# Recurrent *UBE3C-LRP5* translocations in head and neck cancer with therapeutic implications

**DOI:** 10.1038/s41698-024-00555-4

**Published:** 2024-03-04

**Authors:** Bhasker Dharavath, Ashwin Butle, Akshita Chaudhary, Ankita Pal, Sanket Desai, Aniket Chowdhury, Rahul Thorat, Pawan Upadhyay, Sudhir Nair, Amit Dutt

**Affiliations:** 1 Integrated Cancer Genomics Laboratory, Advanced Centre for Treatment, Research, and Education in Cancer, Kharghar, Navi Mumbai, Maharashtra 410210 India; 2 Homi Bhabha National Institute, Training School Complex, Anushakti Nagar, Mumbai, Maharashtra 400094 India; 3 Department of Biochemistry, All India Institute of Medical Sciences, Nagpur, Maharashtra 441108 India; 4 Laboratory Animal Facility, Advanced Centre for Treatment, Research and Education in Cancer, Kharghar, Navi Mumbai, Maharashtra 410210 India; 5 Division of Head and Neck Oncology, Department of Surgical Oncology, Tata Memorial Hospital, Tata Memorial Centre, Parel, Mumbai, 400012 India; 6 Present Address: Department of Genetics, University of Delhi South Campus, New Delhi, 110021 India

**Keywords:** Oral cancer, Oncogenes

## Abstract

Head and neck cancer is a major cause of morbidity and mortality worldwide. The identification of genetic alterations in head and neck cancer may improve diagnosis and treatment outcomes. In this study, we report the identification and functional characterization of *UBE3C-LRP5* translocation in head and neck cancer. Our whole transcriptome sequencing and RT-PCR analysis of 151 head and neck cancer tumor samples identified the *LRP5-UBE3C* and *UBE3C-LRP5* fusion transcripts in 5.3% of patients of Indian origin (*n* = 151), and *UBE3C-LRP5* fusion transcripts in 1.2% of TCGA-HNSC patients (*n* = 502). Further, whole genome sequencing identified the breakpoint of *UBE3C-LRP5* translocation. We demonstrate that *UBE3C-LRP5* fusion is activating in vitro and in vivo, and promotes the proliferation, migration, and invasion of head and neck cancer cells. In contrast, depletion of *UBE3C-LRP5* fusion suppresses the clonogenic, migratory, and invasive potential of the cells. The *UBE3C-LRP5* fusion activates the Wnt/β-catenin signaling by promoting nuclear accumulation of β-catenin, leading to upregulation of Wnt/β-catenin target genes, *MYC, CCND1, TCF4*, and *LEF1*. Consistently, treatment with the FDA-approved drug, pyrvinium pamoate, significantly reduced the transforming ability of cells expressing the fusion protein and improved survival in mice bearing tumors of fusion-overexpressing cells. Interestingly, fusion-expressing cells upon knockdown of *CTNNB1*, or *LEF1* show reduced proliferation, clonogenic abilities, and reduced sensitivity to pyrvinium pamoate. Overall, our study suggests that the UBE3C-LRP5 fusion is a promising therapeutic target for head and neck cancer and that pyrvinium pamoate may be a potential drug candidate for treating head and neck cancer harboring this translocation.

## Introduction

Gene fusion is caused by chromosomal rearrangements or aberrant splicing mechanisms^1,2^. They frequently affect genes that play a role in oncogenesis, such as tyrosine kinases, chromatin regulators, and transcription factors^1^. Although fusion transcripts are challenging to identify, their presence in cancer cells often indicates a greater degree of oncogenic dependency, making them attractive therapeutic targets^3^. The US Food and Drug Administration granted Breakthrough Therapy Designation to several agents targeting the fusion protein and approved them for the treatment of solid tumors harboring fusion genes such as *NTRK*, *ALK*, *RET*, *ROS1*, *BRAF*, and *RAF1*, among others. Larotrectinib and entrectinib, for instance, target NTRK fusions, selpercatinib targets RET fusions, and dabrafenib and trametinib target BRAF fusions^4–11^. The significance of identifying and targeting gene fusions in the treatment of solid tumors has been highlighted by these tumor-independent therapies. The discovery of oncogenic gene fusions has led to the development of tissue-agnostic treatment strategies that have increased cancer patient survival^12^.

Among solid tumors, head and neck squamous cell carcinoma (HNSCC) is one of the most common cancers in developing countries and the sixth most common cancer worldwide^13,14^. Despite the high burden of genomic aberrations in HNSCC, the landscape of fusion genes remains largely unexplored^15^. The identification of therapeutically relevant fusion genes in HNSCC could help in the development of targeted therapies for patients regardless of the tumor subsite. There are few reports on fusion genes in HNSCC, such as *FGFR3-TACC3* fusion^2^ and *MYB-NFIB* fusion. In vitro studies have shown that *FGFR3-TACC3* fusion^16^ could play a role in resistance to *EGFR/ERBB3* inhibition in HNSCC^17^. There are other reports on gene fusions in HNSCC samples^18,19^ and cell lines^20^, but they lack clinical data, and no drugs have been approved against fusion genes as targeted therapies for head and neck cancer. Therefore, identifying and characterizing therapeutically relevant fusion transcripts in HNSCC could help to develop targeted therapies for this cancer.

In this study, we identified a recurrent, and therapeutically relevant inter-chromosomal fusion, *UBE3C-LRP5*, through transcriptome sequencing and RT-PCR-based analysis of HNSCC primary tumor samples. The fusion transcript constitutively activates the Wnt/β-catenin pathway, which promotes proliferation, migration, and invasion of HNSCC cell lines. The fusion was found to be transforming in vitro and in vivo, and responded to an FDA-approved anthelminthic drug, pyrvinium pamoate. Targeting the fusion protein with pyrvinium pamoate represents a potential therapeutic approach for managing patients with head and neck cancer. The identification and characterization of *UBE3C-LRP5* translocation in HNSCC could provide new insights into the biology of the disease and aid in the development of targeted therapies for patients with this cancer.

## Results

### Identification and validation of *LRP5-UBE3C* and *UBE3C-LRP5* translocations in head and neck cancer

We performed whole transcriptome sequencing of five adjacent normal, ten head and neck tumor samples, and four head and neck cancer cell lines (Supplementary Fig. S1) to generate an average of 25 and 34 million paired-end reads, respectively. Using Chimerascan, we performed fusion analysis and identified a total of 242 unique somatic fusion transcripts (Supplementary Figs. S2a, b, S3a–d and Supplementary Table S6). We compared our data with fusion databases (detailed in Materials and Methods) and observed 47 unique transcript fusions overlap, showing identical fusion transcript pairs (Supplementary Table S7).

Among 242 potential fusion transcripts, 12 high-confidence fusion transcripts, including five previously unreported fusion transcripts, were selected for validation using Sanger sequencing based on the following criteria: the presence of an appropriate donor (5’) and acceptor (3’) relationship, the presence of reads spanning the junction region, and those recurrent across multiple samples or expressed at a very high level (Supplementary Table S8). This validation process was performed across the first validation set comprising 44 primary head and neck tumors, which notably included the ten tumors previously subjected to transcriptome sequencing (Supplementary Figs. S1, S4 and Supplementary Table S9), alongside four head and neck cancer cell lines. *CLN6-CALML4* (9/48), *LRP5-UBE3C* (7/48), *RRM2-C2orf48* (7/48), *YIF1A-RCOR2* (6/48), *POLA2-CDC42EP2* (4/48)*, SLC39A1-CRTC2* (2/48), *BACH1-GRIK1* (2/48), *EXT1-MED30* (2/48), fusions transcripts were found to be recurrent, whereas *NAIP-GTF2H2B, PSMD5-VAV2, CTSC-RAB38,* and *FTSJD2-BTBD9* fusion transcripts were observed as non-recurrent (Supplementary Fig. S5a, b). We further prioritized the potential somatic fusion transcripts for functional characterization based on the following criteria: recurrence in patient samples; presence in at least one cell line, which can serve as a model system for genetic perturbation experiments; not reported previously in any fusion databases or literature; and, one of the gene partners in the fusion transcript is reported to play an important role in cancer. Based on these criteria, we prioritized the *LRP5-UBE3C* fusion transcript for functional characterization in head and neck cancer. *LRP5* is a transmembrane low-density lipoprotein receptor involved in the Wnt/β-catenin signaling pathway^21^, and *UBE3C* is an E3 ubiquitin ligase that transfers ubiquitin from the E2 ubiquitin-conjugating enzyme to the substrate^22^.

*LRP5-UBE3C* is an inter-chromosomal fusion transcript of *LRP5* on chromosome 11q13.2 and *UBE3C* on chromosome 7q36.3 in the NT-8e cell line and six of head and neck cancer patients. In the transcriptome sequencing data of the NT-8e cell line, we identified two variants of the *LRP5-UBE3C* fusion transcript, *LRP5-UBE3C* (v1) and *LRP5-UBE3C* (v2) (Supplementary Fig. S6a–e). The fusion transcript variants were validated by RT-PCR and Sanger sequencing in the NT-8e cell line cDNA (Supplementary Fig. S7a–c). In the six patient samples, only one *LRP5-UBE3C* (v1) variant was detected (Supplementary Fig. S8a, b).

**Fig. 1:**
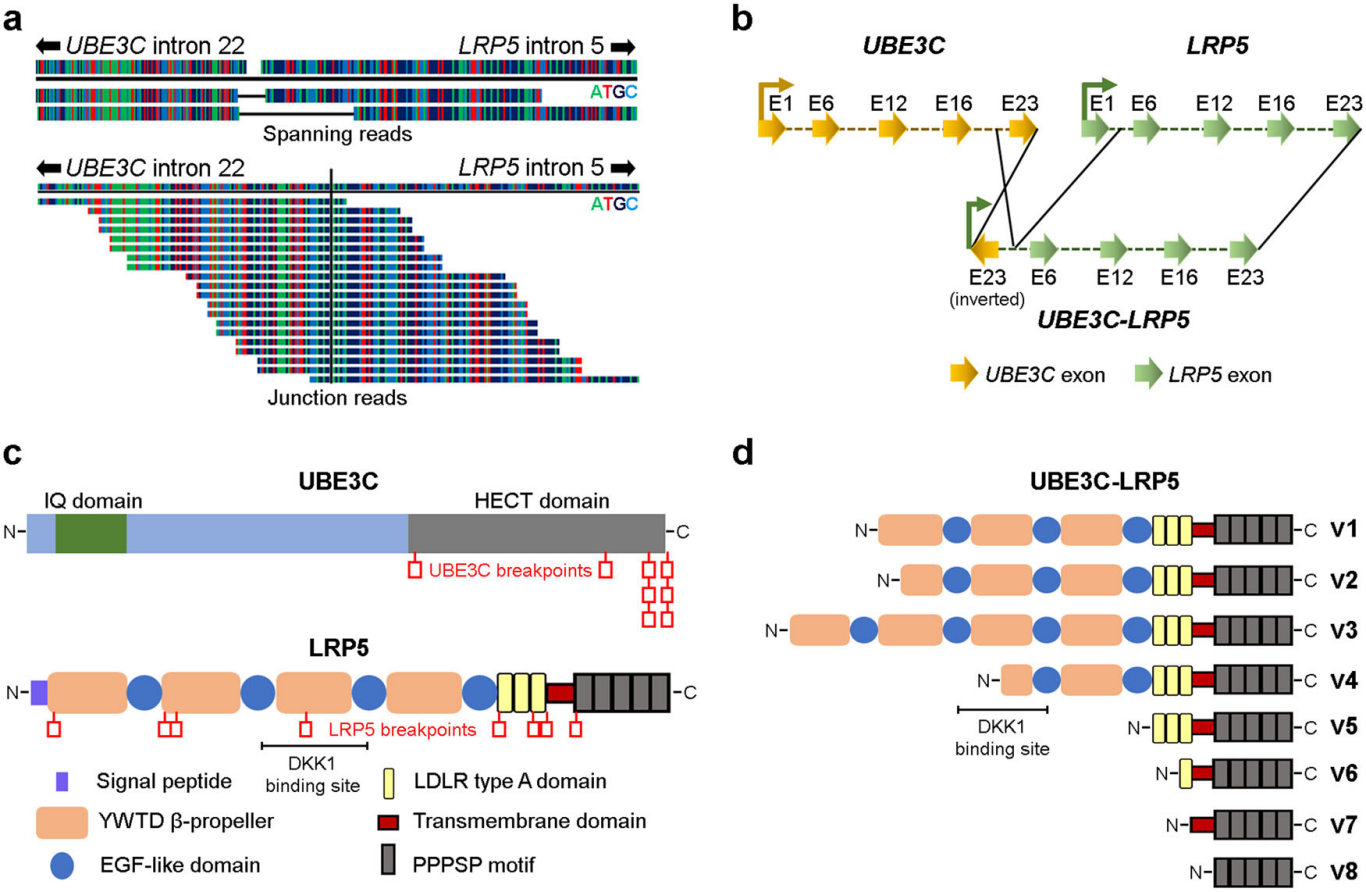
Identification of *UBE3C-LRP5* fusion variants in head and neck cancer. **a** Spanning and junction read supporting the *UBE3C-LRP5* translocation breakpoint at genomic DNA level, as detected by whole genome sequencing of the NT-8e cell line. **b** Schematic representation of *UBE3C-LRP5* fusion depicting inversion of *UBE3C* fusion partner in the fusion. Arrows represent the exons (E1–E23) along with the directionality of the gene. **c** Schematic representation of UBE3C and LRP5 protein domains and all the breakpoints identified. Red boxes indicate breakpoints identified in the NT-8e cell line, in-house HNSCC patient samples, and TCGA-HNSC data. LRP5 protein domains and the DKK1 binding site are mentioned in the schematic image. **d** Schematic representation of the predicted protein domains of the UBE3C-LRP5 (v1–v8) fusion variants identified in the NT-8e cell line, in-house HNSCC samples, and TCGA-HNSC data. The DKK1 binding site is mentioned in the schematic image.

Next, we investigated whether *LRP5* and *UBE3C* fuse at the genomic DNA level to form *LRP5-UBE3C* fusion transcript variants. To identify the translocation breakpoint, we performed whole genome sequencing of the NT-8e cell line (Supplementary Fig. S1) to generate 800 million raw reads, with 35X coverage and analyzed the translocation breakpoint using SvABA and MANTA structural variant analysis tools. Interestingly, we found a unique contig of 400 bp mapping to *LRP5* and *UBE3C* genes. Mapping the junction and spanning reads to the human genome suggested that the translocation breakpoint is located in intron 22 of *UBE3C* and intron 5 of *LRP5* at the genomic DNA level (Fig. 1a). It is noteworthy to mention that of the 10.5 kb intron 22 of *UBE3C*, only 761 bases were retained, and of the 20 kb intron 5 of *LRP5*, only 2.3 kb was retained in the fusion gene at the genomic DNA level. Surprisingly, the *UBE3C* part of the fusion was found to be an inverted 5’-partner gene, suggesting a new fusion variant, *UBE3C-LRP5* (Fig. 1b). To validate the *UBE3C-LRP5* translocation breakpoint and its directionality at the genomic DNA level, orthologous techniques, PCR, and Sanger sequencing were used. PCR and Sanger sequencing analyses confirmed the *UBE3C-LRP5* translocation breakpoint and inversion of the *UBE3C* part at the genomic DNA level (Supplementary Fig. S9a–c).

Further, we investigated whether the *UBE3C-LRP5* translocation transcribed a fusion transcript. Of note, we missed to find spanning/junction reads supporting this fusion in the transcriptome sequencing data of the NT-8e cell line, potentially attributable to the limited coverage of 28 million paired-end reads. We designed primers and performed RT-PCR to identify the *UBE3C-LRP5* fusion transcript in the cDNA of NT-8e cells. Surprisingly, RT-PCR-based amplification of the *UBE3C-LRP5* fusion transcript displayed two distinct bands on agarose gel (Supplementary Fig. S10a). Sanger sequencing of the bands identified two variants of *UBE3C-LRP5* fusion (*UBE3C-LRP5* (v1) and *UBE3C-LRP5* (v2)) in the NT-8e cell line (Supplementary Fig. S10b, c). The *UBE3C-LRP5* (v1) variant is a fusion of inverted exon 23 of *UBE3C*, with 12 bp of retained intron 5 of *LRP5* and exon 6–23 of *LRP5*. The *UBE3C-LRP5* (v2) variant is a fusion of inverted exon 23 of *UBE3C* and exon 6–23 of *LRP5* with a deletion of 188 bases from the start of exon 6 of *LRP5*. To examine the presence of both *LRP5-UBE3C* (v1, v2), and *UBE3C-LRP5* (v1, v2) fusion variants, we performed RT-PCR analysis on the cDNA from a total of 151 head and neck tumor samples. This set included the 44 tumor samples from the first validation set (Supplementary Figs. S1, S4 and Supplementary Table S9). We identified *LRP5-UBE3C* (v1) fusion in 4% (6/151) and *UBE3C-LRP5* (v2) fusion in 2.6% of (4/151) head and neck cancer patients (Supplementary Figs. S8a, b, S11a, b). Importantly, we also validated the UBE3C-LRP5 fusion protein in the NT-8e cell line using western blotting (Supplementary Fig. S12). Unfortunately, we were unable to conduct similar validation for primary tumors derived from tongue cancer patients due to the limited availability of samples.

To explore whether the fusion is present in head and neck cancer patients of Caucasian origin, we analyzed TCGA-HNSC transcriptome sequencing data (*n* = 502) (Supplementary Fig. S1). Interestingly, we found six different *UBE3C-LRP5* fusion transcript variants (v3–v8) in six (6/502) TCGA-HNSC patient samples (1.2%). Importantly, all predicted UBE3C-LRP5 fusion proteins (v1–v8) retain the functional cytoplasmic domain of LRP5 (Fig. 1c, d). As the *UBE3C* gene is inverted in the *UBE3C-LRP5* fusion, the protein domains of UBE3C are not present in the fusion protein (Fig. 1b, d). We identified two *LRP5-UBE3C* and eight *UBE3C-LRP5* fusion transcript variants. LRP5-UBE3C fusion variant proteins are predicted to harbor the extracellular Wnt binding domain of LRP5, whereas UBE3C-LRP5 fusion variant proteins are predicted to harbor the transmembrane and cytoplasmic signal transduction domains of LRP5 (Fig. 1c, d and Supplementary Fig. S6b, c).

### *UBE3C-LRP5* fusion stabilizes nuclear β-catenin and constitutively activates the Wnt/β-catenin pathway

**Fig. 2:**
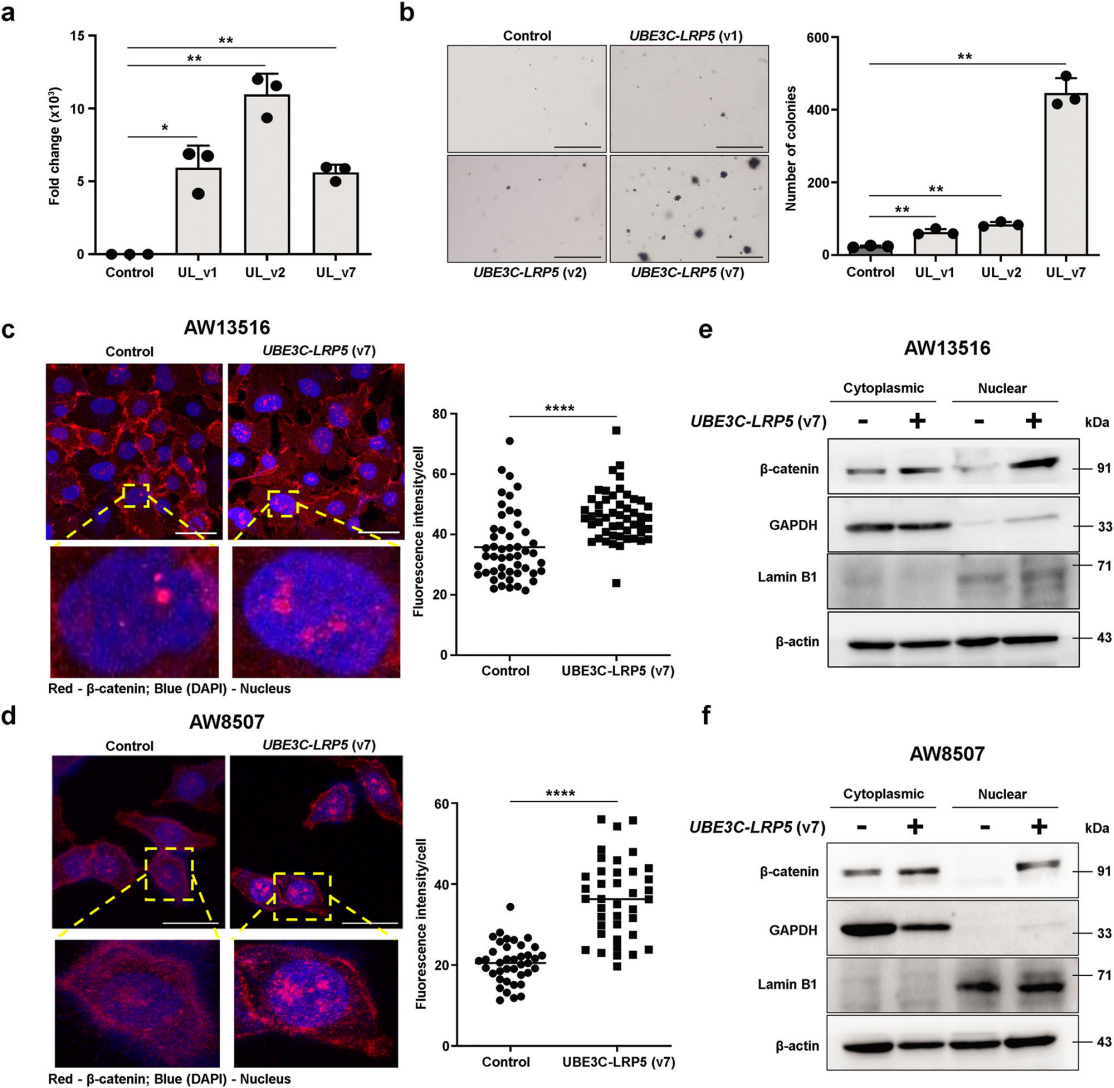
*UBE3C-LRP5* fusion stabilizes nuclear β-catenin to activate the Wnt pathway. **a** qRT-PCR of *UBE3C-LRP5* fusion transcript in NIH/3T3 cells stably overexpressing empty vector or *UBE3C-LRP5* (UL) fusion variants (UL_v1 or UL_v2 or UL_v7). *Gapdh* was used for the normalization of gene expression. **b** Soft agar anchorage-independent growth assay of NIH/3T3 cells stably overexpressing the empty vector or fusion transcript variants. The bar plot indicates the number of colonies in fusion overexpression clones and vector control cells. Scale bar = 400 µM. **c**, **d** Schematic representation of immunofluorescence assay performed to detect expression of nuclear β-catenin in AW13516 (**c**) and AW8507 (**d**) clones stably overexpressing empty vector or *UBE3C-LRP5* (v7) fusion. The yellow color dotted box on the images represent the magnified area of the image shown below. The nucleus is stained with DAPI (blue color) and β-catenin is stained with Alexa Fluor 633 (red color). The scatter plot representation of immunofluorescence assay data on the right indicates the fluorescence intensity/cell (*n* = 50 cells/group). Scale bar = 4 µM (**c**) and 30 µM (**d**). **e**, **f** Immunoblot of β-catenin, GAPDH, Lamin B1, and β-actin in the cytoplasmic and nuclear fractions of AW13516- (**e**) and AW8507- (**f**) clones stably expressing empty vector or *UBE3C-LRP5* (v7) fusion. The blots are from the same experiment and were processed in parallel. Data were shown as means ± SD. *p* values are from the Student’s unpaired *t*-test and denoted as ns (not significant); *p < 0.05; **p < 0.01; ****p < 0.0001. The data shown are representative of *n* = 3 independent experiments.

To study if *LRP5-UBE3C* and *UBE3C-LRP5* fusion variants are activating, we stably overexpressed *LRP5-UBE3C* (v1) or *UBE3C-LRP5* (v1) or *UBE3C-LRP5* (v2) or *UBE3C-LRP5* (v7) fusion variants in the NIH/3T3 cell line. Overexpression of the fusion variants was confirmed by real-time PCR (Fig. 2a and Supplementary Fig. S13a). We performed a soft agar anchorage-independent growth assay with NIH/3T3 clones overexpressing fusion variants or empty vectors to check the transforming potential of the fusion transcript variants. Overexpression of *UBE3C-LRP5* (v7) was found to be the most activating, with a high number of soft agar colonies, compared to other *LRP5-UBE3C* (v1) and *UBE3C-LRP5* (v1, v2) fusion variants (Fig. 2b and Supplementary Fig. S13b). Here, we prioritized the *UBE3C-LRP5* (v7) fusion variant for functional characterization, which is referred to as the “*UBE3C-LRP5*” fusion in the manuscript. To decipher the role of the *UBE3C-LRP5* fusion in head and neck cancer, we stably overexpressed the fusion variant in the head and neck cancer cell lines, AW13516 and AW8507. Overexpression of the fusion was confirmed by real-time PCR and Western blotting (Supplementary Fig. S14a–c). As *LRP5* is a Wnt co-receptor that stabilizes β-catenin upon activation^23,24^, we asked if the *UBE3C-LRP5* (v7) fusion variant constitutively activates the Wnt pathway by stabilizing nuclear β-catenin. We performed an immunofluorescence assay to quantify the expression levels of nuclear β-catenin upon overexpression of *UBE3C-LRP5* in the AW13516 and AW8507 cell lines. The results suggest a significant increase in nuclear β-catenin expression upon overexpression of the fusion in both the head and neck cancer cell lines (Fig. 2c, d). To confirm these findings, we fractionated AW13516 and AW8507 cells, stably overexpressing *UBE3C-LRP5* fusion or vector control, and separated the nuclear and cytoplasmic fractions. Western blotting results suggested a significantly high accumulation of β-catenin in the nuclear fraction of cells upon overexpression of *UBE3C-LRP5* fusion compared to vector control cells (Fig. 2e, f). These results suggest the constitutive activation of the Wnt/β-catenin pathway. To confirm whether the pathway was activated, we screened for the expression of Wnt signaling pathway target genes (*MYC*, and *CCND1*), and co-activators (*CTNNB1*, *LEF1*, and *TCF4*). Real-time PCR-based validation for expression of these genes in AW13516- and AW8507-*UBE3C-LRP5* fusion overexpression clones showed significantly high expression of *MYC*, *CCND1*, *LEF1*, and *TCF4* upon overexpression of the fusion transcript (Supplementary Fig. S15a, b). These results indicate that *UBE3C-LRP5* fusion stabilizes nuclear β-catenin to constitutively activate the Wnt/β-catenin pathway and transform cells in vitro.

### *UBE3C-LRP5* fusion has transforming potential in vivo and promotes metastasis in vitro

**Fig. 3:**
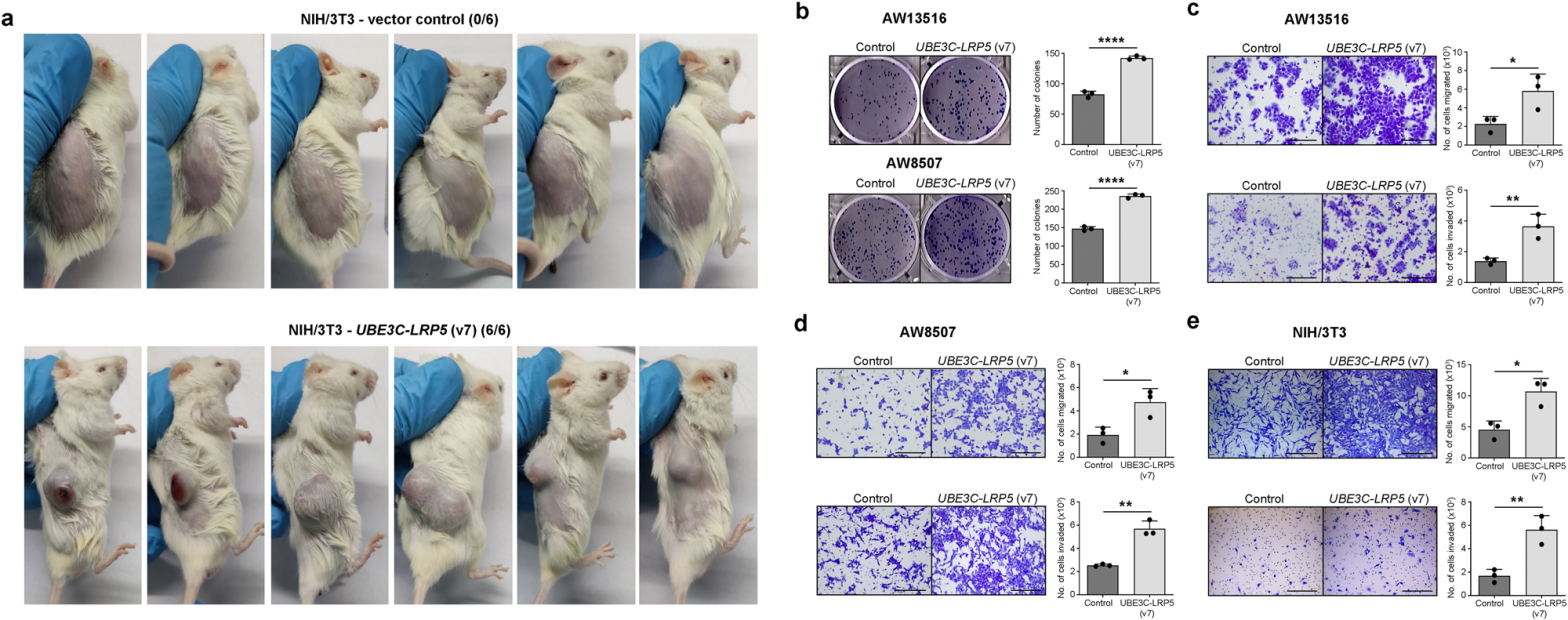
*UBE3C-LRP5* (v7) fusion has transforming potential in vivo and promotes metastasis in vitro. **a** In vivo tumorigenicity assay performed using NOD-SCID mice subcutaneously injected with NIH/3T3 cells stably overexpressing the empty vector (*n* = 6) or *UBE3C-LRP5* (v7) fusion variant (*n* = 6). The number of mice with the tumors is mentioned on the top of the mice images. **b** Clonogenic cell survival assay of AW13516 (top panel) and AW8507 (bottom panel) cells stably expressing empty vector or *UBE3C-LRP5*. The bar plots on the right indicate the number of colonies formed in each group. **c**–**e** Cell migration (top panels) and invasion assay (bottom panels) of AW13516- (**c**), AW8507- (**d**), and NIH/3T3- (**e**) cells stably expressing empty vector or *UBE3C-LRP5* (v7) fusion. The bar plots depict the number of cells migrated or invaded in vector control and *UBE3C-LRP5* (v7) fusion-expressing clones. Data were shown as means ± SD. *p* values are from the Student’s unpaired *t*-test and denoted as *p < 0.05; **p < 0.01; ****p < 0.0001. The data shown are representative of *n* = 3 independent experiments. Scale bar = 400 µM.

**Fig. 4:**
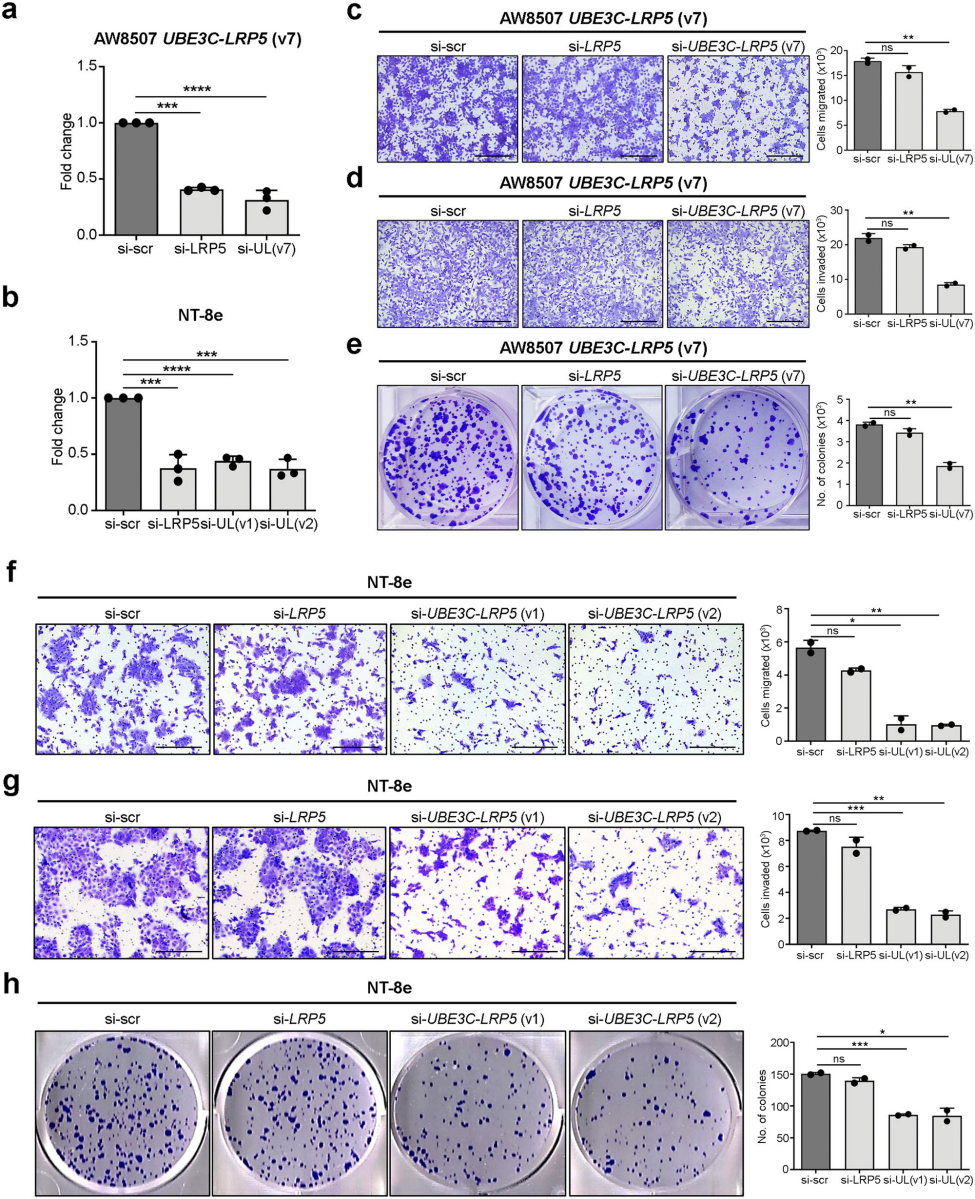
Knockdown of *UBE3C-LRP5* fusion suppresses clonogenic, migratory, invasive potential of head and neck cancer cells. **a** Real-time PCR of *LRP5*, and *UBE3C-LRP5* (v7) fusion variant in AW8507-*UBE3C-LRP5* (v7) clones expressing scrambled siRNA or siRNAs targeting *LRP5*, or *UBE3C-LRP5* (v7). **b** Real-time PCR of *LRP5*, and *UBE3C-LRP5* (v1 and v2) fusion variants in NT-8e cells expressing scrambled siRNA or siRNAs targeting *LRP5*, *UBE3C-LRP5* (v1), or *UBE3C-LRP5* (v2). **c**, **d**, **f**, **g** Boyden chamber cell migration (**c**, **f**) and invasion assay (**d**, **g**) of AW8507-*UBE3C-LRP5* (v7) (**c**, **d**) and NT-8e cells (**f**, **g**) expressing scrambled siRNA or siRNA targeting LRP5 or the *UBE3C-LRP5* fusion variants. The bar plots depict the number of cells migrated or invaded in scrambled siRNA control or siRNAs targeting *LRP5*, or UBE3C-LRP5 fusion variants. **e**, **h** Clonogenic cell survival assay of AW8507-*UBE3C-LRP5* (v7) (**e**) and NT-8e cells (**h**) expressing scrambled siRNA or siRNA targeting the *UBE3C-LRP5* fusion variants or *LRP5*. The bar plots on the right indicate the number of colonies formed in each group. Data were shown as means ± SD. *p* values are from the Student’s unpaired *t*-test and are denoted as *ns* (not significant); *p < 0.05; **p < 0.01; ***p < 0.001; ****p < 0.0001. Scale bar = 400 µM.

To confirm the in vitro findings and study the role of *UBE3C-LRP5* fusion in tumorigenesis in vivo, we used a xenograft mouse model and injected NIH/3T3 clones overexpressing *UBE3C-LRP5*, along with vector control cells, subcutaneously in six mice per group. Caliper measurements at regular intervals showed that NIH/3T3 cells overexpressing the empty vector did not form tumors (0/6), whereas NIH/3T3 cells overexpressing *UBE3C-LRP5* fusion formed tumors in 6/6 mice (Fig. 3a), confirming the transforming potential of fusion transcripts in vitro, and in vivo. The Wnt/β-catenin pathway has been reported to regulate proliferation, migration, and invasion in head and neck cancer^25–28^. We investigated whether the *UBE3C-LRP5* fusion-activated Wnt pathway promoted these phenotypes in head and neck cancer cell lines. A clonogenic assay was performed to assess the ability of single cells to form colonies and the long-term survival of cells overexpressing fusion and vector control cells. Clonogenic assay of AW13516- and AW8507- clones overexpressing *UBE3C-LRP5* fusion, but not the full-length *LRP5*, suggested a significant increase in the number and size of the colonies upon overexpression of the fusion compared to the vector control in both cell lines (Fig. 3b and Supplementary Fig. S16a–e). Assays were performed to investigate the role of the *UBE3C-LRP5* fusion-activated Wnt pathway in metastasis, invasion, and migration. In vitro, migration assay results suggested a significant increase in the migratory potential of AW13516, AW8507, and NIH/3T3 cells upon overexpression of *UBE3C-LRP5* fusion as compared to the vector control cells (Fig. 3c–e). Furthermore, the invasion assay also suggested a significant increase in the invasive potential of AW13516, AW8507, and NIH/3T3 cells upon overexpression of the fusion (Fig. 3c–e), suggesting that *UBE3C-LRP5* fusion overexpression induces cellular proliferation, migration, and invasion. In contrast, siRNA-mediated knockdown of *UBE3C-LRP5* (v7) fusion, but not the full-length *LRP5*, in AW8507-*UBE3C-LRP5* (v7) fusion overexpression clones suppresses the clonogenic, migratory, and invasive potential of the clones (Fig. 4a, c–e and Supplementary Fig. S17). Strikingly, transient knockdown of *UBE3C-LRP5* (v1 and v2) fusion variants in the NT-8e cell line, which endogenously expresses these fusion variants, significantly reduces the clonogenic, migratory, and invasive potential of the cells (Fig. 4b, f–h). Consistently, the knockdown of *LRP5* did not affect these phenotypes in the NT-8e cell line. Taken together, these results suggest that overexpression of *UBE3C-LRP5* fusion, but not *LRP5*, is sufficient to induce tumorigenesis in vitro and in vivo and promote migration and invasion of head and neck cancer cells in vitro.

### Pyrvinium pamoate inhibits *UBE3C-LRP5* activated Wnt pathway and suppresses tumor growth

**Fig. 5:**
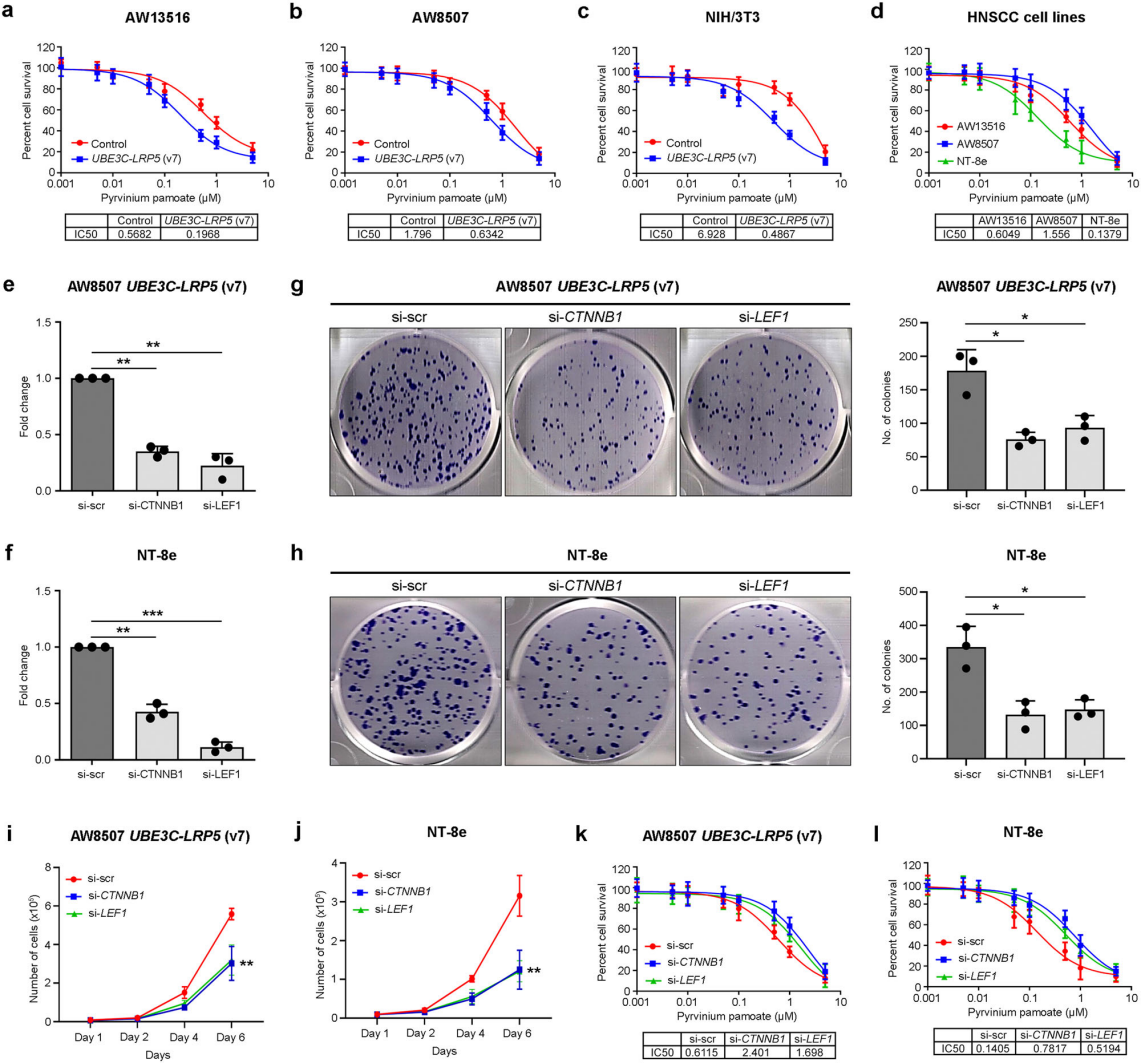
Pyrvinium pamoate treatment or knockdown of *CTNNB1* or *LEF1* inhibits *UBE3C-LRP5* activated Wnt pathway and suppresses proliferation and clonogenic ability of HNSCC cells. **a**–**d** MTT assay for assessing the response of AW13516- (**a**), AW8507- (**b**), and NIH/3T3- (**c**) clones stably expressing empty vector or *UBE3C-LRP5* and HNSCC cell lines (AW13516, AW8507, and NT-8e) (**d**) to pyrvinium pamoate. The percentage of cells surviving the treatment (y-axis) and drug concentration (x-axis) are plotted. The table at the bottom shows IC50 values (µM). **e**, **f** Real-time PCR of *CTNNB1*, and *LEF1* in AW8507-*UBE3C-LRP5* (v7) clones (**e**) and NT-8e (**f**) cells expressing siRNAs targeting *CTNNB1* or *LEF1* compared to the cells expressing scrambled siRNA. **g**, **h** Clonogenic cell survival assay of AW8507-*UBE3C-LRP5* (v7) clones (**g**), and NT-8e cells (**h**) expressing scrambled siRNA or siRNAs targeting the *CTNNB1* or *LEF1*. The bar plots on the right indicate the number of colonies formed in each group. **i**, **j** Cell proliferation assay of AW8507-*UBE3C-LRP5* (v7) clones (**i**) and NT-8e cells (**j**) expressing scrambled siRNA or siRNAs targeting the *CTNNB1* or *LEF1*. Scatter plots indicate the number of live cells on the mentioned day. **k**, **l** MTT assay for assessing the response of AW8507-*UBE3C-LRP5* (v7) clones (**k**), and NT-8e cells (**l**) expressing scrambled siRNA or siRNAs targeting the *CTNNB1*, or *LEF1* to pyrvinium pamoate. Data were shown as means ± SD. *p* values are from the Student’s unpaired *t*-test and denoted as *p < 0.05; **p < 0.01; ***p < 0.001.

**Fig. 6:**
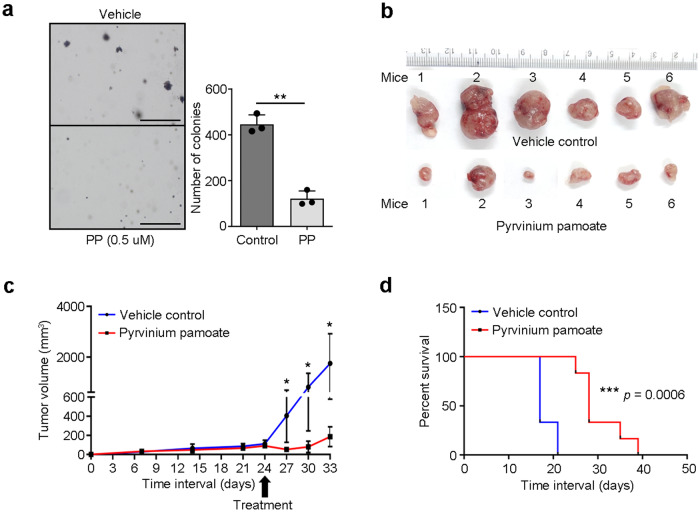
Pyrvinium pamoate treatment suppresses tumor growth and increases survival of mice injected with *UBE3C-LRP5* (v7) fusion-expressing cells. **a** Soft agar anchorage-independent growth assay of NIH/3T3-*UBE3C-LRP5* (v7) clones treated with vehicle or 0.5 µM pyrvinium pamoate (PP). The bar plot indicates the number of colonies in the vehicle and pyrvinium pamoate treatment arm. Data were shown as means ± SD. **b** Excised tumors from mice injected with NIH/3T3-*UBE3C-LRP5* clones, treated with vehicle or pyrvinium pamoate. **c** The graph depicts the tumor volume (y-axis) and number of days (x-axis) after injection of NIH/3T3-*UBE3C-LRP5* clones in the mice. Arrow represents the day of the start of drug treatment. Tumor volumes are shown as mean ± SEM (*n* = 6/group). **d** Kaplan–Meier survival curves for overall survival (OS) of mice treated with vehicle control (blue line) or pyrvinium pamoate (red line). The log-rank test was used to determine the statistical differences in median survival. The *p* values are from the Student’s unpaired *t*-test and denoted as *p < 0.05; **p < 0.01. Scale bar = 400 µM.

To identify therapeutic drugs that target the constitutively activated Wnt/β-catenin pathway, we screened the literature and found that an FDA-approved anthelminthic drug, pyrvinium pamoate, has been reported to target β-catenin and inactivate Wnt signaling in various cancers^29–31^. To determine whether pyrvinium pamoate targets and degrades β-catenin in head and neck cancer, we treated the AW13516 and AW8507 cell lines with increasing doses of pyrvinium pamoate (0.5, 1, 2, and 3 µM) and screened for β-catenin protein expression by Western blotting. Western blotting results suggested a dose-dependent degradation of β-catenin in head and neck cancer cell lines (Supplementary Fig. S18). Furthermore, to study whether the cells overexpressing *UBE3C-LRP5* fusion are sensitive to pyrvinium pamoate, we performed an MTT assay with AW13516, AW8507, and NIH/3T3 clones overexpressing the fusion and compared them to vector control cells. MTT assay results suggested a significant increase in the sensitivity of cell lines overexpressing *UBE3C-LRP5* fusion to pyrvinium pamoate (Fig. 5a–c). We asked if NT-8e cells, which endogenously express the *UBE3C-LRP5* (v1 and v2) fusion variants, display sensitivity to pyrvinium pamoate and performed MTT assay with the HNSCC cell lines (AW13516, AW8507, and NT-8e). Interestingly, NT-8e cells showed higher sensitivity to pyrvinium pamoate compared to other HNSCC cells without the fusion (Fig. 5d). To check the dependency of fusion-expressing cells on the Wnt/β-catenin pathway, we performed siRNA-mediated knockdown of *CTNNB1* and *LEF1*. Strikingly, we find that knockdown of *CTNNB1* or *LEF1* suppresses proliferation, and clonogenic abilities of AW8507-*UBE3C-LRP5* (v7) overexpression clones and NT-8e cells (Fig. 5e–j). Moreover, the cells display decreased sensitivity to pyrvinium pamoate upon knockdown of *CTNNB1* or *LEF1* (Fig. 5k, l), confirming the dependency of fusion-expressing cells on the Wnt/β-catenin pathway. To study the effect of pyrvinium pamoate on soft agar anchorage-independent growth, we performed an assay with NIH/3T3 cells stably overexpressing the *UBE3C-LRP5* fusion and treated them with pyrvinium pamoate (0.5 µM). Soft agar anchorage-independent assay results suggested a significant decrease in the number and size of colonies after drug treatment (Fig. 6a). To confirm the in vitro findings and study the effect of pyrvinium pamoate on tumorigenesis in vivo, we used a xenograft mouse model and subcutaneously injected 12 mice with NIH/3T3 clones overexpressing *UBE3C-LRP5*. Mice were randomly divided into two groups (*n* = 6 mice/group) for pyrvinium pamoate or vehicle treatment. Caliper measurements were taken at regular intervals to monitor the tumor and pyrvinium pamoate drug treatment (10 mg/kg dose every 48 h) was started when the tumors in mice reached 100 mm^3^ in size^32^ in 24 days. In vivo results suggest a significant decrease in the tumor volume upon pyrvinium pamoate treatment (Fig. 6b, c and Supplementary Fig. S19). Remarkably, we noticed a distinct pattern where the tumor volume experiences a significant and abrupt escalation once it reaches the size range of 100–120 mm^3^ in the vehicle control group. Specifically, we observed a remarkable 5–6-fold increase in tumor volume between day 24 and day 27. To generate a visual representation of excised tumors from both the vehicle control and treatment groups, we conducted terminal sacrifice of the mice on day 33 post-injection. Additionally, we sought to assess whether pyrvinium pamoate treatment has a positive impact on the overall survival of the mice. For this purpose, we introduced NIH/3T3 clones overexpressing *UBE3C-LRP5* into a fresh set of 12 mice, which were then randomly divided into two groups, with each group comprising six mice. These groups were subjected to either pyrvinium pamoate or vehicle treatment. The survival study depicts Kaplan–Meier (KM) curves at various time points following cell injection (Fig. 6d). The presence of two distinct steps in the KM curve for the vehicle control group signifies that, in the first step, *n* = 4 mice were terminally sacrificed, while *n* = 2 mice remained in the second step. These terminations were carried out in accordance with the humane endpoint criteria outlined by the Institutional Animal Ethics Committee (IAEC) at ACTREC, which considered both animal health and tumor volume. The survival analysis results suggest that pyrvinium pamoate targets β-catenin and inhibits tumor growth, thereby increasing the survival of mice injected with NIH/3T3 clones overexpressing *UBE3C-LRP5* fusion (Fig. 6d). We investigated whether the presence of fusion transcripts affects the survival of patients with head and neck cancer. Follow-up data were available for 138 in-house HNSCC patients, and the median survival duration for the cohort was 3.3 years (ranges from 1 month to 8.5 years, as the patient samples were collected retrospectively, the follow-up period was not reached the 5-year mark in all patients). During this period, 59 recurrences, 15 distant metastases, and nine fatal events occurred. We performed Kaplan–Meier survival analysis using in-house head and neck cancer data (*n* = 138) and TCGA-HNSC data (*n* = 502). Survival analysis results showed a trend of poor survival in patients positive for fusion in the in-house data (*p* = 0.078) and TCGA-HNSC data (*p* = 0.43); however, this was not significant due to the small number of patients positive for the fusion (Supplementary Fig. S20a, b).

## Discussion

The literature suggests that truncated mutant of *LRP5* with loss of the DKK1 inhibitory domain is activating in hyperparathyroid^33^ and breast cancer^34^. Moreover, *LRP5* and *LRP6* mutants with loss of the extracellular domain have been reported to constitutively activate the Wnt/β-catenin pathway in a Frizzled receptor- and ligand-independent manner^35,36^. Consistent with these reports, ectopic expression of the *UBE3C-LRP5* (v7) splice variant was the most activating, with a higher number of soft agar colonies, compared to other *LRP5-UBE3C* (v1) and *UBE3C-LRP5* (v1, v2) fusion variants, possibly because of the absence of the DKK1 inhibitory domain of LRP5 in the UBE3C-LRP5 (v7) fusion. Immunofluorescence and western blot analysis of nuclear and cytoplasmic cell fractionation revealed a significantly high accumulation of β-catenin in the nuclear fraction of cells upon overexpression of *UBE3C-LRP5* fusion, indicating constitutive activation of the Wnt/β-catenin pathway with upregulated target genes, *MYC*, *CCND1*^37–39^, *LEF1*, and *TCF4*^40–44^ as reported in the literature for multiple cancers. We found that overexpression of *UBE3C-LRP5* fusion significantly increased the clonogenic, migratory, and invasive potential of head and neck cancer cells by activating the Wnt/β-catenin pathway, whereas transient knockdown of the fusion, but not full-length *LRP5*, suppresses these phenotypes. These results are consistent with the literature, which suggests that overexpression or knockdown of full-length *LRP5* does not influence the Wnt pathway activation or transforming ability of cells in breast^34^ and hyperparathyroid cancer^33^. To validate whether the fusion transcript is transforming in vivo, we used xenograft mouse models and subcutaneously injected fusion-expressing NIH/3T3 cells. The *UBE3C-LRP5* fusion transcript transformed NIH/3T3 cells in vivo, forming subcutaneous tumors in 100% of mice injected with cells stably expressing the fusion transcript compared to 0% in mice injected with cells expressing the empty vector. Thus, our findings indicate that *UBE3C-LRP5* fusion activates in vitro and in vivo, promoting proliferation, migration, and invasion of head and neck cancer cells.

Although no drugs have been approved for targeting *LRP5*, we evaluated a known FDA-approved anthelminthic drug, pyrvinium pamoate, which degrades β-catenin to inactivate the Wnt pathway^29–31,45^. Head and neck cancer cells expressing *UBE3C-LRP5* fusion transcripts were sensitive to pyrvinium pamoate, which degrades β-catenin in a dose-dependent manner. Moreover, endogenously fusion-expressing NT-8e cells showed higher sensitivity to pyrvinium pamoate compared to other HNSCC cells without the fusion. Interestingly, the knockdown of *CTNNB1*, or *LEF1*, in fusion-expressing cells reduced the sensitivity of the cells to pyrvinium pamoate, confirming their dependency on the Wnt/β-catenin pathway. Furthermore, soft agar anchorage-independent assay suggested inhibition of the transforming ability of cells upon treatment with the inhibitor, as reported for cells with activated Wnt/β-catenin pathway^46–48^. Consistent with the in vitro findings, in vivo xenograft assay results suggest a significant reduction in the tumor volume of mice bearing tumors of NIH/3T3 cells overexpressing the fusion upon treatment with pyrvinium pamoate as reported in various cancers with activated Wnt/β-catenin pathway, including glioblastoma and breast cancer^30,45,49,50^. Similarly, when treated with pyrvinium pamoate, mice bearing tumors of fusion overexpression clones showed significantly better overall survival than those treated with vehicle control^30^. Importantly, in-house HNSCC data showed a trend towards poor survival of fusion-positive patients, wherein statistical significance could not be attained due to inadequate sample size, similar to the *NTRK* fusions^51^.

In summary, our study presents the functional and clinical significance of a fusion transcript between the E3 ubiquitin ligase, *UBE3C*, and the Wnt signaling co-receptor, *LRP5*, found in a subset of patients with head and neck cancer. The *UBE3C-LRP5* fusion transcript transforms in vitro and in vivo, promoting proliferation, migration, and invasion of head and neck cancer cells through constitutive activation of the Wnt/β-catenin pathway. The UBE3C-LRP5 fusion protein and the Wnt pathway can be targeted by the FDA-approved anthelminthic drug pyrvinium pamoate, which degrades β-catenin to inactivate the pathway, leading to a reduction in the anchorage-independent growth of cells and tumor volume in mice bearing tumors of fusion overexpression clones. Importantly, mice treated with pyrvinium pamoate also showed significantly better overall survival than those treated with vehicle control. Further studies are needed to validate the clinical significance of this fusion in larger patient cohorts and to assess the efficacy and safety of pyrvinium pamoate in patients with head and neck cancer. Overall, this study underscores the importance of continued research on the molecular drivers of cancer and the development of targeted therapies that can improve patient outcomes. Specifically, investigating whether the *UBE3C-LRP5* (v7) fusion variant mimics truncated *LRP5* could yield further valuable insights^33,34^.

The limitations of this study include the low activity of pyrvinium pamoate on head and neck cancer cell lines, its retrospective design, and the lack of samples positive for the fusion transcripts, which led to inconclusive results in the survival analysis. Designing a prospective study with a large cohort of patients is warranted to provide detailed information on fusion gene prevalence, correlation with clinicopathological features and patient survival, and establish *UBE3C-LRP5* fusion as a therapeutic target in head and neck cancer.

## Methods

### Patient sample details

A total of 151 fresh-frozen head and neck tumor samples were used to identify and validate the fusion transcripts. The patient details are provided in Table 1. The patient details include age, gender, anatomic site, TNM tumor stage (as per the 8th edition of AJCC (American Joint Committee on Cancer)/ UICC (Union for International Cancer Control) TNM classification system), nodal status, smoking (representing the patients with habits of smoking tobacco such as cigarettes, cigars, or pipes), chewing, alcohol, tobacco (representing the patients with the habit of smoke-less tobacco chewing), recurrence, metastasis, and status at the last follow-up.

**Table 1:** Clinical characteristics of 151 patients in the study

Clinicopathological features	Variable	Frequency (*n* = 151), *n* (%)
Age	Median (range)	44 (23–76)
	<40	49 (32%)
	40–60	64 (42%)
	>60	25 (17%)
	NA	13 (9%)
Gender	Male	111 (74%)
	Female	27 (18%)
	NA	13 (9%)
Anatomic site	Tongue	150 (99%)
	Buccal mucosa	1 (1%)
TNM Tumor stage	pT1-T2	114 (75%)
	pT3-T4	37 (25%)
Nodal Status	Node positive	85 (56%)
	Node negative	66 (44%)
Smoking	Smoker	40 (26%)
	Non-smoker	98 (65%)
	NA	13 (9%)
Chewing	Chewer	100 (66%)
	Non-chewer	38 (25%)
	NA	13 (9%)
Alcohol	Yes	32 (21%)
	No	106 (70%)
	NA	13 (9%)
Tobacco	Yes	102 (68%)
	No	36 (24%)
	NA	13 (9%)
Recurrence	Yes	59 (39%)
	No	79 (52%)
	NA	13 (9%)
Metastasis	Yes	15 (10%)
	No	123 (81%)
	NA	13 (9%)
Status at last follow-up	Alive	129 (85%)
	Died	9 (6%)
	NA	13 (9%)

*NA* information not available

### Ethical approval

The study was conducted according to the guidelines of the Declaration of Helsinki and approved by the Institutional Review Board (IRB) and Ethics Committee (EC) of the Tata Memorial Centre-ACTREC. All patient samples were collected from the tumor tissue repository (TTR) of Tata Memorial Hospital (TMH) and the biorepository of Advanced Centre for Treatment, Research, and Education in Cancer (ACTREC), Mumbai, which routinely collects and maintains fresh-frozen tumor and normal tissue samples of cancer patients for research use. As the samples were collected retrospectively, the IRB and EC waived the need for informed consent. This is a routine procedure followed at the TMH and ACTREC.

### Cell culture

AW13516^52^, AW8507^52^, NT-8e^53^, and OT9^53,54^ head and neck cancer (HNSCC) cell lines^55,56^ were obtained from Tata Memorial Hospital (Mumbai), while the NIH/3T3 (CRL-1658) cell line was procured from the American Type Culture Collection (ATCC). The cell lines were authenticated by DNA short tandem repeat (STR) profiling using the Promega Geneprint 10 system in conjugation with the GeneMarker HID software tool. HNSCC cell lines were cultured in Dulbecco’s Modified Eagle Medium (DMEM) (cat. no. 12800 – 017; Gibco), supplemented with 10% fetal bovine serum (FBS) (cat. no. 10270106; Gibco) and 1.25 µl/ml gentamycin (Abbott). NIH/3T3 cells were cultured in DMEM supplemented with 10% bovine calf serum (BCS) (cat. no. SH30073.03; Cytiva HyClone) and 1.25 µl/ml gentamycin. Cells were tested for mycoplasma and found to be negative, but as a standard lab protocol, we treated the cells using EZKill Mycoplasma Removal Reagent (cat. no. CCK006 – 1; HiMedia) every 6 months.

### DNA/RNA extraction, RT-PCR, and real-time PCR

RNA was extracted from the tissue samples using the AllPrep DNA/RNA/miRNA Universal Kit (cat. no. 80224; QIAGEN). Approximately 20–30 mg of tissue sections were cut into small pieces, subjected to bead-based homogenization in 600 µL lysis buffer using a FastPrep homogenizer (MP Biomedicals, USA), and further processed for total RNA extraction according to the manufacturer’s protocol. Total RNA was extracted from the cell lines using the TRIzol reagent (cat. no. T9424; Sigma-Aldrich). According to the manufacturer’s instructions, genomic DNA from the cell lines was extracted using the QIAamp DNA Blood Mini Kit (cat. no. 51106; QIAGEN). The RNA/DNA concentration was measured using a NanoDrop 2000c spectrophotometer (Thermo Fisher Scientific). About 2 µg total RNA from tumor and normal tissues was used for cDNA synthesis using the PrimeScript TM first strand cDNA synthesis kit (cat. no. RR370A; TaKaRa) according to the manufacturer’s protocol. About 10 ng of cDNA was used for PCR amplification using a KAPA Taq PCR kit (cat. no. KK1024; KAPA Biosystems) in a 10 µl reaction containing fusion-specific forward and reverse primers or GAPDH primers (2 pmol), followed by gel-purification, and Sanger sequencing. The details of all primers used for the fusion transcript validation are provided in Supplementary Table S1. Quantitative real-time PCR was performed using the KAPA SYBR real-time PCR master mix (cat. no. KK4601; KAPA Biosystems) on a QuantStudio 12 K Flex Real-Time PCR System (cat. no. 4470935; Applied Biosystems). For quantitative real-time PCR of each sample, 6 µL reactions in triplicate were incubated in a 384-well plate at 95 °C for 5 min, followed by 40 cycles of 95 °C for 15 s, 64 °C for 15 s, and 72 °C for 15 s. Primer sequences used for real-time PCR validation of genes are provided in Supplementary Table S2. All real-time PCR experiments were performed in triplicate.

### Transcriptome sequencing and fusion analysis

Transcriptome sequencing of 15 head and neck cancer patient samples and four cell lines was performed to identify the fusion transcripts. Transcriptome sequencing libraries were constructed using the TruSeq RNA library protocol. Briefly, mRNA was purified from 4 μg of intact total RNA using oligodT beads, and library preparation was performed according to the manufacturer’s protocol (TruSeq RNA Sample Preparation Kit, Illumina). Chimerascan^57^ was used to identify transcript fusion following the default parameters in the tumor, normal, and cell lines. For fusion mapping, paired-end raw read sequences were mapped to human reference genome sequences (hg19). Using scripts developed in-house in Python, we filtered fusion pairs without spanning read support, transcript allele fraction (TAF) <0.01, for both the partner, pseudogenes, and homologous sequences spanning reads, as described previously^58^. Tumor-specific fusions were further processed for annotation using Oncofuse^59^, and the frame of fusion was determined. The upstream and downstream sequences supporting fusion were retrieved, and primers were designed using Primer-BLAST^60^.

### TCGA-HNSC data fusion analysis

The RNA-seq tier 1 data (aligned BAM files) of the TCGA-HNSC project (*n* = 502) were downloaded from the National Cancer Institute Cancer Genome Commons Portal (http://portal.gdc.cancer.gov). The BAM files were converted to raw fastq files using the SamToFastq utility of the Picard toolkit (https://broadinstitute.github.io/picard/) as described previously^61^. Primary alignment of the transcriptome data was performed against GRCh38 (GRCh.p12 GENCODE v30) using the two-pass mode of the STAR aligner (v2.7.6a)^62^. Discordant and split reads mapping to *LRP5*/ *UBE3C* genomic coordinates were extracted and annotated using in-house custom scripts. The reads supporting the breakpoints were manually superimposed to derive contigs.

### Whole genome sequencing and SvABA analysis

The whole genome sequencing (WGS) library was prepared using the Illumina-compatible NEXTFlex Rapid DNA Sequencing Bundle (5144 – 02, NEXTFlex, BIOO Scientific, Inc. USA). Briefly, 300 ng of Qubit-quantified DNA was sheared in a Covaris microTUBE AFA (520045, Life Technologies) using a Covaris S220 sonicator (4465653, Life Technologies, Covaris, Inc. USA) to generate fragments with a size range of 300–400 bp. Fragment size distribution was verified using an Agilent 2100 Bioanalyzer and purified using HighPrep magnetic beads (AC-60050, MagBio Genomics, Inc., USA). Purified fragments were end-repaired, adenylated, and ligated to Illumina multiplex barcode adapters according to the NEXTFlex Rapid DNA sequencing bundle kit protocol. The adapter-ligated DNA was purified using HighPrep beads. The resultant fragments were amplified for five cycles of PCR using Illumina-compatible primers provided in the NEXTflex Rapid DNA sequencing Bundle. The final PCR product (sequencing library) was purified using HighPrep beads, followed by quality control of the library. The sequencing library was quantified using a Qubit fluorometer (Thermo Fisher Scientific, MA, USA), and the fragment size distribution was analyzed using an Agilent 2100 Bioanalyzer (5067 – 4626, Agilent High Sensitivity DNA Bioanalyzer Kit). Whole genome sequencing data of the NT-8e cell line were aligned to the human reference genome (GRCh38.p12 GENCODE v30) using BWA-MEM (v0.7.17)^63^. The BAM files were further analyzed using SvABA (v1.1.3)^64^ to identify translocation breakpoints. The translocation breakpoints were annotated using custom scripts, using the reference GENCODE GTF (v30)^65^. The translocation breakpoints identified in the SvABA analysis were additionally confirmed using MANTA (v1.6.0)^66^.

### Protein extraction and western blotting

Cells were lysed using radioimmunoprecipitation assay (RIPA) lysis buffer (cat. no. R0278; Sigma-Aldrich) containing a protease inhibitor cocktail (cat. no. P8340; Sigma-Aldrich) and 0.1 M DTT. After intermittent tapping and vortexing of the samples on ice for 30 min, cell debris was pelleted by centrifugation at 14,000 rpm for 40 min, and the supernatant was collected. For Western blotting, 50 µg of protein was loaded onto an SDS-PAGE gel and transferred onto a PVDF membrane (cat. no. 10600021; Amersham Hybond, GE Healthcare). Blocking was performed using 5% BSA to avoid nonspecific antibody binding. Primary antibodies were diluted in 3% BSA and incubated overnight at 4 °C. The primary antibodies against LRP5 (cat. no. sc-390267; 1:1000; Santa Cruz Biotechnology), DYKDDDDK tag (cat. no. 8146; 1:2000; Cell Signaling Technology), β-Tubulin (cat. no. 2128; 1:2000; Cell Signaling Technology), β-catenin (cat. no. ab32572; 1:2000; Abcam), Lamin B1 (cat. no. sc-374015; 1:1000; Santa Cruz Biotechnology), GAPDH (cat. no. sc-32233; 1:2000; Santa Cruz Biotechnology), Vinculin (cat no. 4650; 1:1000; Cell Signaling Technology), and β-actin (cat. no. sc-47778; 1:2000; Santa Cruz Biotechnology) were used for Western blotting. The membranes were then washed with 1X TBST and incubated with goat anti-rabbit HRP labeled (cat. no. sc-2004; 1:2000; Santa Cruz Biotechnology) or goat anti-mouse HRP labeled (cat. no. sc-2005; 1:2000; Santa Cruz Biotechnology) antibodies for 1 h at room temperature (RT) followed by washing with TBST thrice for 15 min. The ECL Western Blotting Substrate (cat. no. T7101A; TAKARA) was used to visualize luminescence using the Chemidoc system (Bio-Rad). Nuclear and cytoplasmic fractionation was performed as described previously^67^, and Western blotting was performed using 50 µg protein from each fraction. All Western blot analyses were performed in triplicate. The uncropped raw western blot images (Supplementary Fig. S21–S28) are provided in the Supplementary Information.

### Cloning of *LRP5-UBE3C* and *UBE3C-LRP5* fusion cDNA

cDNA of *LRP5-UBE3C* (v1) and *UBE3C-LRP5* (v1, v2, and v7) fusion transcript variants were amplified from the NT-8e cell line using KAPA Taq DNA polymerase (cat. no. KK1024; Sigma-Aldrich) and cloned into the pTZ57R/T cloning vector (InsTAclone PCR cloning kit, cat. no. K1214R; Thermo Fisher Scientific) or pJET 1.2 blunt cloning vector (CloneJET PCR cloning kit, cat. no. K1231; Thermo Fisher Scientific), respectively, as per manufacturer’s protocol. Full-length fusion cDNA sequences (Supplementary Table S3) were confirmed by Sanger sequencing. *LRP5-UBE3C* (v1), *UBE3C-LRP5* (v1, v2) fusion variants were sub-cloned into a retroviral expression vector, pBABE-puro (Addgene plasmid #1764)^68^, and *UBE3C-LRP5* (v7) fusion variant was sub-cloned into a lentiviral expression vector, pLVX-AcGFP1-N1 (plasmid was obtained as a kind gift from Dr. Sagar Sengupta, NII, New Delhi), using restriction digestion-based cloning. The start and stop codons of the fusion transcript cDNAs are highlighted in green and red color, respectively, Supplementary Table S3. Primers used for cloning are listed in Supplementary Table S4.

### siRNA synthesis by in vitro transcription

Sense and anti-sense DNA oligonucleotides for *UBE3C-LRP5* fusion variants (v1, v2, and v7), *LRP5, CTNNB1, LEF1*, and scrambled siRNA (Supplementary Table S5) were ordered from Sigma-Aldrich. The protocol for the synthesis of small RNA transcripts using T7 RNA polymerase is reported in the literature^69^. For each in vitro transcription (IVT) reaction, 1 nmol of each oligonucleotide (re-suspended in 1X TE buffer (10 mM Tris-HCl pH 8.0 and 1 mM EDTA)) was annealed using thermocycler to obtain double-stranded DNA (dsDNA). The thermocycler conditions used were: 95 °C for 3 min, followed by 70 cycles of 95 °C for 30 s (−1 °C/cycle). In vitro transcription (IVT) reaction was performed in 20 µL of a reaction containing 1X T7 transcription buffer (cat. no. P118B, Promega), 1X biotin RNA labeling mix (cat. no. 11685597910, Sigma-Aldrich), 1 U RiboLock RNase Inhibitor (cat. no. EO0381, Fermentas), 10 U T7 RNA polymerase (cat. no. P2075, Promega), and 1 nmol of dsDNA, as a template. The reaction was incubated at 37 °C for 2 h. Sense and anti-sense small interfering RNA (siRNA) synthesized in separate reactions were annealed by mixing the transcription reactions at 95 °C for 1 min, followed by 70 cycles of 95 °C for 30 s (−1 °C/cycle) to obtain double-stranded siRNAs.

### Overexpression and knockdown studies

For stable overexpression of *LRP5-UBE3C* (v1) fusion, the pBABE-puro-*LRP5-UBE3C* construct was used. For stable overexpression of *UBE3C-LRP5* fusion variants (v1, v2, and v7), the constructs cloned in pBABE-puro or pLVX-AcGFP1-N1 were used. Cells with the empty vector were used as a control for overexpression. For retroviral production, 293FT cells were seeded in a six-well plate, a day before transfection, and each of the retroviral constructs, along with the packaging vector, pCL-Ampho, were transfected using Lipofectamine kit (cat. no. L3000 – 015; Invitrogen). For lentiviral production, 293FT cells were seeded in a six-well plate, a day before transfection, and the lentiviral construct, along with the packaging vectors, VSV-G and psPAX2, were transfected using a Lipofectamine kit. The viral soup was collected 48 and 72 h post-transfection, and passed through the 0.45 μM filter for removal of cells/cell debris. Target cells for transduction were seeded 1 day before transduction in a six-well plate and allowed to grow to reach 50–60% confluency. One milliliter of the virus soup (1:1 dilution) and 8 μg/ mL of polybrene (cat. no. H9268, Sigma-Aldrich) was added to cells and incubated for 6 h. Positive clones were selected using 0.5 µg/ml of puromycin treatment (cat. no. TC198; Himedia). For transient overexpression of *LRP5*, pcDNA3.3 ss-3xFLAG-hLRP5 plasmid was used, which was a gift from Harald Junge (Addgene plasmid # 115788)^70^. Transient transfection was performed by using a Lipofectamine kit and cells were collected after 48 h for RNA isolation, protein extraction, and to perform cell-based assays. siRNA-mediated transient knockdown of *UBE3C-LRP5* (v1, v2 and v7), *LRP5*, *CTNNB1*, or *LEF1* was performed with siRNAs synthesized using T7 RNA polymerase. Following siRNA transfection for 48 h using Lipofectamine RNAiMAX (cat. no. 13778075, Invitrogen), cells were used for RNA isolation and in vitro cell-based assays.

### Boyden chamber invasion and migration assay

Boyden chamber Matrigel invasion assays were performed using 24-well Transwell inserts (cat. no. 353097; Corning) coated with 100 μg Matrigel (cat. no. 354234; Corning) and allowed to settle for 16 h at 37 °C in 5% CO_2_ incubator. Invasion assay was performed with 2 × 10^5^ cells of AW13516-*UBE3C-LRP5* (v7) clones; 1 × 10^5^ cells of AW8507-*UBE3C-LRP5* (v7) clones, and AW8507-*UBE3C-LRP5* (v7) clones with siRNA knockdown; 0.5 × 10^5^ cells of NIH/3T3-*UBE3C-LRP5* (v7) clones; and 1 × 10^5^ cells of NT-8e with siRNA knockdown suspended in 300 μL serum-free medium and seeded in the Boyden chamber and 700 μL of 10% serum-containing DMEM medium was added in the companion plate wells. For the migration assay, cells were seeded directly in a Boyden chamber without matrigel. For migration assay, 1×10^5^ cells of AW13516-*UBE3C-LRP5* (v7) clones; 0.5 × 10^5^ cells of AW8507-*UBE3C-LRP5* (v7) clones, and AW8507-*UBE3C-LRP5* (v7) clones with siRNA knockdown; 0.25 × 10^5^ cells of NIH/3T3-*UBE3C-LRP5* (v7) clones; and 0.5 × 10^5^ cells of NT-8e with siRNA knockdown were used. The cells were allowed to invade/migrate for 48 h at 37 °C in a 5% CO_2_ incubator. The transwell chambers were fixed and stained with 0.1% crystal violet. The membrane was mounted using DPX mountant (cat. no. 18404; Qualigens) on a slide, the invaded/migrated cells were imaged using an upright microscope at 10X magnification. Images from ten random fields were chosen, and the number of cells was counted using the ImageJ cell counter plugin tool and plotted as percent cell invasion or percent cell migration. All experiments were independently repeated thrice with two inserts per clone in each experiment (a total of six replicates per clone).

### Clonogenic survival assay

Two hundred and fifty cells per well were seeded in a six-well plate (in triplicates for each clone) and incubated for 15–18 days till colonies with >50 cells per colony appeared. Colonies were fixed with 4% paraformaldehyde, stained with 0.1% crystal violet, and counted under the microscope to determine percent survival. All experiments were performed in triplicate.

### Cell proliferation assay

Cell proliferation assay was performed in triplicates in 24-well plates with 10,000 cells/well for all the experiments. Cell growth was assessed on days 1, 2, 4, and 6 by passaging and counting viable cells by trypan blue staining and using a hemocytometer. All experiments were performed three times, with each experiment performed in triplicates.

### Soft agar colony formation assay

Approximately 1 mL of 2X DMEM supplemented with 20% FBS containing 1 mL of 1.6% agar (to obtain 0.8% agar) was added to the six-well plate as bottom agar and solidified. Cells (1 × 10^4^) were supplemented with 1 mL of 2X DMEM containing 0.8% agar to obtain 0.4% agar and were added to the bottom agar as the top agar. Cells were fed 250 μL of medium every 5 days and incubated for two weeks at 37 ^o^C and 5% CO_2_. Cells were stained with 0.01% crystal violet, and from each well randomly, ten field images were taken using a phase-contrast inverted microscope. Colonies were counted manually.

### Immunofluorescence assay

The cells were seeded on sterile coverslips and incubated for 24 h in a CO_2_ incubator. Cells were washed with 1X PBS and fixed with 4% paraformaldehyde for 15 min at room temperature (RT), washed with 1X PBS, and permeabilized with 0.5% Triton X-100 for 15 min at RT. After subsequent washing with 1X PBS, the cells were incubated for 1 h at 37 °C in 3% bovine serum albumin solution. The cells were then incubated with primary antibody: β-catenin (ab32572, 1:500; Abcam) overnight at 4 °C. After washing with 1X PBS, the coverslips were incubated with Alexa Fluor 633-conjugated goat anti-rabbit secondary antibody (cat. no. A-21070; Invitrogen) for 45 min at RT. Nuclei were counterstained with 0.5 μg/ml DAPI (cat. no. D1306; Invitrogen) for 1 min, washed thrice with 1X PBS, and mounted using VECTASHIELD antifade mounting media (cat. no. H-1000; Vector laboratories). The cells were visualized under a Zeiss LSM 510 Meta Confocal Microscope, and the staining intensities were analyzed using the ImageJ software (NIH, Bethesda, MD).

### Inhibitor studies

The Wnt pathway inhibitor pyrvinium pamoate (cat. no. P0027; Sigma-Aldrich) was reconstituted in dimethyl sulfoxide (DMSO). For Western blotting (0.5, 1, 2, and 3 µM), soft agar (0.5 µM), and in vivo assays (10 mg/kg), cells were treated with the mentioned concentration of drug and collected at appropriate time points for protein isolation or colony counting.

### MTT assay

Cells (2 × 10^3^ cells per well) were seeded in a 96-well plate, incubated with pyrvinium pamoate (six replicates per concentration) for 72 h, and subsequently incubated with 0.5 mg/ml of MTT (cat. no. TC191; HiMedia) for 3 h. MTT assay data were acquired at 562 nm using a microplate reader. The percentage of cell viability was calculated in comparison with the vehicle control. All assays were performed in triplicate.

### In vivo study

All in vivo experiments were performed as approved by the Institutional Animal Ethics Committee (IAEC), TMC-ACTREC. Stable clones of NIH/3T3 overexpressing *UBE3C-LRP5* fusion or empty vector were trypsinized and suspended in 40 μL sterile 1X PBS. Cells were injected subcutaneously (three million cells/mouse) into 6–8-week-old male NOD-SCID (non-obese diabetic/severe combined immunodeficiency) mice (*n* = 6/group). Caliper measurements were performed every three days to monitor the tumor volumes. For both the inhibitor and survival studies, we performed two separate sets of experiments, with each set comprising a total of 12 mice. In each of these experiments, we injected three million NIH/3T3 cells overexpressing the *UBE3C-LRP5* fusion clones subcutaneously into 6–8-week-old NOD-SCID mice (*n* = 6/group) and were grouped to receive pyrvinium pamoate (10 mg/kg) or vehicle. Treatment was given to mice orally using oral gavage at intervals of 48 h for 9 days after the tumor volume reached 100 mm^3^ (24 days after cell injection). The treatment response was monitored every three days by measuring the tumor volume using a Vernier caliper.

### Survival analysis

Survival analysis was performed using the Kaplan–Meier plotter online tool^71^ in the in-house and TCGA-HNSC samples. Patient clinical data were imported into the Kaplan–Meier plotter server using the custom data option. *UBE3C-LRP5* fusion status (with or without fusion) was assigned to the samples assessed in the survival analysis.

### Statistics and reproducibility

Statistical analysis was performed using GraphPad Prism version 8 (GraphPad Software, La Jolla, CA, USA). Student’s unpaired *t*-test was used to determine the statistical significance between different groups, and the *p* values calculated are denoted as ns (not significant); *p < 0.05; **p < 0.01; ***p < 0.001; ****p < 0.0001. The reproducibility of the experimental findings was confirmed by performing *n* = 3 independent replicates. The results of all the biological replicates were consistent.

### Reporting summary

Further information on research design is available in the Nature Research Reporting Summary linked to this article.

### Supplementary information

REPORTING SUMMARY

Supplementary Information

## Data Availability

The raw sequencing data generated and analysed during the current study are available from the ArrayExpress repository (http://www.ebi.ac.uk/arrayexpress/), hosted by the European Bioinformatics Institute (EBI), under the following accession numbers: E-MTAB-3958: transcriptome sequencing data of cell lines, E-MTAB-4654: transcriptome sequencing data of tissue samples, E-MTAB-12534: whole genome sequencing data of NT-8e cell line (AD2880).
