# Supplementary material for: Recurrent UBE3C-LRP5 translocations in head and neck cancer with therapeutic implications
Source: NPJ Precis Oncol. 2024 Mar 4;8:63. doi: 10.1038/s41698-024-00555-4 (PMC10912599; doi:10.1038/s41698-024-00555-4)
Supplement: Supplementary file 1 — REPORTING SUMMARY [file 41698_2024_555_MOESM1_ESM.pdf]

nature portfolio

Corresponding author(s): Amit Dutt

Last updated by author(s): Feb 11, 2024

Reporting Summary

Nature Portfolio wishes to improve the reproducibility of the work that we publish. This form provides structure for consistency and transparency in reporting. For further information on Nature Portfolio policies, see our Editorial Policies and the Editorial Policy Checklist.

Statistics

For all statistical analyses, confirm that the following items are present in the figure legend, table legend, main text, or Methods section.

n/a	Confirmed	
☐	☒	The exact sample size (n) for each experimental group/condition, given as a discrete number and unit of measurement
☐	☒	A statement on whether measurements were taken from distinct samples or whether the same sample was measured repeatedly
☐	☒	The statistical test(s) used AND whether they are one- or two-sided<br>*Only common tests should be described solely by name; describe more complex techniques in the Methods section.*
☐	☒	A description of all covariates tested
☒	☐	A description of any assumptions or corrections, such as tests of normality and adjustment for multiple comparisons
☐	☒	A full description of the statistical parameters including central tendency (e.g. means) or other basic estimates (e.g. regression coefficient) AND variation (e.g. standard deviation) or associated estimates of uncertainty (e.g. confidence intervals)
☐	☒	For null hypothesis testing, the test statistic (e.g. F, t, r) with confidence intervals, effect sizes, degrees of freedom and P value noted<br>*Give P values as exact values whenever suitable.*
☒	☐	For Bayesian analysis, information on the choice of priors and Markov chain Monte Carlo settings
☒	☐	For hierarchical and complex designs, identification of the appropriate level for tests and full reporting of outcomes
☒	☐	Estimates of effect sizes (e.g. Cohen's d, Pearson's r), indicating how they were calculated

Our web collection on statistics for biologists contains articles on many of the points above.

Software and code

Policy information about availability of computer code

Data collection	No software was used for data collection. For survival analysis, clinical data of the in-house and TCGA-HNSC data was directly imported into the Kaplan-Meier plotter server using the the custom data option.
Data analysis	Transcriptome sequencing data fusion analysis:<br>ChimeraScan tool was used to identify fusion transcripts using default parameters in tumor, normal, and cell lines. For fusion mapping, paired-end raw read sequences were mapped to human reference genome sequences (hg19). Using scripts developed in-house in Python, we filtered fusion pairs without spanning read support, transcript allele fraction (TAF) < 0.01, for both the partner, pseudogenes, and homologous sequences spanning reads. Tumor-specific fusions were further processed for annotation using Oncofuse, and the frame of fusion was determined. The upstream and downstream sequences supporting fusion were retrieved, and primers were designed using Primer-BLAST.<br><br>TCGA-HNSC data fusion analysis:<br>The RNA-seq tier 1 data (aligned BAM files) of the TCGA-HNSC project (n=502) were downloaded from the National Cancer Institute Cancer Genome Commons Portal (http://portal.gdc.cancer.gov). The BAM files were converted to raw fastq files using the SamToFastq utility of the Picard toolkit (https://broadinstitute.github.io/picard/). Primary alignment of the transcriptome data was performed against GRCh38 (GRCh.p12 GENCODE v30) using the two-pass mode of the STAR aligner (v2.7.6a). Discordant and split reads mapping to LRP5/ UBE3C genomic coordinates were extracted and annotated using in-house custom scripts. The reads supporting the breakpoints were manually superimposed to derive contigs.<br><br>Whole genome sequencing and SvABA analysis:<br>Whole genome sequencing data of the NT-8e cell line was aligned to the human reference genome (GRCh38.p12 GENCODE v30) using BWA-MEM (v0.7.17). The BAM files were further analyzed using SvABA (v1.1.3) to identify translocation breakpoints. The translocation breakpoints

were annotated using custom scripts, using the reference GENCODE GTF (v30). The translocation breakpoints identified in the SvABA analysis were additionally confirmed using MANTA (v1.6.0).

Survival analysis:
Survival analysis was performed using the Kaplan-Meier plotter online tool in the in-house and TCGA-HNSC samples. Patient clinical data were imported into the Kaplan-Meier plotter server using custom data option. UBE3C-LRP5 fusion status (with or without fusion) was assigned to the samples assessed in the survival analysis.

Statistical analysis:
Statistical analysis was performed using GraphPad Prism version 8 (GraphPad Software, La Jolla, CA, USA). Student's unpaired t-test was used to determine the statistical significance between different groups, and the p-values calculated are denoted as ns (not significant); *, p<0.05; **, p<0.01; ***, p<0.001; ****, p<0.0001.

For manuscripts utilizing custom algorithms or software that are central to the research but not yet described in published literature, software must be made available to editors and reviewers. We strongly encourage code deposition in a community repository (e.g. GitHub). See the Nature Portfolio guidelines for submitting code & software for further information.

Data

Policy information about availability of data

All manuscripts must include a data availability statement. This statement should provide the following information, where applicable:

- Accession codes, unique identifiers, or web links for publicly available datasets
- A description of any restrictions on data availability
- For clinical datasets or third party data, please ensure that the statement adheres to our policy

The raw sequencing data generated and analysed during the current study are available from the ArrayExpress repository (http://www.ebi.ac.uk/arrayexpress/), hosted by the European Bioinformatics Institute (EBI), under the following accession numbers: E-MTAB-3958: transcriptome sequencing data of cell lines, E-MTAB-4654: transcriptome sequencing data of tissue samples, E-MTAB-12534: whole genome sequencing data of NT-8e cell line (AD2880).

Research involving human participants, their data, or biological material

Policy information about studies with human participants or human data. See also policy information about sex, gender (identity/presentation), and sexual orientation and race, ethnicity and racism.

Reporting on sex and gender	Gender (self reported) was considered in the study design. The findings of the study applies to both the genders. The consent was obtained from all the patients for using the gender information data. We have not performed any gender-based analyses in this study.
Reporting on race, ethnicity, or other socially relevant groupings	Our study does not include any information on race, ethnicity, or other socially relevant groupings.
Population characteristics	Please refer to Table 1 of the manuscript.
Recruitment	A total of 151 fresh frozen head and neck cancer samples with adequate tumor content and quality were collected from the tumor tissue repository of Tata Memorial Hospital (TMH-TTR) and the Advanced Centre for Treatment, Research and Education in Cancer (ACTREC-TTR).
Ethics oversight	Samples were collected with the approval of The Institutional Review Board (IRB) and the Ethics Committee (EC) of Tata Memorial Centre-ACTREC.

Note that full information on the approval of the study protocol must also be provided in the manuscript.

Field-specific reporting

Please select the one below that is the best fit for your research. If you are not sure, read the appropriate sections before making your selection.

☒ Life sciences ☐ Behavioural & social sciences ☐ Ecological, evolutionary & environmental sciences

For a reference copy of the document with all sections, see nature.com/documents/nr-reporting-summary-flat.pdf

Life sciences study design

All studies must disclose on these points even when the disclosure is negative.

Sample size	The sample size used in the study was not predetermined. 151 fresh frozen tongue tumor samples were collected from the tumor tissue repository of Tata Memorial Hospital and TMC-ACTREC.
Data exclusions	No data was excluded from the analyses.

Replication	Reproducibility of the experimental findings were confirmed by performing at least 3 biological replicates of each experiment. The findings of all the biological replicates were consistent.
Randomization	Randomization was not required for our study as we screened for the expression of LRP5-UBE3C and UBE3C-LRP5 fusion transcripts in head and neck cancer primary tumor samples. For in vivo studies, mice were randomized before subcutaneous injection of the cells and treatment with pyrvinium pamoate.
Blinding	Blinding was not relevant to our study as we do not correlate the presence/absence of LRP5-UBE3C and UBE3C-LRP5 fusion transcripts with the clinico-pathological features of the head and neck cancer patients.

Reporting for specific materials, systems and methods

We require information from authors about some types of materials, experimental systems and methods used in many studies. Here, indicate whether each material, system or method listed is relevant to your study. If you are not sure if a list item applies to your research, read the appropriate section before selecting a response.

Materials & experimental systems

n/a	Involved in the study	
☐	☒	Antibodies
☐	☒	Eukaryotic cell lines
☒	☐	Palaeontology and archaeology
☐	☒	Animals and other organisms
☒	☐	Clinical data
☒	☐	Dual use research of concern
☒	☐	Plants

Methods

n/a	Involved in the study	
☒	☐	ChIP-seq
☒	☐	Flow cytometry
☒	☐	MRI-based neuroimaging

Antibodies

Antibodies used	LRP5 (cat. no. sc-390267, Santa Cruz Biotechnology)<br>DYKDDDDK tag (cat. no. 8146, Cell Signaling Technology)<br>β-Tubulin (cat. no. 2128, Cell Signaling Technology)<br>β-catenin (cat. no. ab32572, Abcam)<br>Lamin B1 (cat. no. sc-374015, Santa Cruz Biotechnology)<br>GAPDH (cat. no. sc-32233, Santa Cruz Biotechnology)<br>Vinculin (cat no. 4650, Cell Signaling Technology)<br>β-actin (cat. no. sc-47778, Santa Cruz Biotechnology)<br>Goat anti-rabbit IgG-HRP secondary antibody (sc-2004, Santa Cruz Biotechnology)<br>Goat anti-mouse IgG-HRP secondary antibody (sc-2005, Santa Cruz Biotechnology)
Validation	All the antibodies were commercially purchased and were previously validated by the vendors and described in literature. In detail:<br>LRP5 (cat. no. sc-390267, Santa Cruz Biotechnology)- LRP5 Antibody (B-9) is a mouse monoclonal IgG1 κ LRP5 antibody. Applications: IP, IF and ELISA<br>DYKDDDDK tag (cat. no. 8146, Cell Signaling Technology)- DYKDDDDK Tag (9A3) Mouse mAb detects exogenously expressed DYKDDDDK proteins in cells. Applications: WB, IP, IHC, IF and Flow Cytometry<br>β-Tubulin (cat. no. 2128, Cell Signaling Technology)- β-Tubulin (9F3) Rabbit mAb detects endogenous levels of total β-tubulin protein, and does not cross-react with recombinant α-tubulin. Applications: WB, IHC, IF and Flow Cytometry<br>β-catenin (cat. no. ab32572, Abcam)- Rabbit monoclonal [E247] to beta Catenin - ChIP Grade. Applications: WB, IHC-P, ICC/IF, IP and ChIP<br>Lamin B1 (cat. no. sc-374015, Santa Cruz Biotechnology)- Anti-Lamin B1 Antibody (B-10) is recommended for detection of Lamin B1 of mouse, rat and human origin. Applications: WB, IP, IF and ELISA<br>GAPDH (cat. no. sc-32233, Santa Cruz Biotechnology)- GAPDH (6C5) is a mouse monoclonal antibody raised against GAPDH purified from muscle of rabbit origin. Applications: WB, IP, IF<br>Vinculin (Cat No. 4650S, Cell Signaling Technology)-Vinculin Antibody detects endogenous levels of total vinculin protein. This antibody also reacts with metavinculin, a 145 kDa splice variant of vinculin. Applications: WB<br>β-actin (cat. no. sc-47778, Santa Cruz Biotechnology)- Anti-β-Actin Antibody (C4) is a mouse monoclonal IgG1 κ β-Actin antibody. Applications: WB, IP<br>Goat anti-rabbit IgG-HRP secondary antibody (sc-2004, Santa Cruz Biotechnology)- goat anti-rabbit IgG-HRP is an affinity purified secondary antibody raised in goat against whole rabbit IgG and conjugated to HRP (horseradish peroxidase). Applications: goat anti-rabbit IgG-HRP is recommended for detection of rabbit IgG by Western Blotting.<br>Goat anti-mouse IgG-HRP secondary antibody (sc-2005, Santa Cruz Biotechnology)- goat anti-mouse IgG-HRP is an affinity purified secondary antibody raised in goat against mouse IgG and conjugated to HRP (horseradish peroxidase). Applications- goat anti-mouse IgG-HRP is recommended for detection of mouse IgG by Western Blotting.

Eukaryotic cell lines

Policy information about cell lines and Sex and Gender in Research

Cell line source(s)	AW13516, AW8507 (Tatake et al., J. Cancer Res. Clin. Oncol., 1990), NT-8e (Mulherkar, R. et al., Cancer letters, 1997), and OT-9 (Mulherkar, R. et al., Cancer letters, 1997; Dhawan, V. V. et al., Cellular oncology, 2014) head and neck cancer cell lines were established within Tata Memorial Centre and were acquired from Tata Memorial Hospital (Mumbai, India). NIH/3T3 (CRL-1658) cell line was procured from ATCC. Stable LRP5-UBE3C and UBE3C-LRP5 overexpression clones were generated in-house from parental cell lines.
Authentication	The cell lines were authenticated by DNA short tandem repeat (STR) profiling using Promega Geneprint 10 system in conjugation with GeneMarker HID software tool.
Mycoplasma contamination	Cells were tested for mycoplasma and found to be negative, but as a standard lab protocol, we treated the cells using EZKill Mycoplasma Removal Reagent (cat. no. CCK006-1; HiMedia) every six months.
Commonly misidentified lines (See ICLAC register)	No commonly misidentified cell lines were used in the study.

Animals and other research organisms

Policy information about studies involving animals; ARRIVE guidelines recommended for reporting animal research, and Sex and Gender in Research

Laboratory animals	6-8 week-old male NOD-SCID (non-obese diabetic/severe combined immunodeficiency) mice used in the study were obtained from TMC-ACTREC animal facility.
Wild animals	Study did not involve wild animals.
Reporting on sex	There was no sex or gender specific analyses performed in the animal studies.
Field-collected samples	Study did not involve samples collected from the field.
Ethics oversight	All in vivo experiments were performed as approved by Institutional Animal Ethics Committee (IAEC), TMC-ACTREC.

Note that full information on the approval of the study protocol must also be provided in the manuscript.

Plants

Seed stocks	n/a
Novel plant genotypes	n/a
Authentication	n/a
