# Supplementary material for: Recurrent UBE3C-LRP5 translocations in head and neck cancer with therapeutic implications
Source: NPJ Precis Oncol. 2024 Mar 4;8:63. doi: 10.1038/s41698-024-00555-4 (PMC10912599; doi:10.1038/s41698-024-00555-4)
Supplement: Supplementary file 2 — Supplementary Information [file 41698_2024_555_MOESM2_ESM.pdf]

Supplementary Information for

Recurrent *UBE3C-LRP5* Translocations in Head and Neck Cancer with Therapeutic Implications

Bhasker Dharavath[1,2], Ashwin Butle[1], Akshita Chaudhary[1], Ankita Pal[1], Sanket Desai[1], Aniket Chowdhury[1,2], Rahul Thorat[3], Pawan Upadhyay[1], Sudhir Nair[2,4], Amit Dutt[1,2,5*]

1. Integrated Cancer Genomics Laboratory, Advanced Centre for Treatment, Research, and Education in Cancer, Kharghar, Navi Mumbai, Maharashtra, 410210, India
2. Homi Bhabha National Institute, Training School Complex, Anushakti Nagar, Mumbai, Maharashtra, 400094, India
3. Laboratory Animal Facility, Advanced Centre for Treatment, Research and Education in Cancer, Kharghar, Navi Mumbai, Maharashtra, 410210, India
4. Division of Head and Neck Oncology, Department of Surgical Oncology, Tata Memorial Hospital, Tata Memorial Centre, Parel, Mumbai, 400012, India
5. Department of Genetics, University of Delhi South Campus, New Delhi, 110021, India.

*Corresponding author:
Dr. Amit Dutt (adutt@actrec.gov.in)

Supplementary Figure S1 to S28 and Table S1 to S9

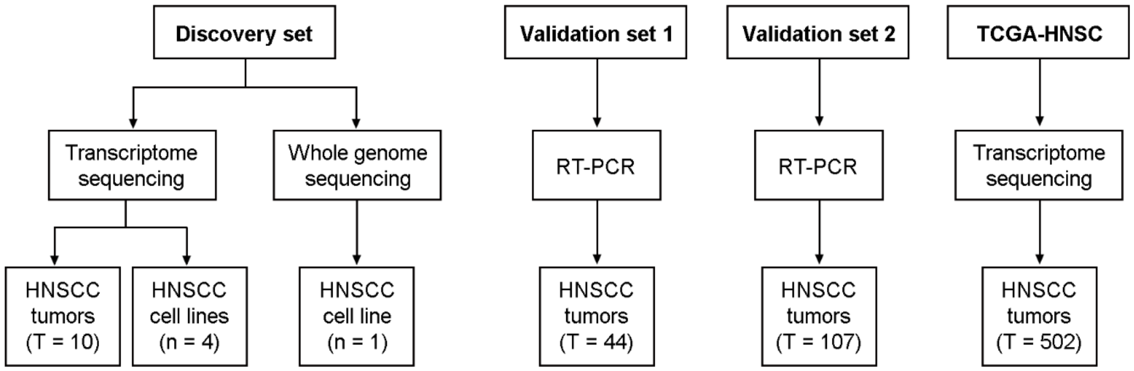

Supplementary Figure S1: Schematic representation depicting the overview of samples in the discovery and validation sets.

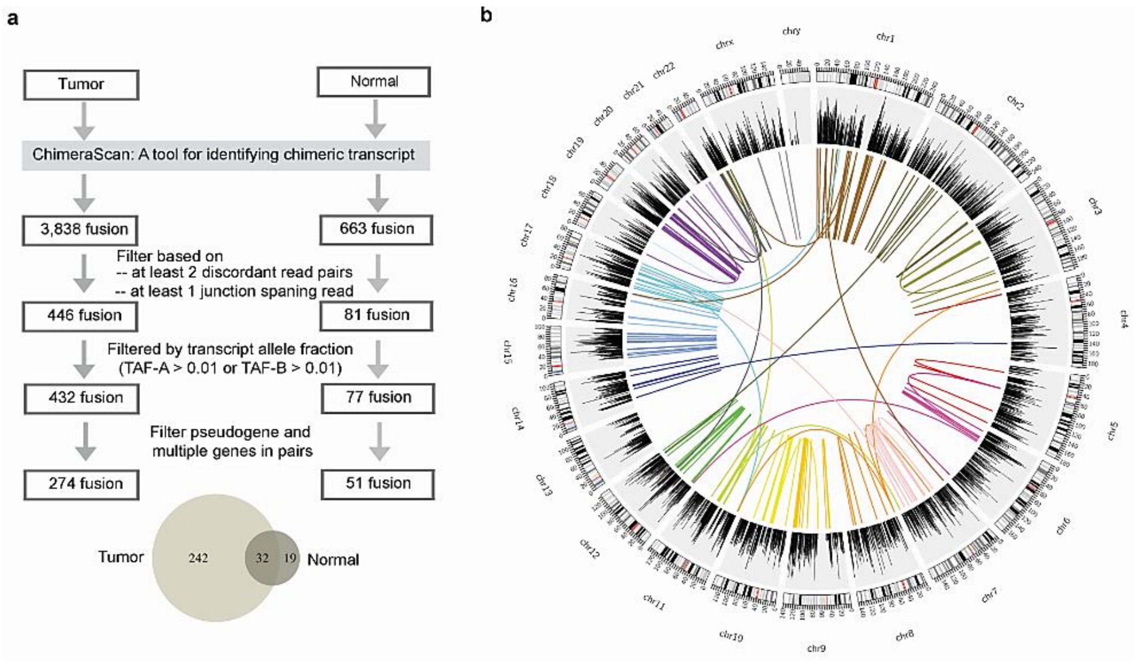

Supplementary Figure S2: Landscape of transcript fusions identified in head and neck cancer. (a) Flow chart representation of the analysis pipeline and filters used in the analysis, such as spanning reads, transcript allele fraction (TAF), and pseudogene homology to identify and prioritize the putative high-confidence transcript fusions in the study. (b) Circos plot representation of candidate fusion transcripts identified in head and neck tumors. From outside to inside: karyotype, Gene expression (TPM), and fusion transcripts. Black line tracks indicate the gene expression, and fusion transcripts are colored by their chromosome of origin.

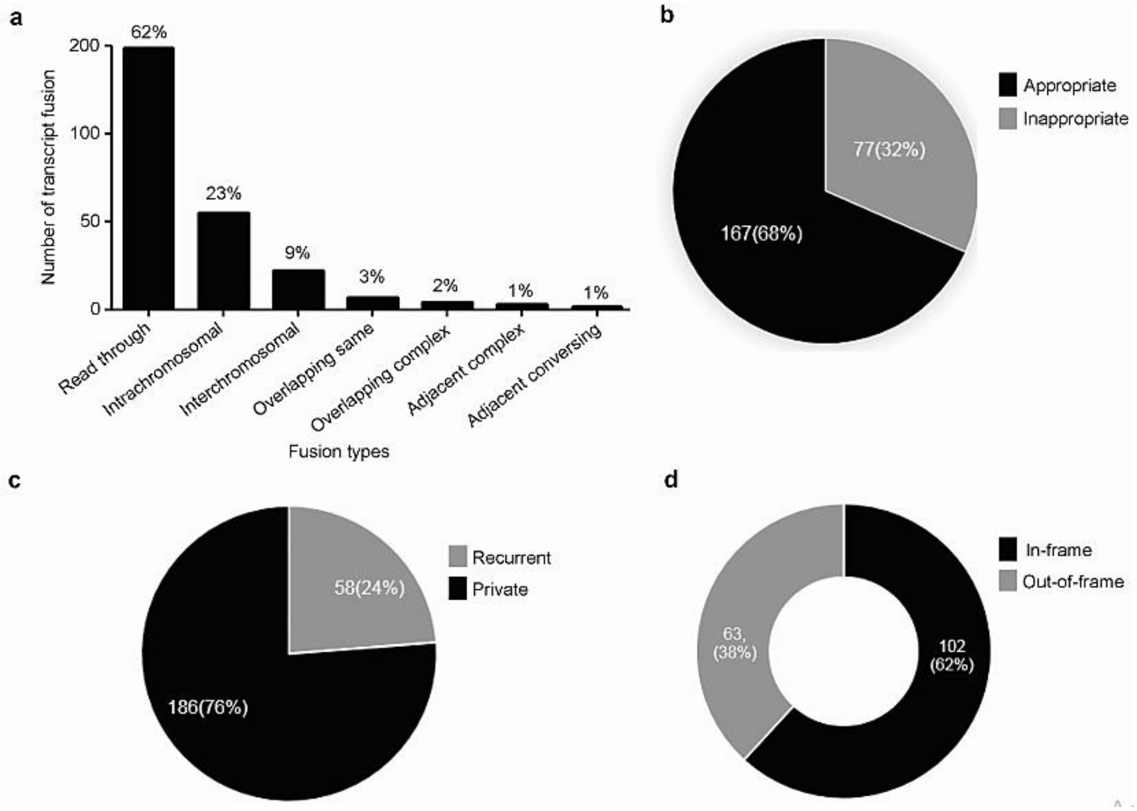

Supplementary Figure S3: Characteristic features of transcript fusions. (a) The bar graph representation of different types of fusion transcripts identified in head and neck tumor samples. The percentage frequency of each type of fusion is denoted at the top of each bar. (b) Pie-chart representation of number and percent frequency of appropriate (donor (5')-acceptor (3') relationships) and inappropriate (donor-donor or acceptor –acceptor) transcript fusions. (c) Pie-chart representation of number and percent frequency of private and recurrent fusion transcript. (d) Doughnut plot representation of number and percent frequency of reading frame of fusion transcripts (in-frame –continuous reading frame for translation and out-of-frame – reading frame with stop codon or truncation).

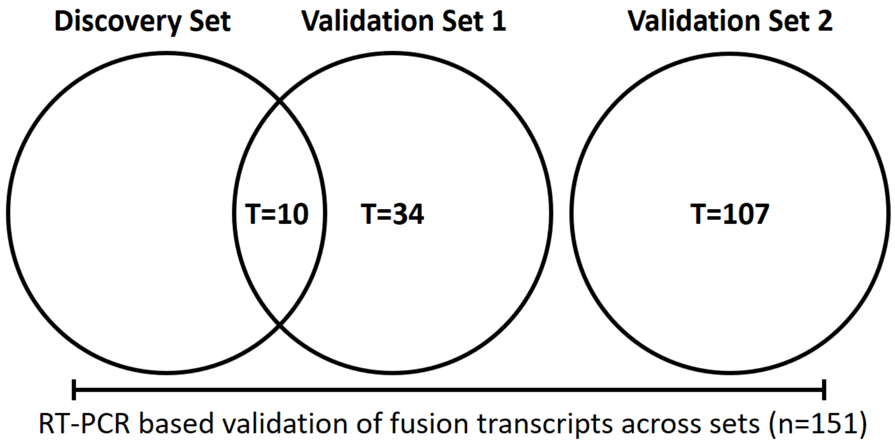

Supplementary Figure S4: RT-PCR based validation of *LRP5-UBE3C*, and *UBE3C-LRP5* fusion transcript variants in patient samples. Venn diagram indicating the number of samples (T=primary tumors) in the discovery and validation cohorts. The intersection in the Venn diagram circles represent the overlapping samples in the two cohorts.

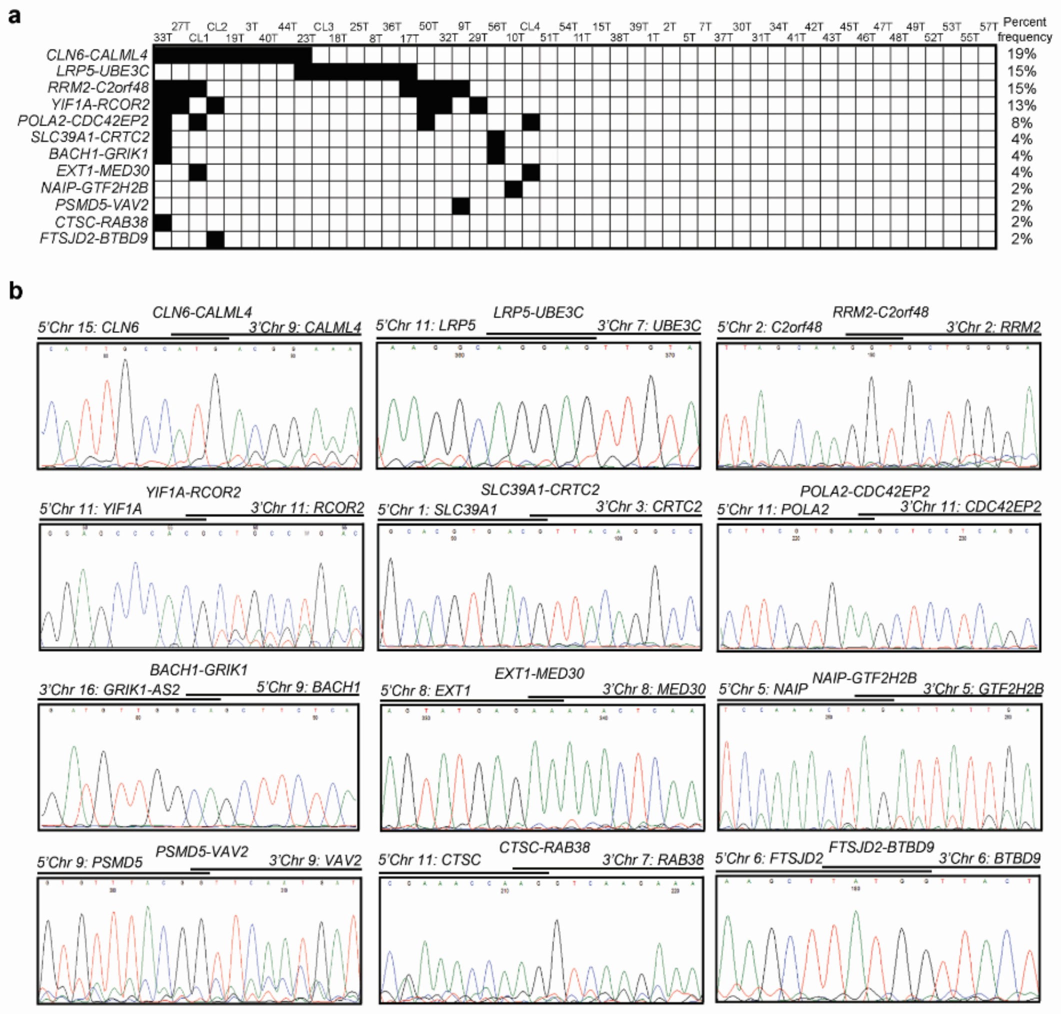

Supplementary Figure S5: Validation of fusion transcripts across 44 paired head and neck tumor samples and cell lines. (a) Heatmap representation of twelve validated fusion transcripts by RT-PCR followed by Sanger sequencing confirmation in a cohort of 44 paired HNSCC tumors and 4 cell lines. The black-filled boxes denote samples positive for fusion transcript by Sanger sequencing and white for no event. The percentage frequency is shown for each fusion transcript in the cohort. The sample name CL1, CL2, CL3, CL4 denotes AW13516, OT9, NT-8e and AW8507 cell lines, respectively. (b) Representative Sanger sequencing chromatogram of twelve validated fusions using RT-PCR followed by Sanger sequencing. For each fusion transcript, chromosome number, gene name, and the direction of the gene are shown as 5’ and 3’.

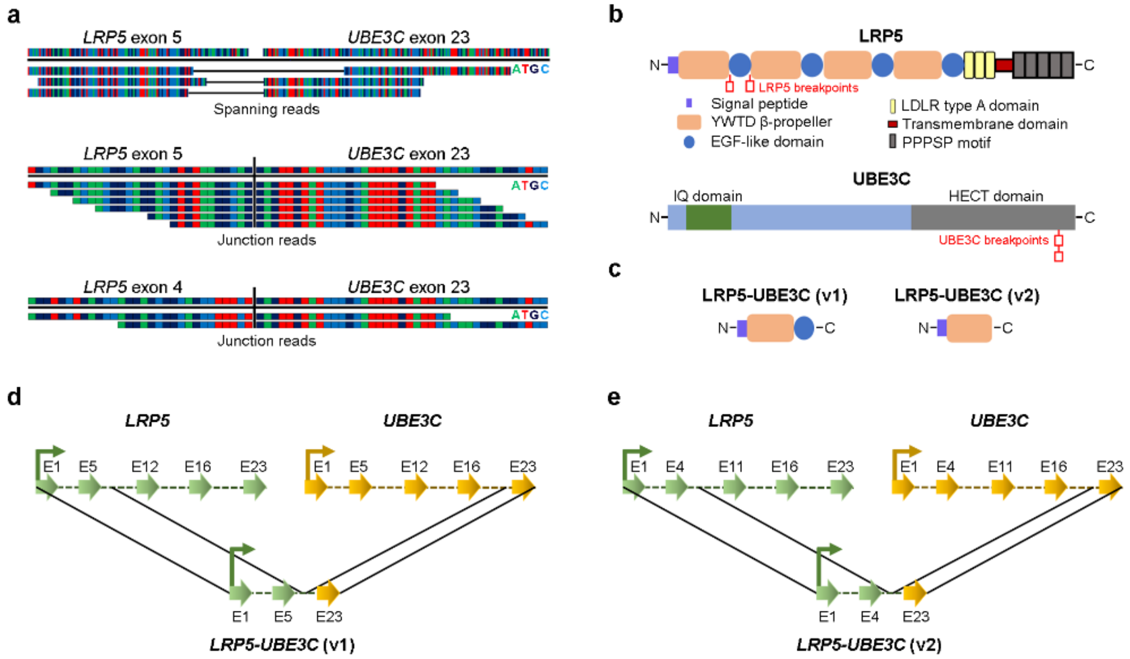

Supplementary Figure S6: Identification of *LRP5-UBE3C* fusion variants in head and neck cancer. (a) Spanning and junction read supporting the *LRP5-UBE3C* fusion variants, as detected from the transcriptome sequencing data of the NT-8e cell line. (b) Schematic representation of LRP5 and UBE3C protein domains and the breakpoints identified. Red boxes indicate breakpoints identified in the NT-8e cell line, and in-house HNSCC patient samples. (c) Schematic representation of the predicted protein domains of the LRP5-UBE3C fusion variants (v1, v2). (d, e) Schematic representation of LRP5-UBE3C fusion variants v1 (d) and v2 (e), depicting the directionality of the fusion transcripts. Arrows represent the exons (E1-E23) in both the genes.

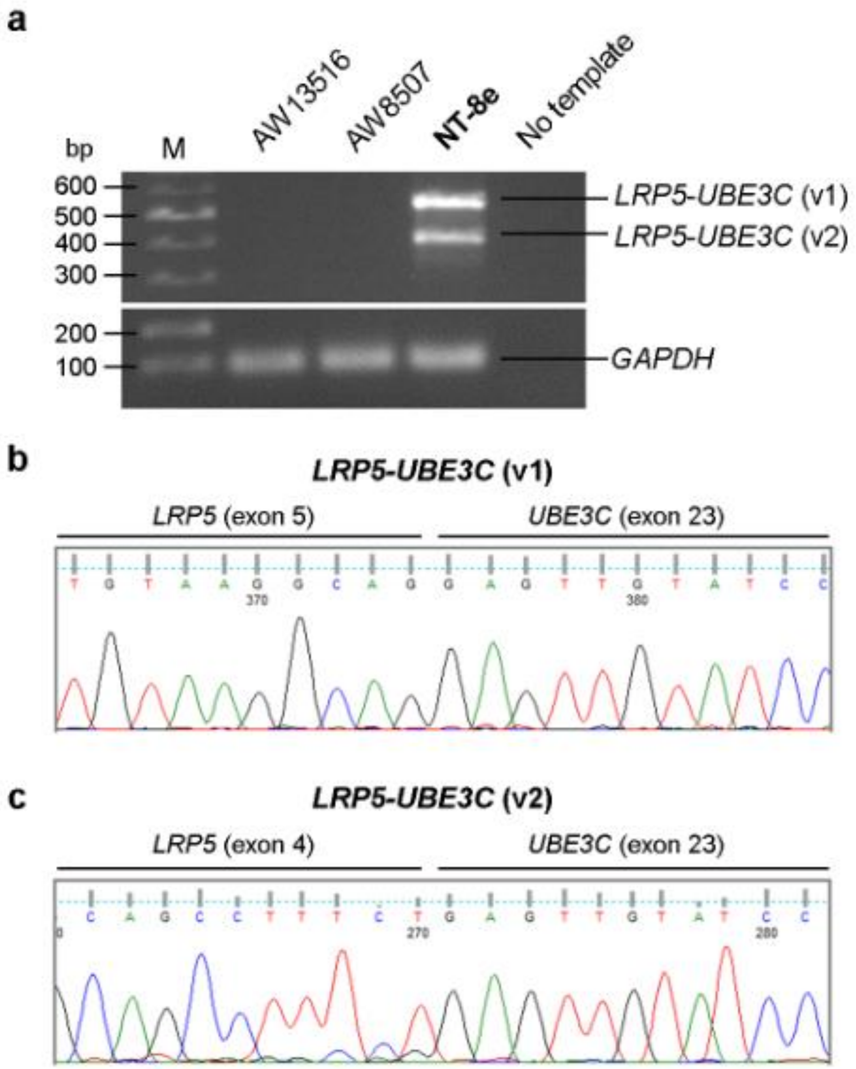

Supplementary Figure S7: Validation of *LRP5-UBE3C* fusion transcript variants in NT-8e cell line using RT-PCR and Sanger sequencing. (a) RT-PCR data of *LRP5-UBE3C* (v1) and *LRP5-UBE3C* (v2) fusion transcripts in HNSCC cell lines. *GAPDH* transcripts serve as an internal reference control. The gels are from the same experiment and were processed in parallel. (b, c) Sanger sequencing traces of *LRP5-UBE3C* fusion transcript variants, v1 (b) and v2 (c), amplified from cDNA of NT-8e cell line.

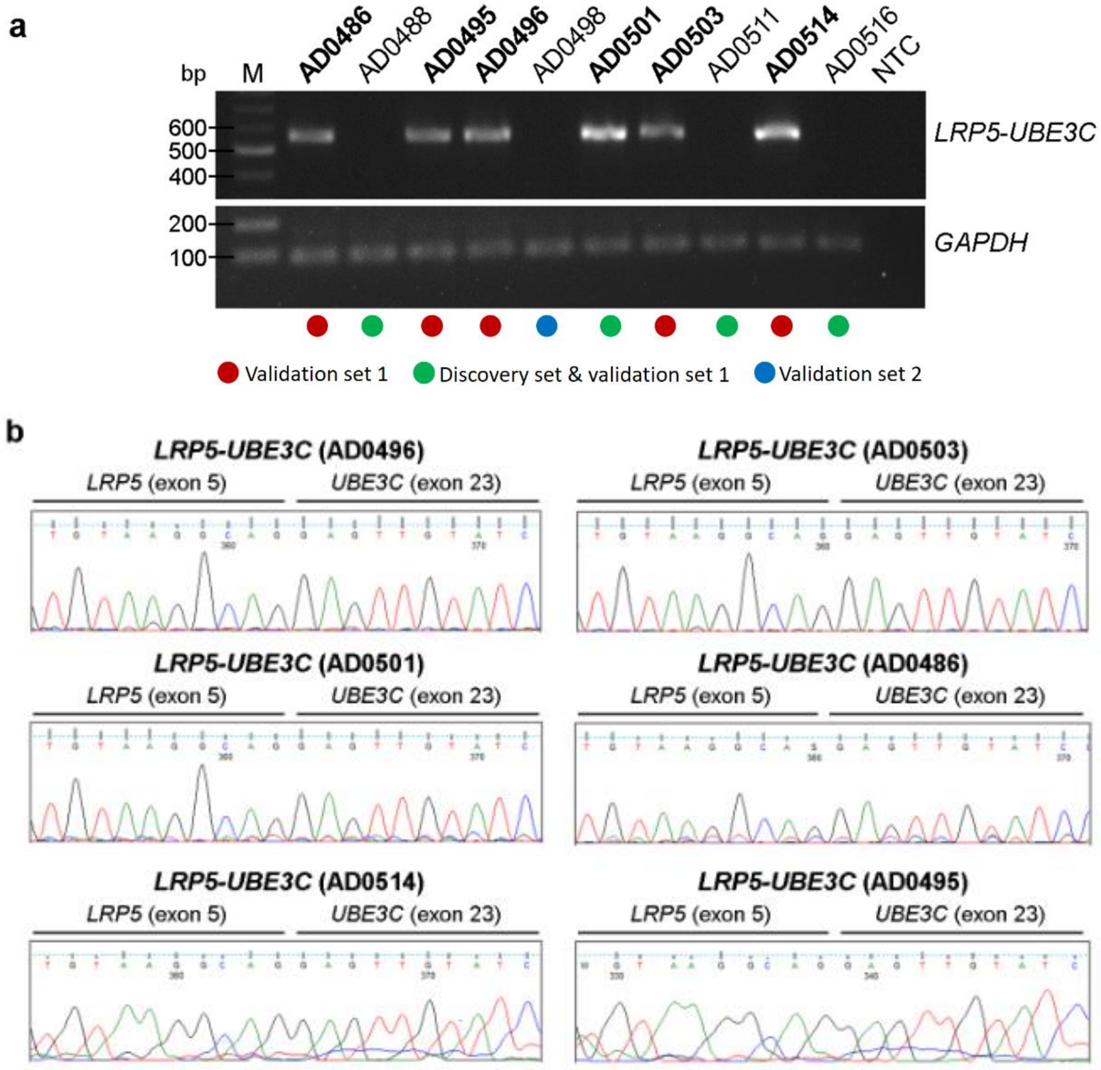

Supplementary Figure S8: Electropherogram showing Sanger sequencing of the *LRP5-UBE3C* fusion transcript in HNSCC tumor samples. (a) RT-PCR data of *LRP5-UBE3C* (v1) fusion transcript and *GAPDH* in HNSCC primary tumor samples. The top gel image is a representation of the tumor samples positive for the fusion (n = 6) along with four fusion-negative controls, and one no template control (NTC). Red color dot at the bottom of the gel indicates the samples from validation set 1, green color dot indicates the common samples from discovery and validation set 1, and blue color dot indicate samples from validation set 2. The gels are from the same experiment and were processed in parallel. (b) Sanger sequencing traces of *LRP5-UBE3C* fusion transcript (v1) amplified from cDNA of six primary tumor samples. Sample IDs are mentioned on top of the gel image and each electropherogram.

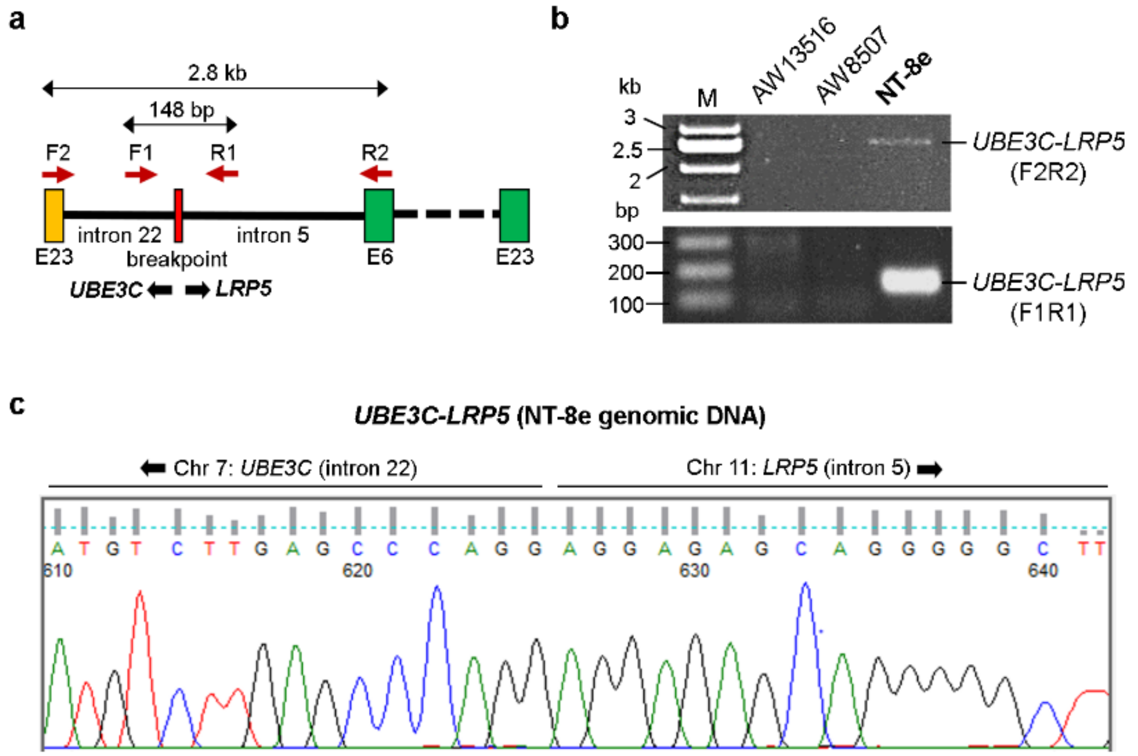

Supplementary Figure S9: Validation of *UBE3C-LRP5* fusion breakpoint at the genomic DNA level in the NT-8e cell line. (a) Schematic representation of primer locations in the *UBE3C-LRP5* gene fusion. The direction of *UBE3C* and *LRP5* in the fusion is represented with the black arrows. The yellow color box (E23) represents the exon 23 of inverted *UBE3C* and the green color boxes (E6, E23) along with the dotted lines represent the region of exon 6-23 of *LRP5*. Primer locations (F1, F2, R1, and R2) are indicated at the top of the introns/exons along with the nucleotide distance. (b) PCRs of *UBE3C-LRP5* gene fusion with the combination of primers (F1R1 and F2R2) in the genomic DNA of HNSCC cell lines. The gels are from the same experiment and were processed in parallel. (c) Sanger sequencing traces of *UBE3C-LRP5* fusion gene amplified from genomic DNA of NT-8e cell line.

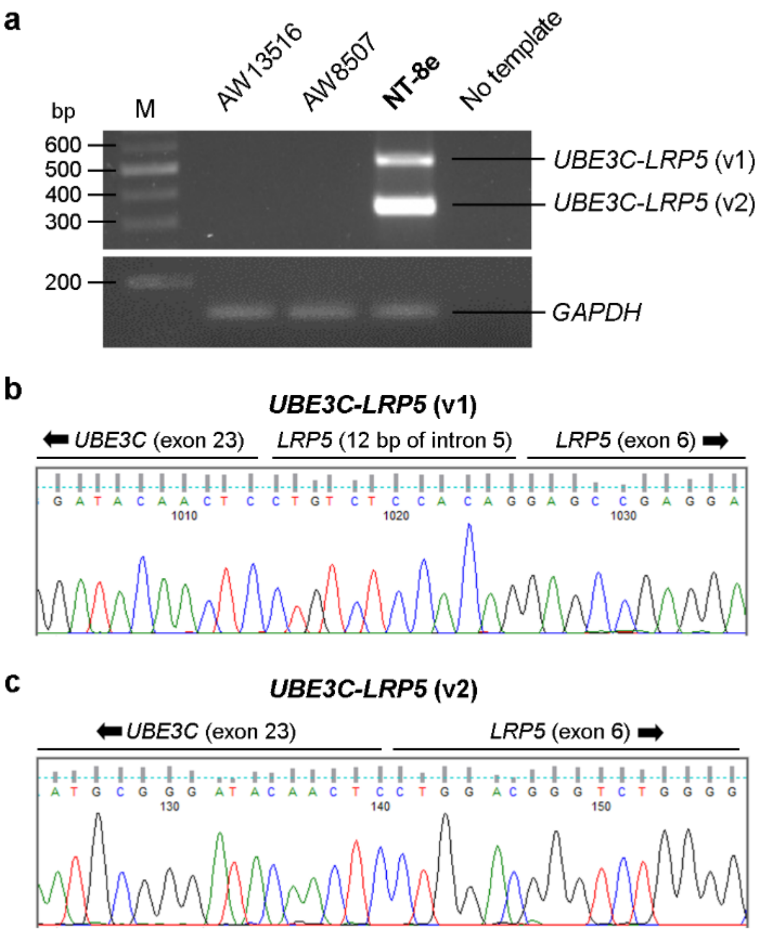

Supplementary Figure S10: Identification and validation of *UBE3C-LRP5* fusion transcript variants in NT-8e cell line. (a) RT-PCR of *UBE3C-LRP5* (v1), *UBE3C-LRP5* (v2) fusion transcripts, and *GAPDH* in HNSCC cell lines. The gels are from the same experiment and were processed in parallel. (b, c) Sanger sequencing traces of *UBE3C-LRP5* fusion transcript variants, v1 (b) and v2 (c), amplified from the cDNA of the NT-8e cell line.

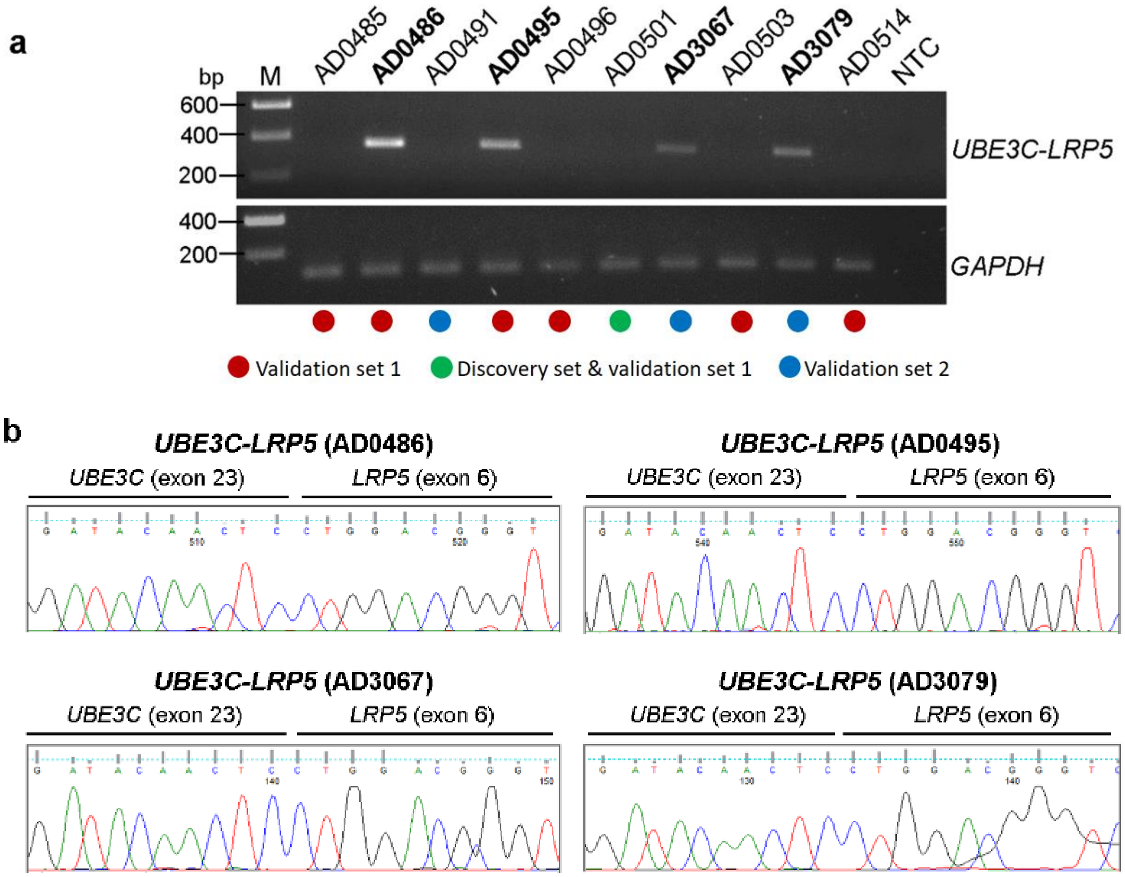

Supplementary Figure S11: Identification of *UBE3C-LRP5* fusion transcript in HNSCC patient samples. (a) RT-PCR of *UBE3C-LRP5* (v2) fusion transcript and *GAPDH* in HNSCC tumor samples. The top gel image is a representation of the tumor samples positive for the fusion (n = 4) along with six fusion-negative controls, and one no template control (NTC). Red color dot at the bottom of the gel indicates the samples from validation set 1, blue color dot indicate samples from validation set 2, and green color dot indicates the common sample from discovery and validation set 1. The gels are from the same experiment and were processed in parallel. (b) Sanger sequencing traces of *UBE3C-LRP5* (v2) fusion transcript amplified from cDNA of primary tumor samples. Sample IDs are mentioned on top of the gel image and each electropherogram. NTC represents no template control.

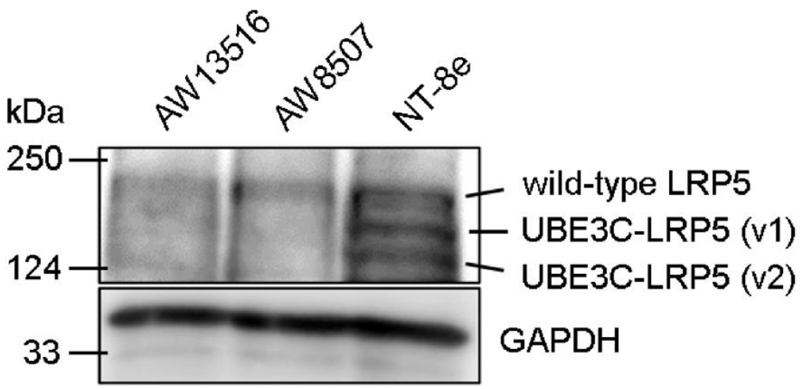

Supplementary Figure S12: Validation of UBE3C-LRP5 fusion protein in the NT-8e cell line. Immunoblot of wild-type LRP5, UBE3C-LRP5 fusion variants (v1, and v2), and GAPDH in AW13516, AW8507, and NT-8e cell lines. The blots are from the same experiment and were processed in parallel.

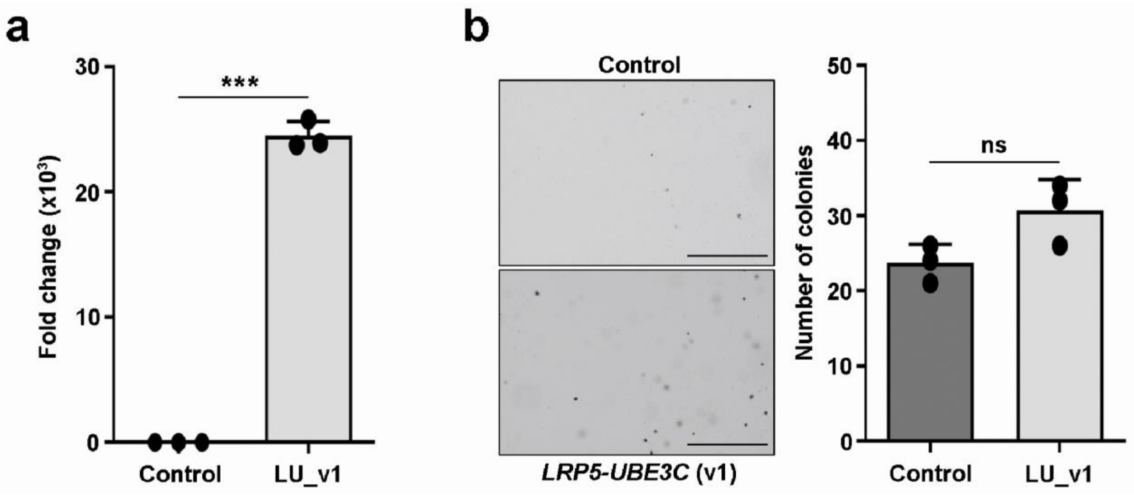

Supplementary Figure S13: *LRP5-UBE3C* fusion transcript is not transforming *in vitro*. (a) qRT-PCR of *LRP5-UBE3C* fusion transcript in NIH/3T3 cells stably overexpressing empty vector or *LRP5-UBE3C* (LU) fusion variant (LU_v1). *Gapdh* was used for the normalization of gene expression. (b) Soft agar anchorage-independent growth assay of NIH/3T3 cells stably overexpressing the empty vector or fusion transcript variant. The bar plot indicates the number of colonies in fusion overexpression clones and vector control cells. Data are shown as means ± SD. *p*-values are from Student's unpaired t-test and denoted as *ns* (not significant); ***, $p < 0.001$. The data shown are representative of n = 3 independent experiments. Scale bar = 400 µM.

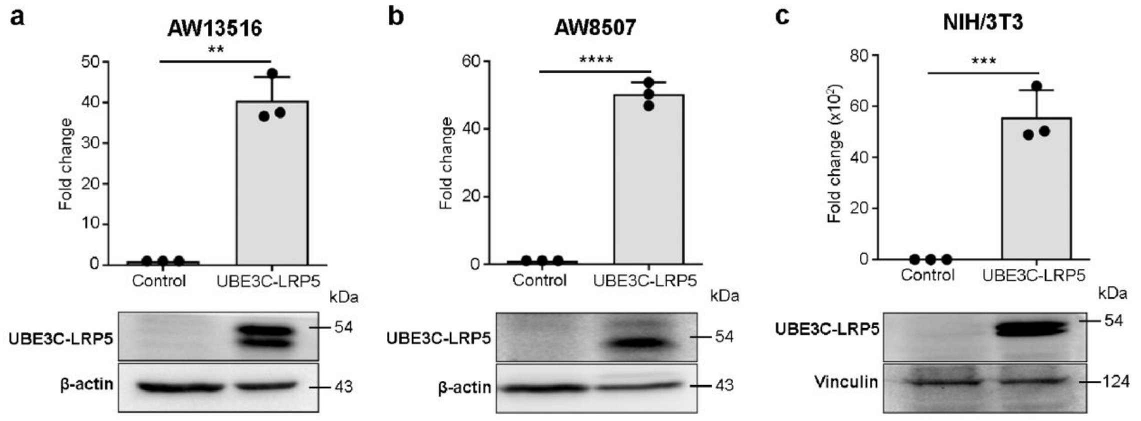

Supplementary Figure S14: Overexpression of *UBE3C-LRP5* fusion variant (v7) in cell lines. (a-c) qRT-PCR and immunoblot of UBE3C-LRP5 fusion, and β-actin/ Vinculin in AW13516 (a), AW8507 (b), and NIH/3T3 (c) cells stably expressing empty vector or *UBE3C-LRP5* fusion (v7). *GAPDH* was used for the normalization of gene expression in the qRT-PCR data. The blots are from the same experiment and were processed in parallel. Data are shown as means ± SD. *p*-values are from Student's unpaired t-test and denoted as **, $p < 0.01$; ***, $p < 0.001$; ****, $p < 0.0001$. The data shown are representative of n = 3 independent experiments.

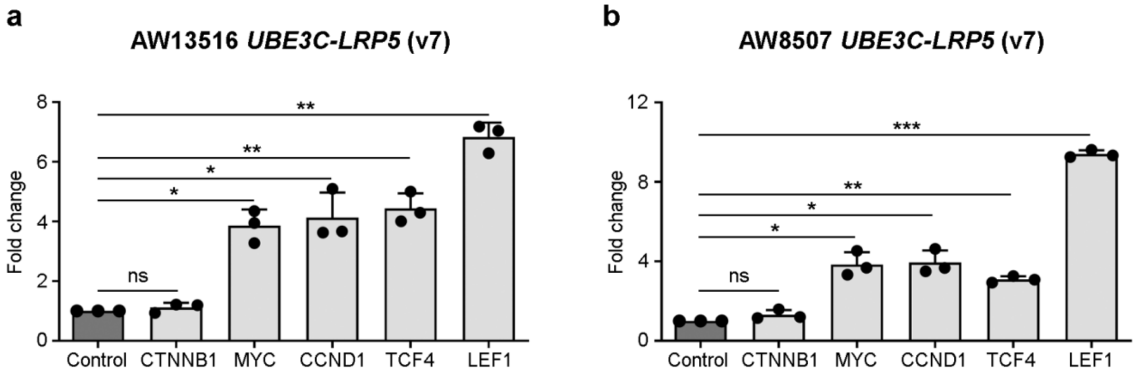

Supplementary Figure S15: *UBE3C-LRP5* fusion upregulates the Wnt signaling pathway genes. (a, b) Real-time PCR based fold change expression of *CTNNB1, MYC, CCND1, TCF4,* and *LEF1* in AW13516 (a) and AW8507 (b) cells stably overexpressing *UBE3C-LRP5* (v7) fusion compared to vector control cells. *GAPDH* was used for normalization. Data are shown as means ± SD. *p*-values are from Student's unpaired t-test and are denoted as *ns* (not significant); *, $p < 0.05$; **, $p < 0.01$; ***, $p < 0.001$. The data shown are representative of n = 3 independent experiments.

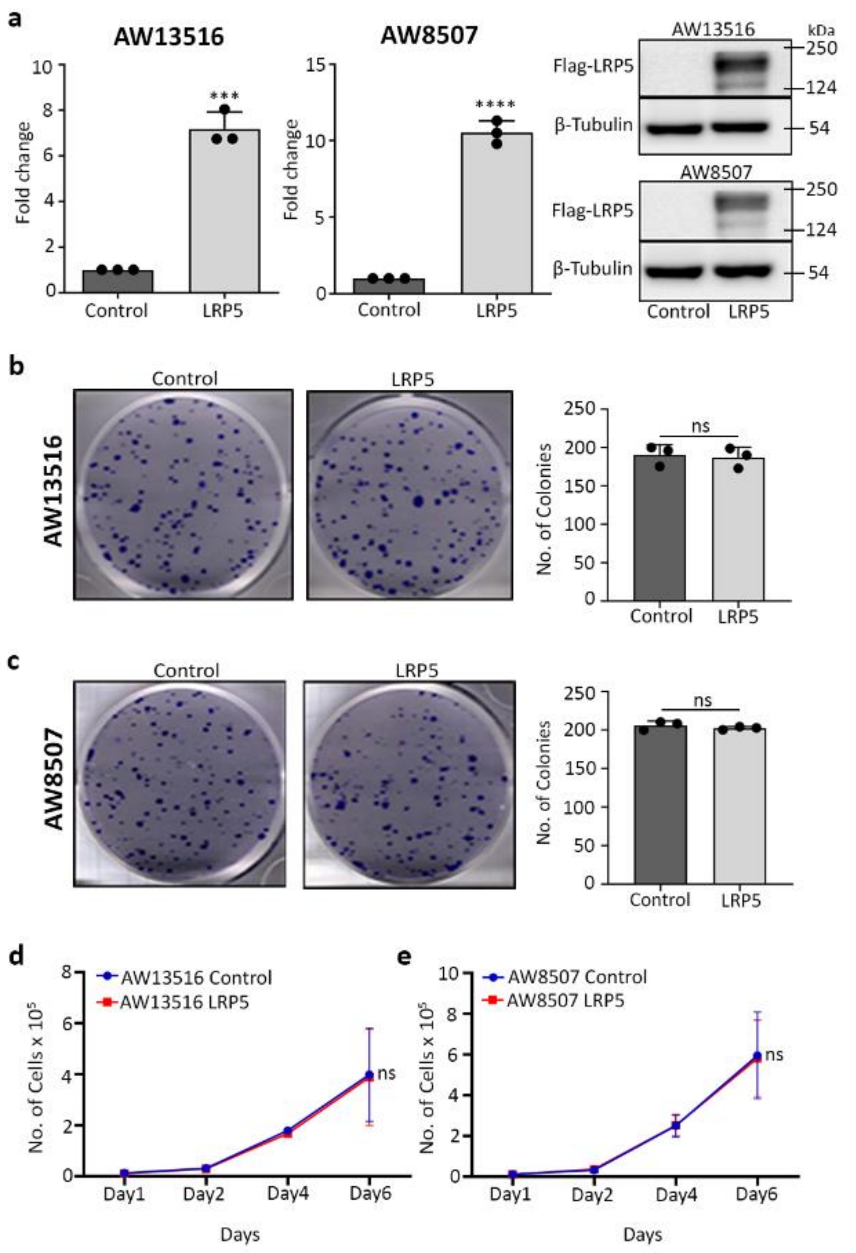

Supplementary Figure S16: Overexpression of *LRP5* has no significant effect on clonogenic and proliferative ability of HNSCC cells. (a) Real-time PCR of *LRP5* in AW13516 and AW8507 cells expressing empty vector or *LRP5*. Immunoblots of flag-tagged LRP5 and β-Tubulin in AW13516 and AW8507 cell lines expressing empty vector or *LRP5*. The blots are from the same experiment and were processed in parallel. (b, c) Clonogenic cell survival assay of AW13516 cells (b), and AW8507 cells (c) expressing empty vector or *LRP5*. (d, e) Cell proliferation assay of AW13516 cells (d), and AW8507 cells (e) expressing empty vector or *LRP5*.

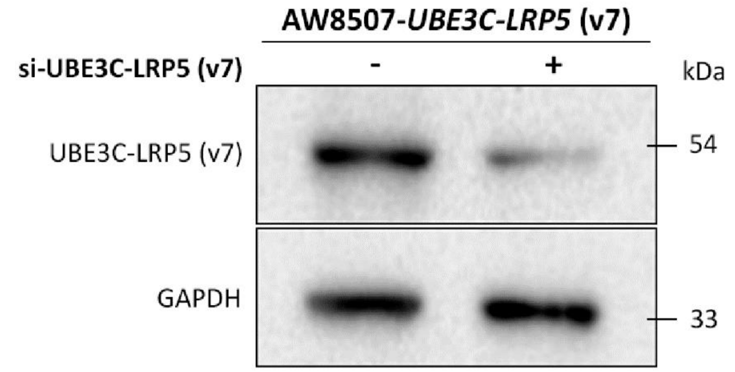

Supplementary Figure S17: Validation of *UBE3C-LRP5* (v7) fusion knockdown in AW8507-*UBE3C-LRP5* (v7) clones. Immunoblot of UBE3C-LRP5 (v7) fusion protein, and GAPDH in AW8507-*UBE3C-LRP5* (v7) clones. The blots are from the same experiment and were processed in parallel.

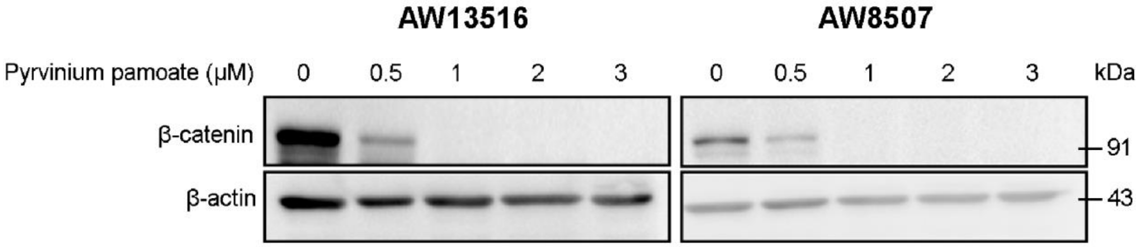

Supplementary Figure S18: Pyrvinium pamoate degrades β-catenin in a dose-dependent manner in head and neck cancer cell lines. Immunoblot of β-catenin and β-actin in AW13516 and AW8507 cells treated with different doses of pyrvinium pamoate mentioned on top of the gel images. The blots are from the same experiment and were processed in parallel. The data shown are representative of n = 3 independent experiments for each cell line.

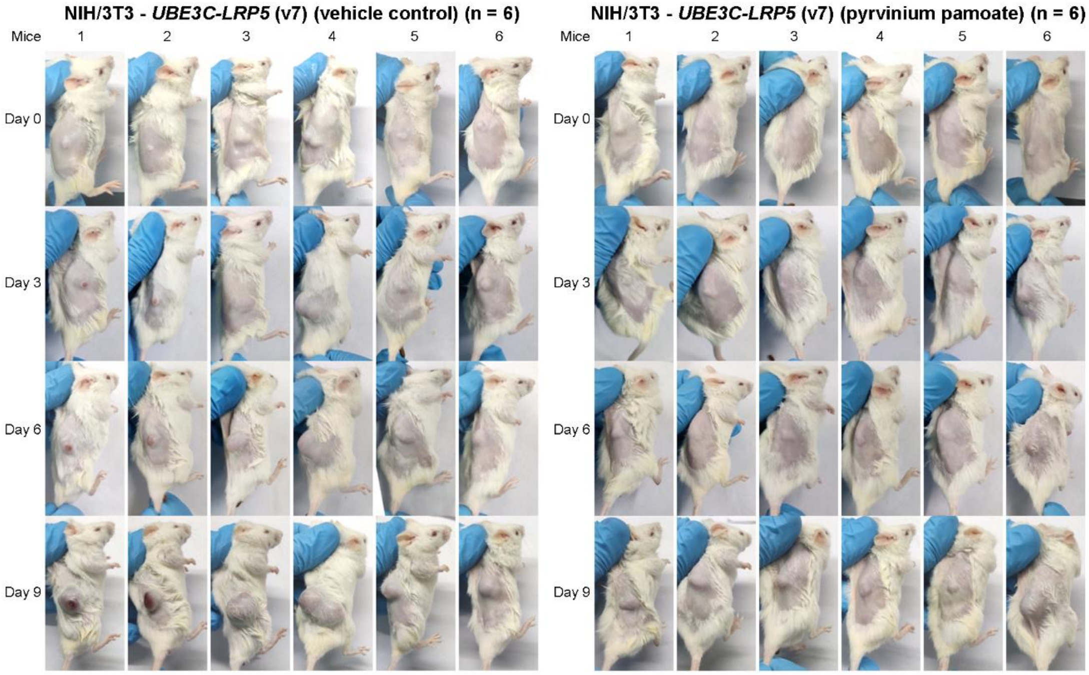

Supplementary Figure S19: Pyrvinium pamoate treatment suppresses tumor growth *in vivo*. Images of mice (n = 6 mice/group) showing tumors during (day 0 – day 9) the treatment with vehicle control or pyrvinium pamoate. Mice numbers are mentioned on top and days after start of the treatment are mentioned on the left side of the mice image panel.

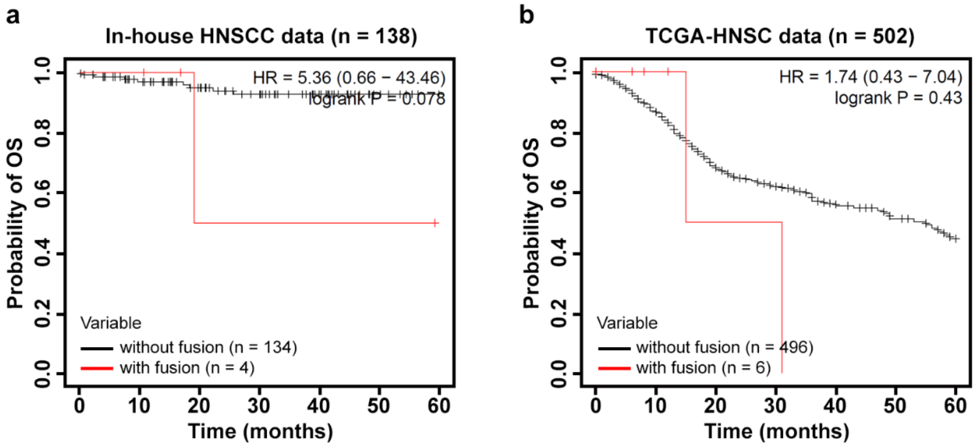

Supplementary Figure S20: Kaplan-Meier survival curves for overall survival in the in-house HNSCC data (n = 138) and TCGA-HNSC data (n = 502). (a, b) Kaplan-Meier (KM) survival curves for overall survival (OS) in the in-house HNSCC data (a), and TCGA-HNSC data (b) based on the presence or absence of *UBE3C-LRP5* fusion. The red and black lines denote the presence and absence of the fusion, respectively, in the KM plots. The number of samples in each group is denoted. The log-rank test was used to determine the statistical differences in median survival.

Supplementary Figure S21: Uncropped Western blots used in Figure 2e. Cropped sections marked on the blots are used as figures in the manuscript:

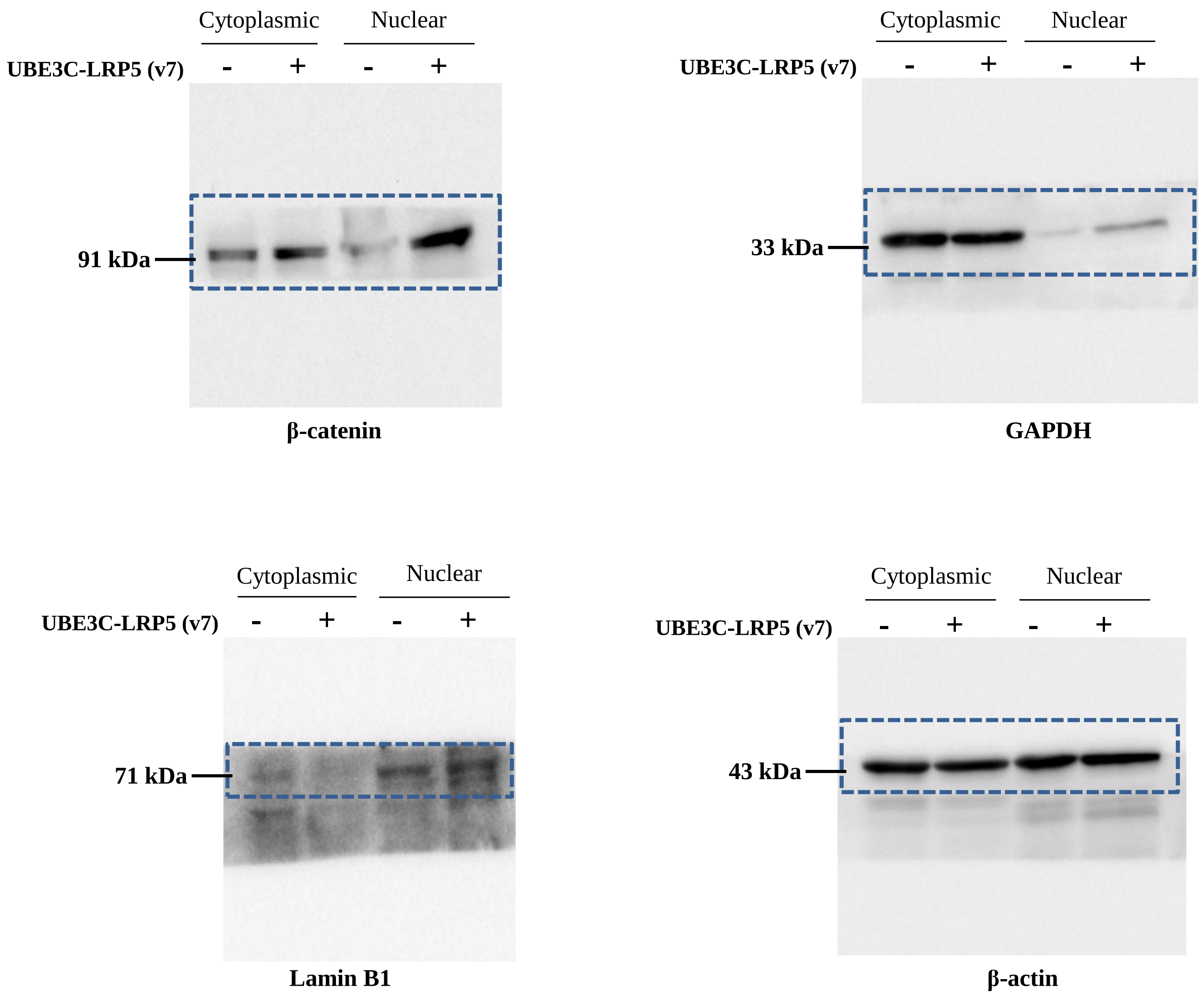

Supplementary Figure S22: Uncropped Western blots used in Figure 2f. Cropped sections marked on the blots are used as figures in the manuscript:

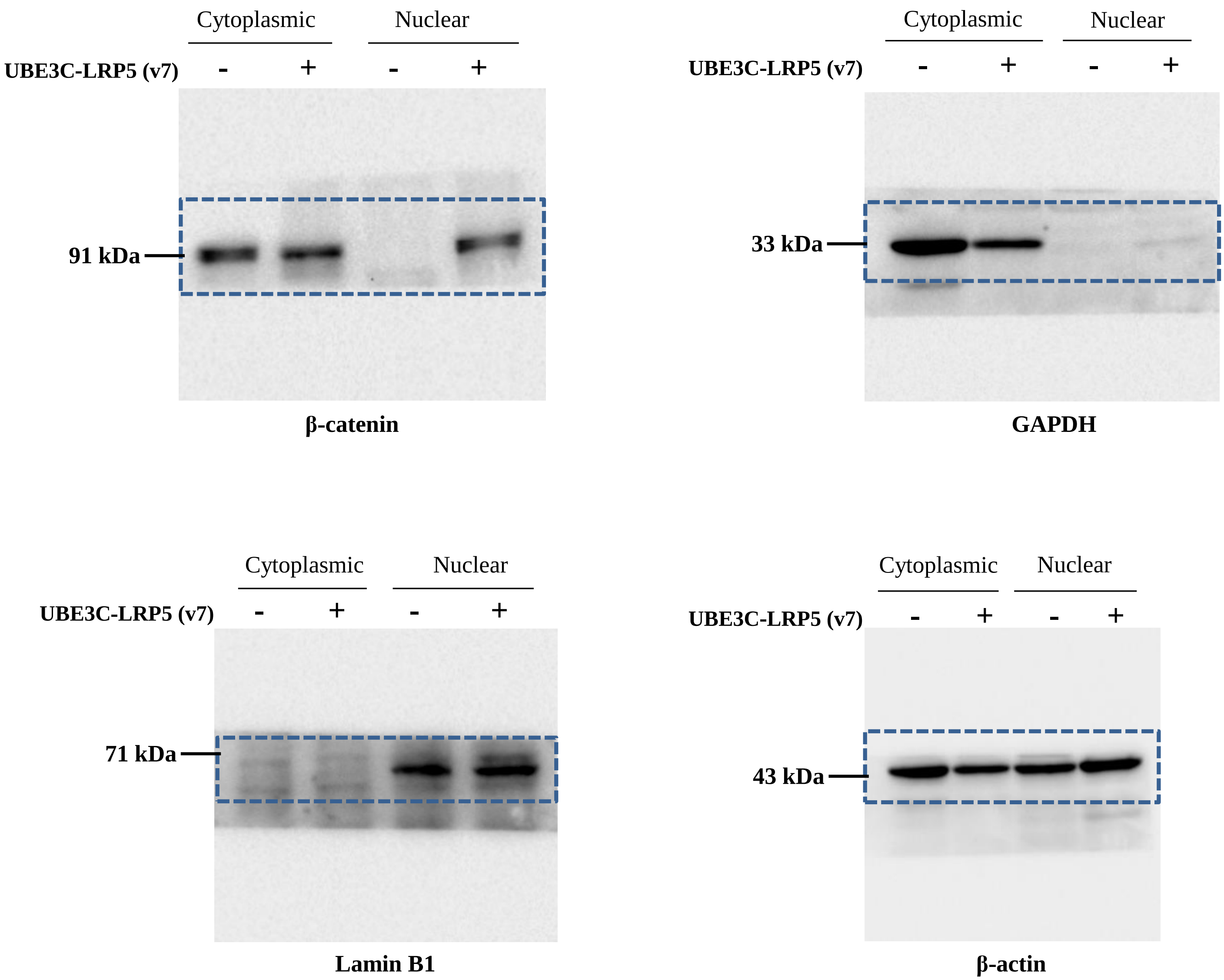

Supplementary Figure S23: Uncropped Western blots used in Supplementary Figure S12. Cropped sections marked on the blots are used as figures in the manuscript:

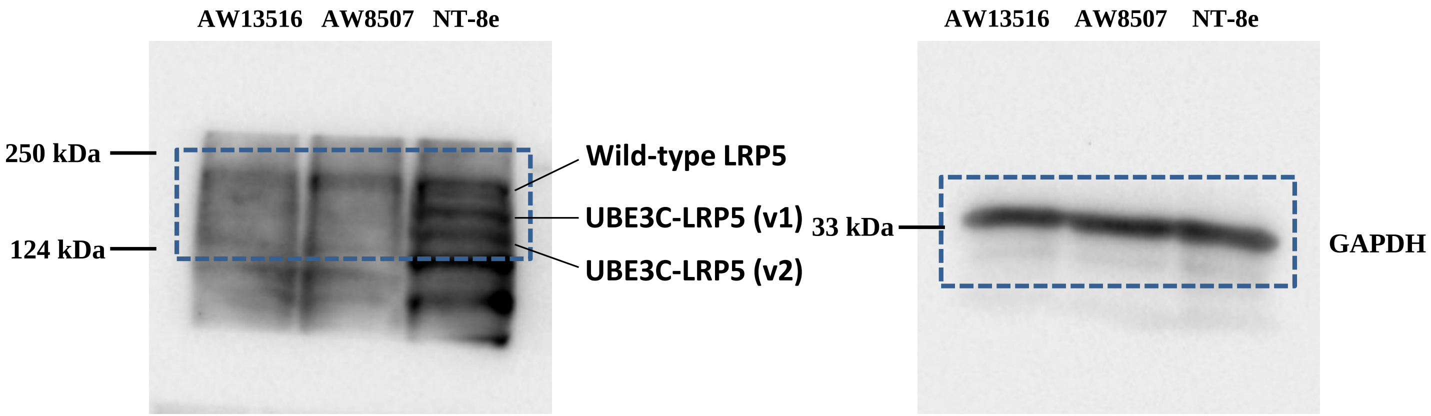

Supplementary Figure S24: Uncropped Western blots used in Supplementary Figure S14a, b. Cropped sections marked on the blots are used as figures in the manuscript:

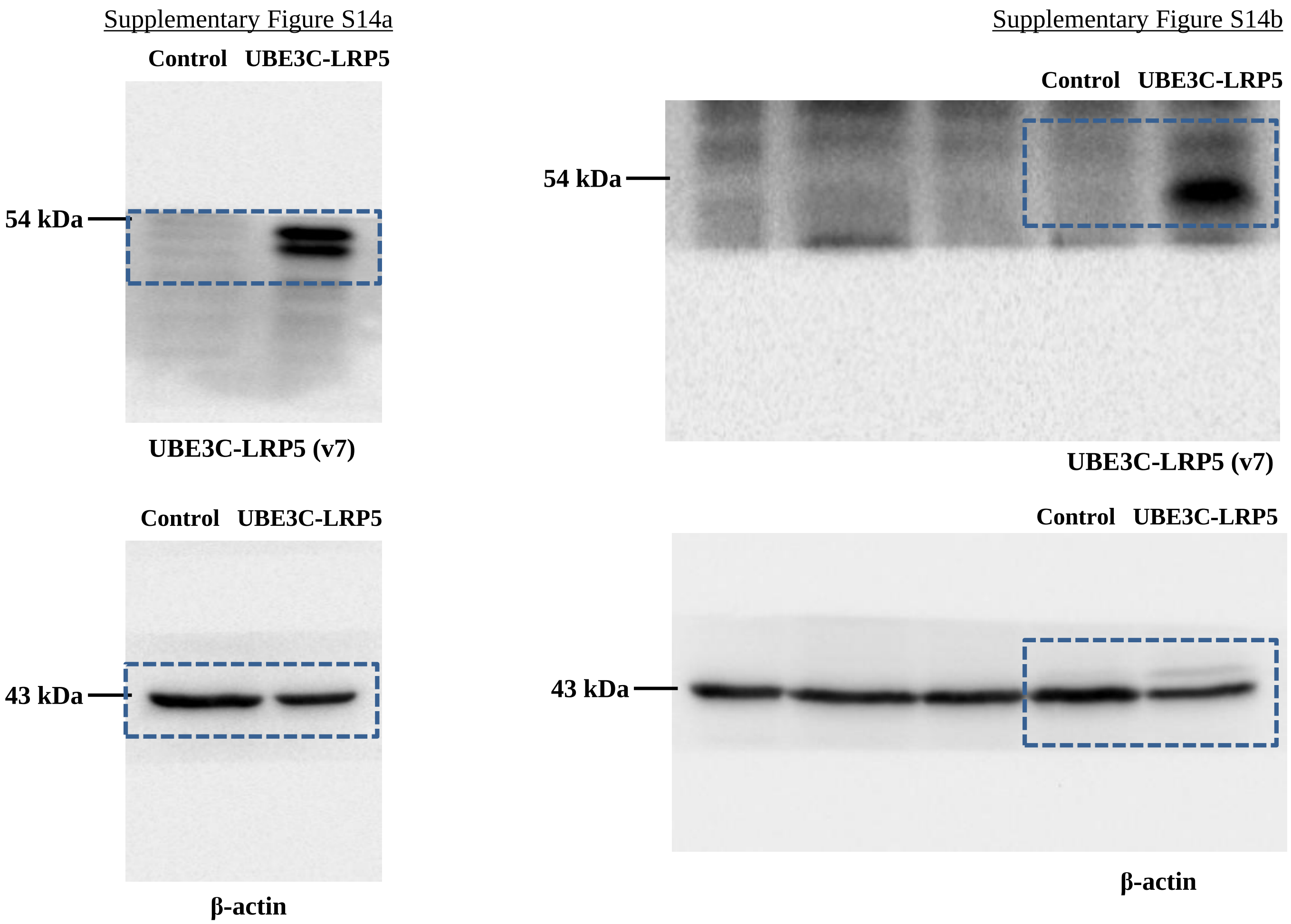

Supplementary Figure S25: Uncropped Western blots used in Supplementary Figure S14c. Cropped sections marked on the blots are used as figures in the manuscript:

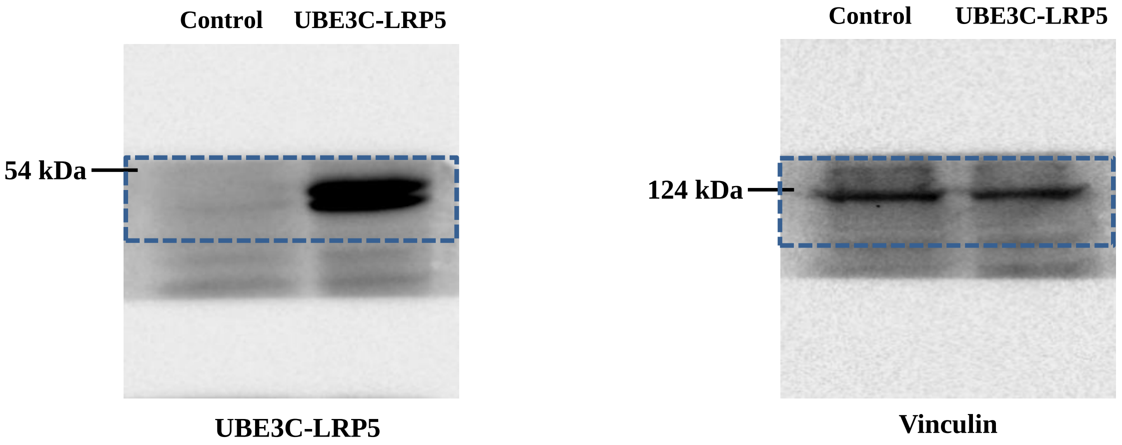

Supplementary Figure S26: Uncropped Western blots used in Supplementary Figure S16a. Cropped sections marked on the blots are used as figures in the manuscript:

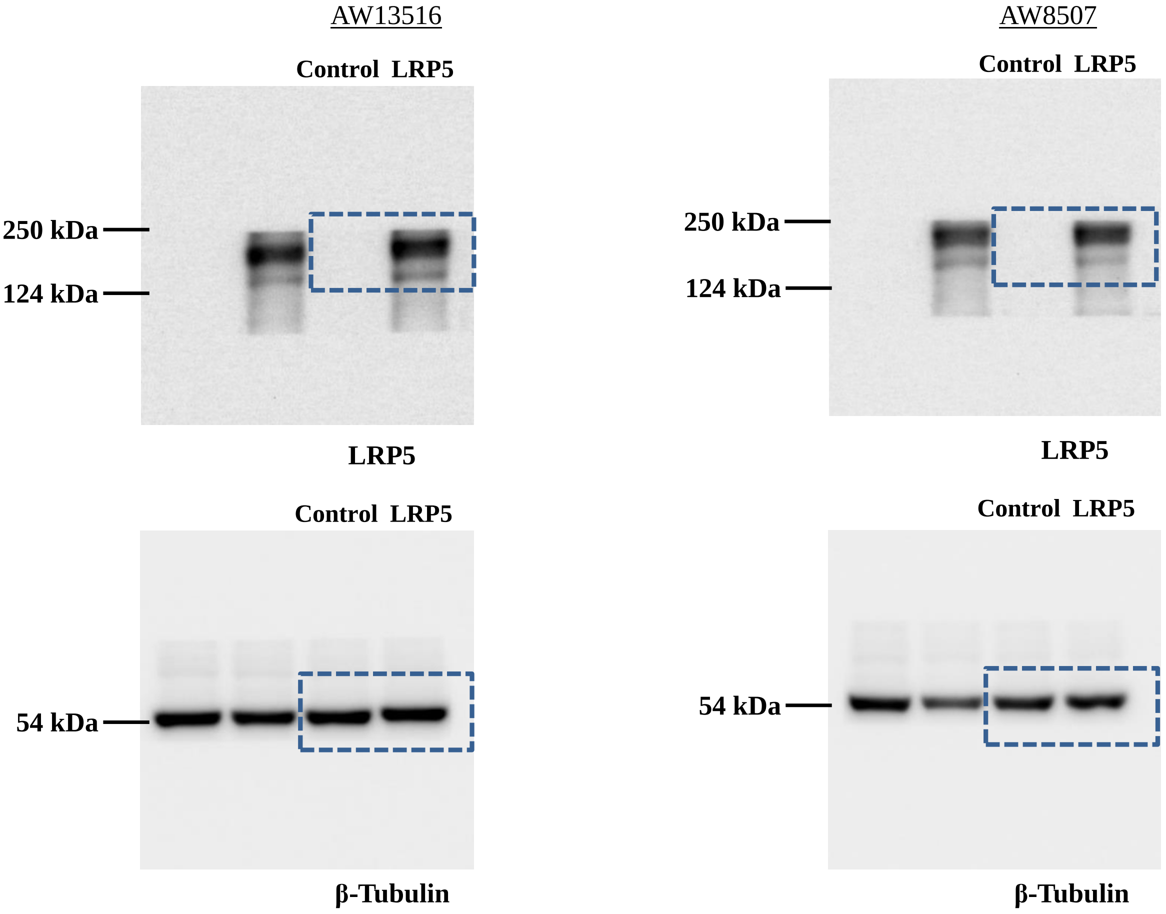

Supplementary Figure S27: Uncropped Western blots used in Supplementary Figure S17. Cropped sections marked on the blots are used as figures in the manuscript:

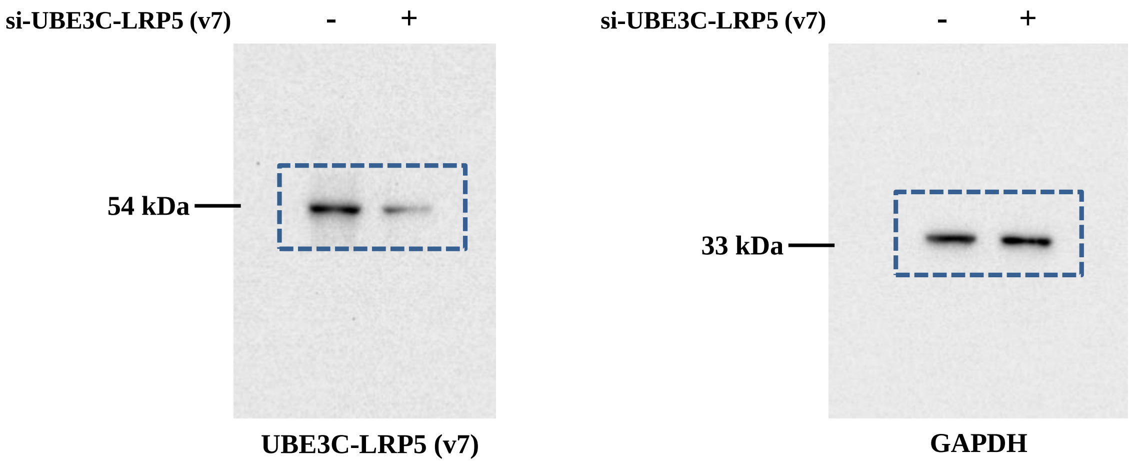

Supplementary Figure S28: Uncropped Western blots used in Supplementary Figure S18. Cropped sections marked on the blots are used as figures in the manuscript:

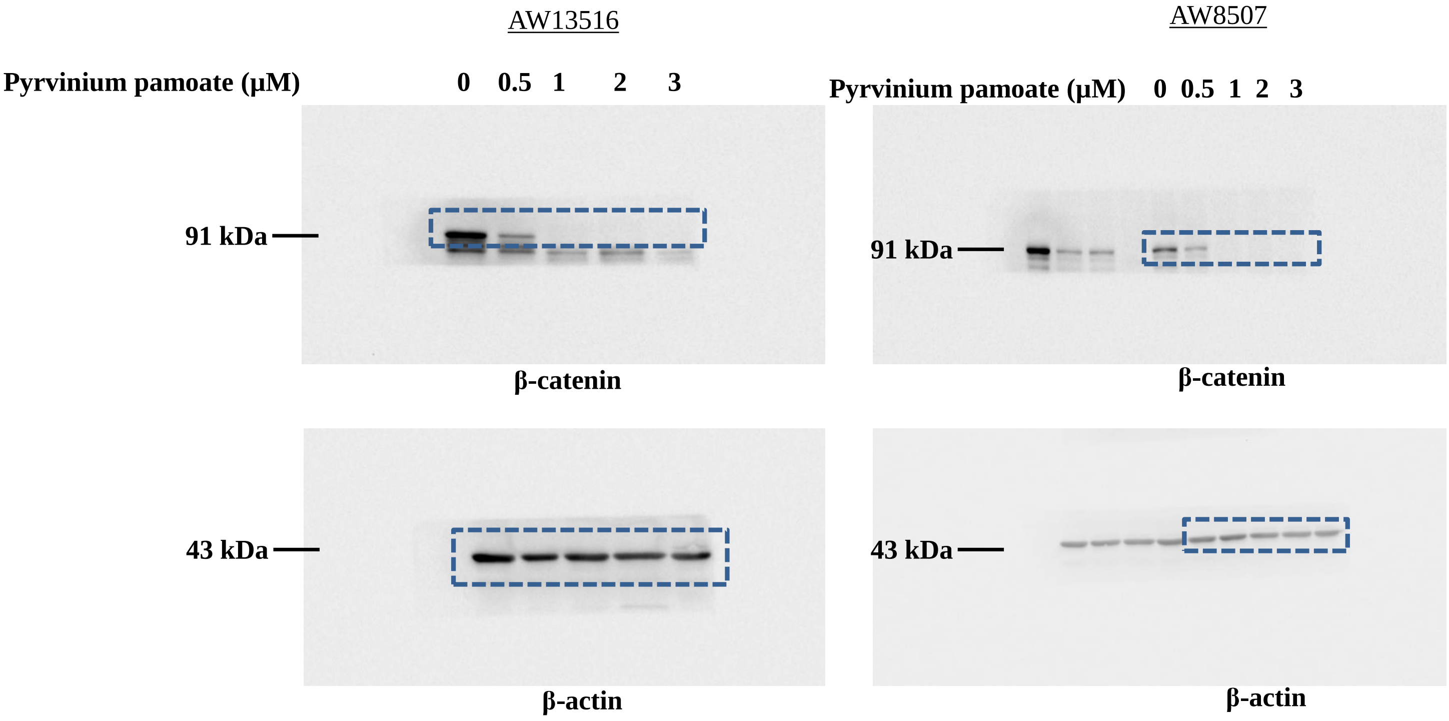

Supplementary Table S1: Primer sequences used for validation of fusion transcripts

S.No.	Primer ID	Name	Direction	Primer sequence	Remarks
1	OAD422	*LRP5-UBE3C*	F	AAGCTCTACTGGGCTGACGC	Primers used for validation of fusion transcripts at RNA level
	OAD423	*LRP5-UBE3C*	R	AGCGGCACATTCAATCGCATA	
2	OAD511	*UBE3C-LRP5*	F	TCAGCTCAGCTCAAAGCCAGCGGC	
	OAD2087	*UBE3C-LRP5*	R	CTCGTCCAGGTCCTCCGACA	
3	OAD418	*EXT1-MED30*	F	CTTGGCCTGACTACACCGAG	
	OAD419	*EXT1-MED30*	R	TAGTTCCTCATTGCCAACATGG	
4	OAD426	*YIFA1-RCOR2*	F	AGTATTCGTACCCACGAGGCG	
	OAD427	*YIFA1-RCOR2*	R	GTACCTGGCGCTTGAGGGAG	
5	OAD432	*PSMD5-VAV2*	F	CCCCACTATGATTGGTGTAGC	
	OAD433	*PSMD5-VAV2*	R	CGGGACTTGTAGGGGTACTTG	
6	OAD436	*FTSJD2-BTBD9*	F	GAATGCACTGCCCCCATCAA	
	OAD437	*FTSJD2-BTBD9*	R	CCTGTCCCCGATTCCTCACT	
7	OAD438	*NAIP-GTF2H2B*	F	CTCTGTCACCAAGTGCTCCATA	
	OAD439	*NAIP-GTF2H2B*	R	TAAAGGTGGCGCATCATTCC	
8	OAD1366	*CLN6-CALML4*	F	AGGCATGGCTCTGTGAGCG	
	OAD1367	*CLN6-CALML4*	R	CCGTCCAGGAAGGGTGATCTT	
9	OAD1368	*RRM2-C2orf48*	F	GAGGGTTTACACTGTGATTTTGC	
	OAD1369	*RRM2-C2orf48*	R	AGACCTTCCCGAGCTTCTCAT	
10	OAD1370	*SLC39A1-CRTC2*	F	CAGAAAGCCCTGAGCCTAGT	
	OAD1371	*SLC39A1-CRTC2*	R	AGGGAGAGCTGTCAATGTGG	
11	OAD1372	*POLA2-CDC42EP2*	F	CGGAGTGATCTTCGGCTTGA	
	OAD1373	*POLA2-CDC42EP2*	R	CACGTTCTCCCCAAAGGACG	
12	OAD1374	*CTSC-RAB38*	F	ACTTTGTGAAAGCTATCAATGCCA	
	OAD1375	*CTSC-RAB38*	R	TCCACTTTGCCACTGCTTCA	
13	OAD1376	*BACH1-GRIK1-AS2*	F	TGAGACGGACACCGAAGGAG	
	OAD1377	*BACH1-GRIK1-AS2*	R	TCTCTCCCATCTTCTGGCTTC	
14	OAD1941	*UBE3C-LRP5*	F1	TAGCCAGGTGTGGTGGTCTG	Primers for validation of fusion breakpoint at genomic DNA level
	OAD1943	*UBE3C-LRP5*	R1	CATACCAACAGTCGGCCTCAT	
15	OAD423	*UBE3C-LRP5*	F2	AGCGGCACATTCAATCGCATA	
	OAD554	*UBE3C-LRP5*	R2	AGATCCTCCGTAGGTCCGTC	
16	OAD1615	*GAPDH*	F	AATCCCATCACCATCTTCCA	Primers for *GAPDH* (reference control)
	OAD1616	*GAPDH*	R	TGGACTCCACGACGTACTCA	

Supplementary Table S2: Primer sequences used for real-time PCR

S.No.	Primer ID	Name	Direction	Primer sequence
1	OAD2483	*LRP5*	F	CCCTTCACAGGCATCGCATG
	OAD2484	*LRP5*	R	CCTGTGACGTGGTTCCGGTC
2	OAD1640	*LRP5-UBE3C* (v1)	F	GGACGTGTAAGGCAGGAGTT
	OAD1641	*LRP5-UBE3C* (v1)	R	TTCAGCAGGTTCATGCAGGT
3	OAD2142	*UBE3C-LRP5* (v1)	F	AACTCCTGTCTCCACAGGAGC
	OAD2087	*UBE3C-LRP5* (v1)	R	CTCGTCCAGGTCCTCCGACA
4	OAD2143	*UBE3C-LRP5* (v2)	F	GATACAACTCCTGGACGGGT
	OAD2087	*UBE3C-LRP5* (v2)	R	CTCGTCCAGGTCCTCCGACA
5	OAD2483	*UBE3C-LRP5* (v7)	F	CCCTTCACAGGCATCGCATG
	OAD2484	*UBE3C-LRP5* (v7)	R	CCTGTGACGTGGTTCCGGTC
6	OAD559	*CTNNB1*	F	GCTGGGACCTTGCATAACCTT
	OAD560	*CTNNB1*	R	ATTTTCACCAGGGCAGGAATG
7	OAD561	*LEF1*	F	TCGTGAAGAGCAGGCTAA
	OAD562	*LEF1*	R	GCAGACCAGCCTGGATA
8	OAD563	*TCF4*	F	ACATGCATGGAATCATTGGA
	OAD564	*TCF4*	R	TGAATGTCTGTTGGCTGAAA
9	OAD205	*MYC*	F	TAGTGGAAAACCAGCAGCCT
	OAD206	*MYC*	R	TCGTCGCAGTAGAAATACGG
10	OAD226	*CCND1*	F	CGTGGCCTCTAAGATGAAGG
	OAD227	*CCND1*	R	CCACTTGAGCTTGTTCACCA
11	OAD1615	*GAPDH*	F	AATCCCATCACCATCTTCCA
	OAD1616	*GAPDH*	R	TGGACTCCACGACGTACTCA
12	OAD1642	Mice_*Gapdh*	F	AAGCCCATCACCATCTTCCA
	OAD1643	Mice_*Gapdh*	R	GTAGACTCCACGACATACTCAGC

Supplementary Table S3: cDNA sequences of cloned *LRP5-UBE3C* (v1) and *UBE3C-LRP5* (v1, v2, v7) fusion transcript variants

1) cDNA sequence of *LRP5-UBE3C* (v1) fusion transcript:

ATGGAGGCAGCGCCGCCCGGGCCGCCGTGGCCGCTGCTGCTGCTGCTGCTGCTGCTGCTGGCGCTGTGCGGC
TGCCCGGCCCCCGCCGCGGCCTCGCCGCTCCTGCTATTTGCCAACCGCCGGGACGTACGGCTGGTGGACGCC
GGCGGAGTCAAGCTGGAGTCCACCATCGTGGTCAGCGGCCTGGAGGATGCGGCCGCAGTGGACTTCCAGTTT
TCCAAGGGAGCCGTGTACTGGACAGACGTGAGCGAGGAGGCCATCAAGCAGACCTACCTGAACCAGACGGG
GGCCGCCGTGCAGAACGTGGTCATCTCCGGCCTGGTCTCTCCCGACGGCCTCGCCTGCGACTGGGTGGGCAA
GAAGCTGTACTGGACGGACTCAGAGACCAACCGCATCGAGGTGGCCAACCTCAATGGCACATCCCGGAAGGT
GCTCTTCTGGCAGGACCTTGACCAGCCGAGGGCCATCGCCTTGGACCCCGCTCACGGGTACATGTACTGGACA
GACTGGGGTGAGACGCCCCGGATTGAGCGGGCAGGGATGGATGGCAGCACCCGGAAGATCATTGTGGACTC
GGACATTTACTGGCCCAATGGACTGACCATCGACCTGGAGGAGCAGAAGCTCTACTGGGCTGACGCCAAGCT
CAGCTTCATCCACCGTGCCAACCTGGACGGCTCGTTCCGGCAGAAGGTGGTGGAGGGCAGCCTGACGCACCC
CTTCGCCCTGACGCTCTCCGGGGACACTCTGTACTGGACAGACTGGCAGACCCGCTCCATCCATGCCTGCAAC
AAGCGCACTGGGGGGAAGAGGAAGGAGATCCTGAGTGCCCTCTACTCACCCATGGACATCCAGGTGCTGAG
CCAGGAGCGGCAGCCTTTCTTCCACACTCGCTGTGAGGAGGACAATGGCGGCTGCTCCCACCTGTGCCTGCTG
TCCCCAAGCGAGCCTTTCTACACATGCGCCTGCCCCACGGGTGTGCAGCTGCAGGACAACGGCAGGACGTGT
AAGGCAGGAGTTGTATCCCGCATTTTGTATTCACAACGGAGGCTCCGACCT**TGA**GCGGCTCCCCACAGCCAGC
ACCTGCATGAACCTGCTGAAGCTCCCCGAGTTCTATGACGAGACACTTTTGCGAAGTAAACTTCTCTATGCGAT
TGAATGTGCCGCTGGCTTTGAGCTGAGCTGA

2) cDNA sequence of *UBE3C-LRP5* (v1) fusion transcript:

TCAGCTCAGCTCAAAGCCAGCGGCACATTCAATCGCATAGAGAAGTTTACTTCGCAAAAGTGTCTCGTCATAG
AACTCGGGGAGCTTCAGCAGGTTCATGCAGGTGCTGGCTGTGGGGAGCCGCTCAAGGTCGGAGCCTCCGTTG
TGAATACAAA**ATG**CGGGATACAACTCCTGTCTCCACAGGAGCCGAGGAGGTGCTGCTGCTGGCCCGGCGGAC
GGACCTACGGAGGATCTCGCTGGACACGCCGGACTTCACCGACATCGTGCTGCAGGTGGACGACATCCGGCA
CGCCATTGCCATCGACTACGACCCGCTAGAGGGCTATGTCTACTGGACAGATGACGAGGTGCGGGCCATCCG
CAGGGCGTACCTGGACGGGTCTGGGGCGCAGACGCTGGTCAACACCGAGATCAACGACCCCGATGGCATCG
CGGTCGACTGGGTGGCCCGAAACCTCTACTGGACCGACACGGGCACGGACCGCATCGAGGTGACGCGCCTCA
ACGGCACCTCCCGCAAGATCCTGGTGTCGGAGGACCTGGACGAGCCCCGAGCCATCGCACTGCACCCCGTGA
TGGGCCTCATGTACTGGACAGACTGGGGAGAGAACCCTAAAATCGAGTGTGCCAACTTGGATGGGCAGGAG
CGGCGTGTGCTGGTCAATGCCTCCCTCGGGTGGCCCAACGGCCTGGCCCTGGACCTGCAGGAGGGGAAGCTC
TACTGGGGAGACGCCAAGACAGACAAGATCGAGGTGATCAATGTTGATGGGACGAAGAGGCGGACCCTCCT
GGAGGACAAGCTCCCGCACATTTTTGGGTTCACGCTGCTGGGGGACTTCATCTACTGGACTGACTGGCAGCGC
CGCAGCATCGAGCGGGTGCACAAGGTCAAGGCCAGCCGGGACGTCATCATTGACCAGCTGCCCGACCTGATG
GGGCTCAAAGCTGTGAATGTGGCCAAGGTCGTCGGAACCAACCCGTGTGCGGACAGGAACGGGGGGTGCAG
CCACCTGTGCTTCTTCACACCCCACGCAACCCGGTGTGGCTGCCCCATCGGCCTGGAGCTGCTGAGTGACATG
AAGACCTGCATCGTGCCTGAGGCCTTCTTGGTCTTCACCAGCAGAGCCGCCATCCACAGGATCTCCCTCGAGA
CCAATAACAACGACGTGGCCATCCCGCTCACGGGCGTCAAGGAGGCCTCAGCCCTGGACTTTGATGTGTCCAA
CAACCACATCTACTGGACAGACGTCAGCCTGAAGACCATCAGCCGCGCCTTCATGAACGGGAGCTCGGTGGA
GCACGTGGTGGAGTTTGGCCTTGACTACCCCGAGGGCATGGCCGTTGACTGGATGGGCAAGAACCTCTACTG
GGCCGACACTGGGACCAACAGAATCGAAGTGGCGCGGCTGGACGGGCAGTTCCGGCAAGTCCTCGTGTGGA
GGGACTTGGACAACCCGAGGTCGCTGGCCCTGGATCCCACCAAGGGCTACATCTACTGGACCGAGTGGGGCG
GCAAGCCGAGGATCGTGCGGGCCTTCATGGACGGGACCAACTGCATGACGCTGGTGGACAAGGTGGGCCGG
GCCAACGACCTCACCATTGACTACGCTGACCAGCGCCTCTACTGGACCGACCTGGACACCAACATGATCGAGT
CGTCCAACATGCTGGGTCAGGAGCGGGTCGTGATTGCCGACGATCTCCCGCACCCGTTCGGTCTGACGCAGTA
CAGCGATTATATCTACTGGACAGACTGGAATCTGCACAGCATTGAGCGGGCCGACAAGACTAGCGGCCGGAA

CCGCACCCTCATCCAGGGCCACCTGGACTTCGTGATGGACATCCTGGTGTTCCACTCCTCCCGCCAGGATGGCC
TCAATGACTGTATGCACAACAACGGGCAGTGTGGGCAGCTGTGCCTTGCCATCCCCGGCGGCCACCGCTGCG
GCTGCGCCTCACACTACACCCTGGACCCCAGCAGCCGCAACTGCAGCCCGCCCACCACCTTCTTGCTGTTCAGC
CAGAAATCTGCCATCAGTCGGATGATCCCGGACGACCAGCACAGCCCGGATCTCATCCTGCCCCTGCATGGAC
TGAGGAACGTCAAAGCCATCGACTATGACCCACTGGACAAGTTCATCTACTGGGTGGATGGGCGCCAGAACA
TCAAGCGAGCCAAGGACGACGGGACCCAGCCCTTTGTTTTGACCTCTCTGAGCCAAGGCCAAAACCCAGACA
GGCAGCCCCACGACCTCAGCATCGACATCTACAGCCGGACACTGTTCTGGACGTGCGAGGCCACCAATACCAT
CAACGTCCACAGGCTGAGCGGGGAAGCCATGGGGGTGGTGCTGCGTGGGGACCGCGACAAGCCCAGGGCC
ATCGTCGTCAACGCGGAGCGAGGGTACCTGTACTTCACCAACATGCAGGACCGGGCAGCCAAGATCGAACGC
GCAGCCCTGGACGGCACCGAGCGCGAGGTCCTCTTCACCACCGGCCTCATCCGCCCTGTGGCCCTGGTGGTG
GACAACACACTGGGCAAGCTGTTCTGGGTGGACGCGGACCTGAAGCGCATTGAGAGCTGTGACCTGTCAGG
GGCCAACCGCCTGACCCTGGAGGACGCCAACATCGTGCAGCCTCTGGGCCTGACCATCCTTGGCAAGCATCTC
TACTGGATCGACCGCCAGCAGCAGATGATCGAGCGTGTGGAGAAGACCACCGGGGACAAGCGGACTCGCAT
CCAGGGCCGTGTCGCCCACCTCACTGGCATCCATGCAGTGGAGGAAGTCAGCCTGGAGGAGTTCTCAGCCCA
CCCATGTGCCCGTGACAATGGTGGCTGCTCCCACATCTGTATTGCCAAGGGTGATGGGACACCACGGTGCTCA
TGCCCAGTCCACCTCGTGCTCCTGCAGAACCTGCTGACCTGTGGAGAGCCGCCCACCTGCTCCCCGGACCAGT
TTGCATGTGCCACAGGGGAGATCGACTGTATCCCCGGGGCCTGGCGCTGTGACGGCTTTCCCGAGTGCGATG
ACCAGAGCGACGAGGAGGGCTGCCCCGTGTGCTCCGCCGCCCAGTTCCCCTGCGCGCGGGGTCAGTGTGTGG
ACCTGCGCCTGCGCTGCGACGGCGAGGCAGACTGTCAGGACCGCTCAGACGAGGCGGACTGTGACGCCATCT
GCCTGCCCAACCAGTTCCGGTGTGCGAGCGGCCAGTGTGTCCTCATCAAACAGCAGTGCGACTCCTTCCCCGA
CTGTATCGACGGCTCCGACGAGCTCATGTGTGAAATCACCAAGCCGCCCTCAGACGACAGCCCGGCCCACAGC
AGTGCCATCGGGCCCGTCATTGGCATCATCCTCTCTCTCTTCGTCATGGGTGGTGTCTATTTTGTGTGCCAGCG
CGTGGTGTGCCAGCGCTATGCGGGGGCCAACGGGCCCTTCCCGCACGAGTATGTCAGCGGGACCCCGCACGT
GCCCCTCAATTTCATAGCCCCGGGCGGTTCCCAGCATGGCCCCTTCACAGGCATCGCATGCGGAAAGTCCATG
ATGAGCTCCGTGAGCCTGATGGGGGGCCGGGGCGGGGTGCCCCTCTACGACCGGAACCACGTCACAGGGGC
CTCGTCCAGCAGCTCGTCCAGCACGAAGGCCACGCTGTACCCGCCGATCCTGAACCCGCCGCCCTCCCCGGCC
ACGGACCCCTCCCTGTACAACATGGACATGTTCTACTCTTCAAACATTCCGGCCACTGCGAGACCGTACAGGCC
CTACATCATTCGAGGAATGGCGCCCCCGACGACGCCCTGCAGCACCGACGTGTGTGACAGCGACTACAGCGC
CAGCCGCTGGAAGGCCAGCAAGTACTACCTGGATTTGAACTCGGACTCAGACCCCTATCCACCCCCACCCACG
CCCCACAGCCAGTACCTGTCGGCGGAGGACAGCTGCCCGCCCTCGCCCGCCACCGAGAGGAGCTACTTCCATC
TCTTCCCGCCCCCTCCGTCCCCCTGCACGGACTCATCC**TGA**

3) cDNA sequence of *UBE3C-LRP5* (v2) fusion transcript:

TCAGCTCAGCTCAAAGCCAGCGGCACATTCAATCGCATAGAGAAGTTTACTTCGCAAAAGTGTCTCGTCATAG
AACTCGGGGAGCTTCAGCAGGTTCATGCAGGTGCTGGCTGTGGGGAGCCGCTCAAGGTCGGAGCCTCCGTTG
TGAATACAAAATGCGGGATACAACTCCTGGACGGGTCTGGGGCGCAGACGCTGGTCAACACCGAGATCAACG
ACCCCGATGGCATCGCGGTCGACTGGGTGGCCCGAAACCTCTACTGGACCGACACGGGCACGGACCGCATCG
AGGTGACGCGCCTCAACGGCACCTCCCGCAAGATCCTGGTGTCGGAGGACCTGGACGAGCCCCGAGCCATCG
CACTGCACCCCGTG**ATG**GGCCTCATGTACTGGACAGACTGGGGAGAGAACCCTAAAATCGAGTGTGCCAACT
TGGATGGGCAGGAGCGGCGTGTGCTGGTCAATGCCTCCCTCGGGTGGCCCAACGGCCTGGCCCTGGACCTGC
AGGAGGGGAAGCTCTACTGGGGAGACGCCAAGACAGACAAGATCGAGGTGATCAATGTTGATGGGACGAA
GAGGCGGACCCTCCTGGAGGACAAGCTCCCGCACATTTTTGGGTTCACGCTGCTGGGGGACTTCATCTACTGG
ACTGACTGGCAGCGCCGCAGCATCGAGCGGGTGCACAAGGTCAAGGCCAGCCGGGACGTCATCATTGACCA
GCTGCCCGACCTGATGGGGCTCAAAGCTGTGAATGTGGCCAAGGTCGTCGGAACCAACCCGTGTGCGGACAG
GAACGGGGGGTGCAGCCACCTGTGCTTCTTCACACCCCACGCAACCCGGTGTGGCTGCCCCATCGGCCTGGA
GCTGCTGAGTGACATGAAGACCTGCATCGTGCCTGAGGCCTTCTTGGTCTTCACCAGCAGAGCCGCCATCCAC
AGGATCTCCCTCGAGACCAATAACAACGACGTGGCCATCCCGCTCACGGGCGTCAAGGAGGCCTCAGCCCTG
GACTTTGATGTGTCCAACAACCACATCTACTGGACAGACGTCAGCCTGAAGACCATCAGCCGCGCCTTCATGA

ACGGGAGCTCGGTGGAGCACGTGGTGGAGTTTGGCCTTGACTACCCCGAGGGCATGGCCGTTGACTGGATG
GGCAAGAACCTCTACTGGGCCGACACTGGGACCAACAGAATCGAAGTGGCGCGGCTGGACGGGCAGTTCCG
GCAAGTCCTCGTGTGGAGGGACTTGGACAACCCGAGGTCGCTGGCCCTGGATCCCACCAAGGGCTACATCTA
CTGGACCGAGTGGGGCGGCAAGCCGAGGATCGTGCGGGCCTTCATGGACGGGACCAACTGCATGACGCTGG
TGGACAAGGTGGGCCGGGCCAACGACCTCACCATTGACTACGCTGACCAGCGCCTCTACTGGACCGACCTGG
ACACCAACATGATCGAGTCGTCCAACATGCTGGGTCAGGAGCGGGTCGTGATTGCCGACGATCTCCCGCACCC
GTTCGGTCTGACGCAGTACAGCGATTATATCTACTGGACAGACTGGAATCTGCACAGCATTGAGCGGGCCGA
CAAGACTAGCGGCCGGAACCGCACCCTCATCCAGGGCCACCTGGACTTCGTGATGGACATCCTGGTGTTCCAC
TCCTCCCGCCAGGATGGCCTCAATGACTGTATGCACAACAACGGGCAGTGTGGGCAGCTGTGCCTTGCCATCC
CCGGCGGCCACCGCTGCGGCTGCGCCTCACACTACACCCTGGACCCCAGCAGCCGCAACTGCAGCCCGCCCAC
CACCTTCTTGCTGTTCAGCCAGAAATCTGCCATCAGTCGGATGATCCCGGACGACCAGCACAGCCCGGATCTC
ATCCTGCCCCTGCATGGACTGAGGAACGTCAAAGCCATCGACTATGACCCACTGGACAAGTTCATCTACTGGG
TGGATGGGCGCCAGAACATCAAGCGAGCCAAGGACGACGGGACCCAGCCCTTTGTTTTGACCTCTCTGAGCC
AAGGCCAAAACCCAGACAGGCAGCCCCACGACCTCAGCATCGACATCTACAGCCGGACACTGTTCTGGACGT
GCGAGGCCACCAATACCATCAACGTCCACAGGCTGAGCGGGGAAGCCATGGGGGTGGTGCTGCGTGGGGAC
CGCGACAAGCCCAGGGCCATCGTCGTCAACGCGGAGCGAGGGTACCTGTACTTCACCAACATGCAGGACCGG
GCAGCCAAGATCGAACGCGCAGCCCTGGACGGCACCGAGCGCGAGGTCCTCTTCACCACCGGCCTCATCCGC
CCTGTGGCCCTGGTGGTGGACAACACACTGGGCAAGCTGTTCTGGGTGGACGCGGACCTGAAGCGCATTGAG
AGCTGTGACCTGTCAGGGGCCAACCGCCTGACCCTGGAGGACGCCAACATCGTGCAGCCTCTGGGCCTGACC
ATCCTTGGCAAGCATCTCTACTGGATCGACCGCCAGCAGCAGATGATCGAGCGTGTGGAGAAGACCACCGGG
GACAAGCGGACTCGCATCCAGGGCCGTGTCGCCCACCTCACTGGCATCCATGCAGTGGAGGAAGTCAGCCTG
GAGGAGTTCTCAGCCCACCCATGTGCCCGTGACAATGGTGGCTGCTCCCACATCTGTATTGCCAAGGGTGATG
GGACACCACGGTGCTCATGCCCAGTCCACCTCGTGCTCCTGCAGAACCTGCTGACCTGTGGAGAGCCGCCCAC
CTGCTCCCCGGACCAGTTTGCATGTGCCACAGGGGAGATCGACTGTATCCCCGGGGCCTGGCGCTGTGACGG
CTTTCCCGAGTGCGATGACCAGAGCGACGAGGAGGGCTGCCCCGTGTGCTCCGCCGCCCAGTTCCCCTGCGC
GCGGGGTCAGTGTGTGGACCTGCGCCTGCGCTGCGACGGCGAGGCAGACTGTCAGGACCGCTCAGACGAGG
CGGACTGTGACGCCATCTGCCTGCCCAACCAGTTCCGGTGTGCGAGCGGCCAGTGTGTCCTCATCAAACAGCA
GTGCGACTCCTTCCCCGACTGTATCGACGGCTCCGACGAGCTCATGTGTGAAATCACCAAGCCGCCCTCAGAC
GACAGCCCGGCCCACAGCAGTGCCATCGGGCCCGTCATTGGCATCATCCTCTCTCTCTTCGTCATGGGTGGTG
TCTATTTTGTGTGCCAGCGCGTGGTGTGCCAGCGCTATGCGGGGGCCAACGGGCCCTTCCCGCACGAGTATGT
CAGCGGGACCCCGCACGTGCCCCTCAATTTCATAGCCCCGGGCGGTTCCCAGCATGGCCCCTTCACAGGCATC
GCATGCGGAAAGTCCATGATGAGCTCCGTGAGCCTGATGGGGGGCCGGGGCGGGGTGCCCCTCTACGACCG
GAACCACGTCACAGGGGCCTCGTCCAGCAGCTCGTCCAGCACGAAGGCCACGCTGTACCCGCCGATCCTGAA
CCCGCCGCCCTCCCCGGCCACGGACCCCTCCCTGTACAACATGGACATGTTCTACTCTTCAAACATTCCGGCCA
CTGCGAGACCGTACAGGCCCTACATCATTCGAGGAATGGCGCCCCCGACGACGCCCTGCAGCACCGACGTGT
GTGACAGCGACTACAGCGCCAGCCGCTGGAAGGCCAGCAAGTACTACCTGGATTTGAACTCGGACTCAGACC
CCTATCCACCCCCACCCACGCCCCACAGCCAGTACCTGTCGGCGGAGGACAGCTGCCCGCCCTCGCCCGCCAC
CGAGAGGAGCTACTTCCATCTCTTCCCGCCCCCTCCGTCCCCCTGCACGGACTCATCC**TGA**

4) cDNA sequence of *UBE3C-LRP5* (v7) fusion transcript:

GTGACCACACCGTAATAAAAATCATTTAAAAAAGGAAGGGAGGGAGGAATGGCAGAATGAAAGAAGGAGCT
TAGGAGAGCCTCAGGCGTGCAGTGTGAGTATCCTCGTGGGTCTGGGGAGGCGCTGCTGACGAAGGAAGCAC
TGGTTCTCTGTAGGGGTCTGACGAGCTC**ATG**TGTGAAATCACCAAGCCGCCCTCAGACGACAGCCCGGCCCAC
AGCAGTGCCATCGGGCCCGTCATTGGCATCATCCTCTCTCTCTTCGTCATGGGTGGTGTCTATTTTGTGTGCCA
GCGCGTGGTGTGCCAGCGCTATGCGGGGGCCAACGGGCCCTTCCCGCACGAGTATGTCAGCGGGACCCCGCA
CGTGCCCCTCAATTTCATAGCCCCGGGCGGTTCCCAGCATGGCCCCTTCACAGGCATCGCATGCGGAAAGTCC
ATGATGAGCTCCGTGAGCCTGATGGGGGGCCGGGGCGGGGTGCCCCTCTACGACCGGAACCACGTCACAGG
GGCCTCGTCCAGCAGCTCGTCCAGCACGAAGGCCACGCTGTACCCGCCGATCCTGAACCCGCCGCCCTCCCCG

GCCACGGACCCCTCCCTGTACAACATGGACATGTTCTACTCTTCAAACATTCCGGCCACTGCGAGACCGTACAG
GCCCTACATCATTCGAGGAATGGCGCCCCCGACGACGCCCTGCAGCACCGACGTGTGTGACAGCGACTACAG
CGCCAGCCGCTGGAAGGCCAGCAAGTACTACCTGGATTTGAACTCGGACTCAGACCCCTATCCACCCCCACCC
ACGCCCCACAGCCAGTACCTGTCGGCGGAGGACAGCTGCCCGCCCTCGCCCGCCACCGAGAGGAGCTACTTC
CATCTCTTCCCGCCCCCTCCGTCCCCCTGCACGGACTCATCC**TGA**

Supplementary Table S4: Primer sequences used for cloning

S.No.	Primer ID	Name	Direction	Restriction enzyme	Primer sequence
1	OAD1571	*LRP5-UBE3C* (v1)	F	SnaBI	CCGTACGTAATGGAGGCAGCGCCGCC
	OAD1572	*LRP5-UBE3C* (v1)	R	EcoRI	GGCGAATTCTCAGCTCAGCTCAAAGCCAGC
2	OAD2274	*UBE3C-LRP5* (v1)	F	SnaBI	CCGTACGTAATGCGGGATACAACTC
	OAD2149	*UBE3C-LRP5* (v1)	R	EcoRI	GGCGAATTCTCAGGATGAGTCCGTGCAGG
3	OAD2339	*UBE3C-LRP5* (v2)	F	SnaBI	CCGTACGTAATGGGCCTCATGTACTG
	OAD2149	*UBE3C-LRP5* (v2)	R	EcoRI	GGCGAATTCTCAGGATGAGTCCGTGCAGG
4	OAD2368	*UBE3C-LRP5* (v7)	F	SnaBI	CCGTACGTAATGTGTGAAATCACCAAGCC
	OAD2149	*UBE3C-LRP5* (v7)	R	EcoRI	GGCGAATTCTCAGGATGAGTCCGTGCAGG

Supplementary Table S5: Primer sequences used for siRNA-mediated knockdown of fusion transcript variants, *LRP5* , and Wnt pathway genes

Primer ID	Primer sequence
OAD3105_T7 promoter_FP	TAATACGACTCACTATAG
OAD2892_scrambled as-siRNA	AAGAAATGCATGCATGAGAAGCTATAGTGAGTCGTATTA
OAD2893_scrambled s-siRNA	CAGCTTCTCAATGCATGCCTTCTATAGTGAGTCGTATTA
OAD3095_*UBE3C-LRP5* (v1)_as-siRNA	CAACTCCTGTCTCCACAGGAGCTATAGTGAGTCGTATTA
OAD3096_*UBE3C-LRP5* (v1)_s-siRNA	CGGCTCCTGTGGAGACAGGAGCTATAGTGAGTCGTATTA
OAD3097_*UBE3C-LRP5* (v2)_as-siRNA	GGGATACAACTCCTGGACGGGCTATAGTGAGTCGTATTA
OAD3098_*UBE3C-LRP5* (v2)_s-siRNA	AGACCCGTCCAGGAGTTGTATCTATAGTGAGTCGTATTA
OAD3103_*UBE3C-LRP5* (v7)_as-siRNA-1	ATCGCATGCGGAAAGTCCATGCTATAGTGAGTCGTATTA
OAD3104_*UBE3C-LRP5* (v7)_s-siRNA-1	CATCATGGACTTTCCGCATGCCTATAGTGAGTCGTATTA
OAD3108_*UBE3C-LRP5* (v7)_as-siRNA-2	ATCATTCGAGGAATGGCGCCCCTATAGTGAGTCGTATTA
OAD3109_*UBE3C-LRP5* (v7)_s-siRNA-2	CGGGGGCGCCATTCCTCGAATCTATAGTGAGTCGTATTA
OAD3099_*LRP5* _as-siRNA-1	CAACCACATCTACTGGACAGACTATAGTGAGTCGTATTA
OAD3100_*LRP5* _s-siRNA-1	ACGTCTGTCCAGTAGATGTGGCTATAGTGAGTCGTATTA
OAD3101_*LRP5* _as-siRNA-2	AAGCGAGCCTTTCTACACATGCTATAGTGAGTCGTATTA
OAD3102_*LRP5* _s-siRNA-2	GCGCATGTGTAGAAAGGCTCGCTATAGTGAGTCGTATTA
OAD3131_*CTNNB1* _as-siRNA-1	CAGCGTTTGGCTGAACCATCACTATAGTGAGTCGTATTA
OAD3132_*CTNNB1* _s-siRNA-1	CTGTGATGGTTCAGCCAAACGCTATAGTGAGTCGTATTA
OAD3133_*CTNNB1* _as-siRNA-2	TATAATGAGGACCTATACTTACTATAGTGAGTCGTATTA
OAD3134_*CTNNB1* _s-siRNA-2	TCGTAAGTATAGGTCCTCATTCTATAGTGAGTCGTATTA
OAD2872_*LEF1* _as-siRNA-1	GTCCTCCTGGTCCCCACACAACTATAGTGAGTCGTATTA
OAD2873_*LEF1* _s-siRNA-1	CAGTTGTGTGGGGACCAGGAGCTATAGTGAGTCGTATTA
OAD2874_*LEF1* _as-siRNA-2	GTGAAGAGCAGGCTAAATATTCTATAGTGAGTCGTATTA
OAD2875_*LEF1* _s-siRNA-2	CATAATATTTAGCCTGCTCTTCTATAGTGAGTCGTATTA

Supplementary Table S6: List of 242 fusion transcripts identified in the transcriptome sequencing data of tongue tumors and cell lines

5pGene-3pGene	5pChr_endposition_3pChr_startposition	Type	Number of samples	Sample_info
CLN6-CALML4	chr15_68522079_chr15_68483042	Read_Through	7	23T;OT9;NT8e;9T;AW8507;AW13516;33T
DKFZp451F083-ZNF75D	chrX_134477956_chrX_134419722	Overlapping_Complex	7	19T;23T;OT9;11T;AW8507;AW13516;33T
FAM208B-C10orf18	chr10_5727137_chr10_5751492	Overlapping_Same	6	38T;NT8e;9T;11T;AW8507;AW13516
KIAA0889-CCDC165	chr20_35492086_chr18_8718421	Interchromosomal	6	23T;NT8e;9T;AW8507;AW13516;33T
KIAA1267-ARL17A	chr17_44270165_chr17_44630121	Intrachromosomal_Complex	6	10T;19T;23T;9T;15T;33T
BACH1-GRIK1-AS2	chr21_30702013_chr21_30969889	Read_Through	5	29T;23T;9T;AW8507;33T
MATL2963-CLSTN3	chr12_7273220_chr12_7285619	Read_Through	5	23T;9T;AW8507;15T;33T
POLA2-CDC42EP2	chr11_65063460_chr11_65088014	Read_Through	5	38T;10T;AW8507;AW13516;33T
TLK2-AL137655	chr17_60637486_chr11_141452	Interchromosomal	5	38T;19T;11T;AW8507;33T
KLF16-REXO1	chr19_1863563_chr19_1815244	Read_Through	4	23T;OT9;9T;AW8507
MFSD7-ATP5I	chr4_682972_chr4_666224	Read_Through	4	38T;19T;23T;9T
RRM2-C2orf48	chr2_10269280_chr2_10281980	Read_Through	4	23T;AW8507;AW13516;33T
TMEM141-KIAA1984	chr9_139686809_chr9_139693553	Read_Through	4	10T;19T;AW13516;33T
TPD52L2-DNAJC5	chr20_62514172_chr20_62559687	Read_Through	4	23T;11T;AW13516;33T
VMAC-CAPS	chr19_5905091_chr19_5914395	Read_Through	4	19T;23T;OT9;9T
CTSD-IFITM10	chr11_1785221_chr11_1753639	Read_Through	3	OT9;AW13516;39T
IGSF3-GGT1	chr1_117210313_chr22_25003920	Interchromosomal	3	NT8e;AW8507;AW13516
KLK8-KLK7	chr19_51504964_chr19_51479734	Read_Through	3	38T;11T;AW8507
LMAN2-MXD3	chr5_176778884_chr5_176733817	Read_Through	3	OT9;AW13516;33T
MED22-SURF6	chr9_136214971_chr9_136197551	Read_Through	3	23T;OT9;9T
MFGE8-HAPLN3	chr15_89455754_chr15_89420518	Read_Through	3	10T;AW13516;15T
MXD3-RAB24	chr5_176739291_chr5_176728198	Read_Through	3	29T;19T;23T
NUDT14-JAG2	chr14_105647659_chr14_105608075	Read_Through	3	AW8507;AW13516;15T
PMS2L2-CCDC146	chr7_72511868_chr7_76796974	Intrachromosomal	3	23T;AW13516;33T
PMS2P5-CCDC146	chr7_74313950_chr7_76796974	Intrachromosomal	3	23T;AW13516;33T
PRIM1-NACA	chr12_57146145_chr12_57106210	Read_Through	3	OT9;AW8507;33T
RRN3P3-CDR2	chr16_22449035_chr16_22357256	Read_Through	3	23T;OT9;9T
SLC39A1-CRTC2	chr1_153935797_chr1_153920147	Read_Through	3	9T;15T;33T
SMG1-ARL6IP1	chr16_18937725_chr16_18802990	Read_Through	3	10T;11T;AW13516
ZDHHC1-TPPP3	chr16_67450338_chr16_67423711	Read_Through	3	23T;OT9;9T
ARHGAP11A-SCG5	chr15_32928115_chr15_32935786	Read_Through	2	AW8507;AW13516
C10orf18-FAM208B	chr10_5751625_chr10_5754785	Overlapping_Complex	2	AW8507;AW13516
CAGE1-SSR1	chr6_7374363_chr6_7281287	Read_Through	2	AW13516;15T
CTSC-RAB38	chr11_88070940_chr11_87846414	Read_Through	2	19T;33T
CYB5R1-ADIPOR1	chr1_202936403_chr1_202909960	Read_Through	2	11T;33T
DUS3L-PRR22	chr19_5791248_chr19_5782970	Read_Through	2	38T;33T
ENTPD7-CUTC	chr10_101462429_chr10_101496002	Read_Through	2	9T;AW8507
EXT1-MED30	chr8_119124057_chr8_118552121	Adjacent_Converging	2	AW8507;AW13516
FBXO46-SNRPD2	chr19_46234150_chr19_46190711	Read_Through	2	23T;9T
GBP3-RBMXL1	chr1_89488548_chr1_89445138	Read_Through	2	AW8507;AW13516
GFOD2-C16orf48	chr16_67753272_chr16_67696849	Read_Through	2	9T;AW13516
HP11097-CCDC33	chr15_74501874_chr15_74536325	Read_Through	2	AW8507;AW13516
KLHL22-SCARF2	chr22_20850169_chr22_20778873	Read_Through	2	23T;AW13516
MBD1-CCDC11	chr18_47808136_chr18_47753562	Read_Through	2	AW8507;33T
MBD1-CCDC11	chr18_47803367_chr18_47753562	Read_Through	2	9T;15T
MBD1-CCDC11	chr18_47808143_chr18_47753562	Read_Through	2	11T;AW13516
MFGE8-HAPLN3	chr15_89456662_chr15_89420518	Read_Through	2	AW8507;33T
RIPK3-ADCY4	chr14_24808270_chr14_24787554	Read_Through	2	19T;11T
SNTB2-VPS4A	chr16_69333676_chr16_69349910	Read_Through	2	9T;AW8507
SYT7-SYT7	chr11_61309869_chr11_61281187	Overlapping_Same	2	23T;15T
TBC1D23-NIT2	chr3_100039814_chr3_100057930	Read_Through	2	11T;33T
TSC22D4-C7orf61	chr7_100076901_chr7_100054237	Read_Through	2	19T;9T
VPS72-TMOD4	chr1_151162639_chr1_151142462	Read_Through	2	19T;23T
ZNF264-AURKC	chr19_57716859_chr19_57743109	Read_Through	2	AW8507;AW13516
ZNF580-ZNF581	chr19_56152548_chr19_56155918	Overlapping_Complex	2	AW13516;15T
ZNF628-NAT14	chr19_55988173_chr19_55997027	Read_Through	2	19T;9T
ABHD14B-PCBP4	chr3_52008645_chr3_51991469	Read_Through	1	OT9
ABHD14B-PCBP4	chr3_52017424_chr3_51991469	Read_Through	1	11T
ADORA2B-TTC19	chr17_15848896_chr17_15907514	Read_Through	1	AW13516
AF086285-ATP6V1E2	chr2_46769140_chr2_46738985	Read_Through	1	AW13516
AGAP4-FRMPD2	chr10_46342920_chr10_49364600	Intrachromosomal_Complex	1	AW8507
AGAP5-FRMPD2	chr10_75490271_chr10_49364600	Intrachromosomal	1	AW8507
AGAP8-FRMPD2	chr10_51371330_chr10_49364600	Intrachromosomal	1	AW8507
AIM1L-UBXN11	chr1_26680620_chr1_26608772	Read_Through	1	10T
ALG14-CNN3	chr1_95538506_chr1_95362506	Read_Through	1	AW8507
AQP7-AQP7P3	chr9_33402516_chr9_42883123	Intrachromsomal_Diverging	1	19T
ARHGAP1-ARHGAP8	chr11_46722119_chr22_45210551	Interchromosomal	1	33T
ARID3C-DCTN3	chr9_34628010_chr9_34613547	Read_Through	1	OT9
ARL6IP1-RPS15A	chr16_18812856_chr16_18794276	Read_Through	1	38T
ARMC6-SLC25A42	chr19_19168466_chr19_19206899	Read_Through	1	19T
ATP6V0C-AMDHD2	chr16_2564182_chr16_2570984	Read_Through	1	NT8e
AX747182-POLR2J2	chr7_44047464_chr7_102277194	Intrachromosomal_Complex	1	23T

BCAR3-MIG7	chr1_94312705_chr1_94219111	Overlapping_Complex	1	AW13516
BID-MICAL3	chr22_18257260_chr22_18270415	Adjacent_Complex	1	AW13516
BMS1-AQP7P1	chr10_43319231_chr9_67254266	Interchromosomal	1	19T
BPTF-AMZ2	chr17_65822452_chr17_66246328	Intrachromosomal	1	AW8507
BTN2A3P-BTN3A2	chr6_26429012_chr6_26368217	Intrachromosomal_Complex	1	9T
BTN2A3P-BTN3A3	chr6_26429012_chr6_26443609	Read_Through	1	9T
BX247991-EFCAB11	chr14_90303006_chr14_90263468	Overlapping_Same	1	AW13516
BX648270-C2orf27A	chr2_132445875_chr2_132508205	Read_Through	1	23T
C14orf149-GPR135	chr14_59951072_chr14_59895739	Read_Through	1	AW13516
C15orf26-IL16	chr15_81430475_chr15_81517639	Read_Through	1	AW8507
C17orf99-SYNGR2	chr17_76160444_chr17_76166897	Read_Through	1	38T
C19orf79-STXBP2	chr19_7695731_chr19_7703611	Read_Through	1	OT9
C20orf29-MAVS	chr20_3802939_chr20_3835204	Read_Through	1	33T
CCDC159-LPPR2	chr19_11465371_chr19_11467477	Read_Through	1	AW8507
CCDC19-VSIG8	chr1_159869905_chr1_159824105	Read_Through	1	10T
CDCA5-AB231703	chr11_64851614_chr11_64834080	Read_Through	1	OT9
CDSN-C6orf15	chr6_31088251_chr6_31078999	Read_Through	1	10T
CECR7-IL17RA	chr22_17528315_chr22_17577951	Read_Through	1	AW13516
CELF1-RAPSN	chr11_47574791_chr11_47459307	Read_Through	1	23T
CHD4-NOP2	chr12_6715616_chr12_6666036	Read_Through	1	AW13516
CIRBP-C19orf24	chr19_1274438_chr19_1277181	Read_Through	1	10T
CLEC7A-CLEC1A	chr12_10282695_chr12_10223079	Read_Through	1	9T
CLIP1-VPS33A	chr12_122907115_chr12_122692208	Read_Through	1	23T
CLTC-VMP1	chr17_57754548_chr17_57915655	Read_Through	1	15T
CNPY2-CS	chr12_56710127_chr12_56665482	Read_Through	1	AW8507
CRIP2-CRIP1	chr14_105945829_chr14_105954502	Read_Through	1	AW13516
CRY2-MAPK8IP1	chr11_45893783_chr11_45919635	Read_Through	1	10T
CSNK1E-ZNF217	chr22_38794526_chr20_52183609	Interchromosomal	1	AW8507
CTNNBIP1-CLSTN1	chr1_9970315_chr1_9789078	Read_Through	1	AW13516
CYP4B1-CYP4X1	chr1_47280935_chr1_47495661	Read_Through	1	19T
DHRS13-FLOT2	chr17_27230088_chr17_27206356	Read_Through	1	AW8507
DMWD-DMPK	chr19_46296059_chr19_46272975	Read_Through	1	AW13516
DSTYK-LRRN2	chr1_205180726_chr1_204586302	Intrachromosomal	1	23T
DTX3-CCT2	chr12_58000300_chr12_69993642	Intrachromosomal	1	19T
DUOXA1-DUOX2	chr15_45422056_chr15_45384851	Read_Through	1	10T
EP400NL-EP400NL	chr12_132593226_chr12_132604964	Read_Through	1	10T
F3-SPG7	chr1_95007412_chr16_89611055	Interchromosomal	1	AW13516
FAM108C1-KIAA1199	chr15_80988359_chr15_81165878	Read_Through	1	AW8507
FAM108C1-KIAA1199	chr15_80988359_chr15_81166204	Read_Through	1	AW13516
FAM208B-C10orf18	chr10_5727137_chr10_5754356	Overlapping_Same	1	OT9
FLNC-ATP6V1F	chr7_128498270_chr7_128505430	Read_Through	1	9T
FTSJD2-BTBD9	chr6_37411925_chr6_38136226	Intrachromosomal_Converging	1	OT9
GLRX2-UCHL5	chr1_193074607_chr1_192981495	Read_Through	1	AW8507
GUSBP1-GTF2H2	chr5_21342084_chr5_70330950	Intrachromosomal_Converging	1	33T
GUSBP1-GTF2H2B	chr5_21342084_chr5_69716075	Intrachromosomal	1	33T
GUSBP2-GTF2H2	chr6_26924332_chr5_70330950	Interchromosomal	1	33T
GUSBP2-GTF2H2B	chr6_26924332_chr5_69716075	Interchromosomal	1	33T
HCP5-MICB	chr6_31431055_chr6_31473393	Intrachromosomal	1	23T
HDAC8-CITED1	chrX_71787879_chrX_71521487	Read_Through	1	23T
HERC2-HERC2P3	chr15_28567294_chr15_20588367	Intrachromosomal	1	AW8507
HILPDA-EFCAB3	chr7_128096076_chr17_60491092	Interchromosomal	1	AW8507
HOXB6-HOXB3	chr17_46682353_chr17_46626231	Intrachromosomal	1	AW8507
HOXC10-HOXC4	chr12_54379793_chr12_54446943	Intrachromosomal	1	AW8507
HSF4-NOL3	chr16_67203003_chr16_67208064	Read_Through	1	15T
IGSF3-CD58	chr1_117210313_chr1_117057155	Read_Through	1	NT8e
IL17RC-CRELD1	chr3_9974386_chr3_9976518	Read_Through	1	10T
IL17RC-CRELD1	chr3_9974386_chr3_9976103	Read_Through	1	OT9
IL18R1-ZNF664	chr2_102990452_chr12_124495927	Interchromosomal	1	AW8507
IL1F10-IL1RN	chr2_113832426_chr2_113868692	Read_Through	1	23T
IL2RG-CXorf65	chrX_70329239_chrX_70323738	Read_Through	1	33T
ING5-ATG4B	chr2_242644138_chr2_242606059	Intrachromosomal_Complex	1	AW8507
INPP5K-MYO1C	chr17_1420181_chr17_1367479	Read_Through	1	AW8507
KIAA1267-ARL17A	chr17_44302739_chr17_44630121	Intrachromosomal_Complex	1	AW8507
KLHDC4-FLJ00104	chr16_87743272_chr16_87731753	Read_Through	1	AW13516
LAIR1-LAIR2	chr19_54876720_chr19_55021735	Intrachromsomal_Diverging	1	9T
LARS2-LIMD1	chr3_45565599_chr3_45677641	Read_Through	1	AW13516
LRP4-CKAP5	chr11_46940172_chr11_46765083	Intrachromosomal	1	AW13516
LRP5-UBE3C	chr11_68133169_chr7_157060278	Interchromosomal	1	NT8e
LRRFIP1-RBM44	chr2_238683104_chr2_238722234	Read_Through	1	AW8507
LYPLA1-TCEA1	chr8_55014576_chr8_54879115	Intrachromosomal	1	9T
MAEA-CTBP1	chr4_1283769_chr4_1205227	Intrachromsomal_Diverging	1	AW8507
MAP3K11-KCNK7	chr11_65378542_chr11_65360325	Read_Through	1	33T
MAPKAPK5-ALDH2	chr12_112308983_chr12_112247346	Intrachromosomal_Complex	1	AW8507
MBD1-CCDC11	chr18_47807676_chr18_47753562	Read_Through	1	23T
MED12-NLGN3	chrX_70361813_chrX_70367399	Read_Through	1	AW8507

MGC57346-C17orf69	chr17_43707523_chr17_43723266	Read_Through	1	29T
MIG7-BCAR3	chr1_94240929_chr1_94027348	Overlapping_Same	1	AW13516
MIR22HG-TLCD2	chr17_1619565_chr17_1606083	Read_Through	1	9T
NAALADL2-NAALADL2	chr3_174455535_chr3_174814579	Read_Through	1	19T
NACC2-QSOX2	chr9_138987130_chr9_139098181	Intrachromosomal_Complex	1	AW8507
NAIP-GTF2H2	chr5_70282272_chr5_70330950	Intrachromosomal_Complex	1	10T
NAIP-GTF2H2B	chr5_69407509_chr5_69716075	Intrachromsomal_Diverging	1	10T
NAIP-GTF2H2C	chr5_70282272_chr5_68860926	Intrachromosomal_Converging	1	10T
NBPF24-NBPF15	chr1_146068251_chr1_148574736	Intrachromsomal_Diverging	1	AW8507
NBPF24-NBPF15	chr1_147610087_chr1_148574736	Intrachromsomal_Diverging	1	AW8507
NCKIPSD-CELSR3	chr3_48723333_chr3_48662830	Read_Through	1	AW8507
NCSTN-NHLH1	chr1_160324079_chr1_160340346	Read_Through	1	AW8507
NDUFV3-PKNOX1	chr21_44317156_chr21_44424435	Read_Through	1	23T
NF1-AK4	chr17_29670152_chr1_65613960	Interchromosomal	1	23T
NHP2L1-LLPH	chr22_42084912_chr12_66516848	Interchromosomal	1	NT8e
NSUN4-FAAH	chr1_46826499_chr1_46867762	Read_Through	1	9T
NXN-GLOD4	chr17_882997_chr17_662548	Read_Through	1	9T
OGT-TAF1	chrX_70787965_chrX_70748391	Adjacent_Complex	1	AW8507
OMA1-AX746780	chr1_59012445_chr1_58933598	Read_Through	1	23T
OSBPL8-NUP107	chr12_76953588_chr12_69094505	Intrachromosomal_Converging	1	19T
OSBPL8-TSPAN8	chr12_76953588_chr12_71518876	Intrachromosomal	1	19T
PARG-BMS1	chr10_51371330_chr10_43287074	Intrachromosomal_Converging	1	23T
PBX1-AX748175	chr1_164532547_chr1_164651546	Overlapping_Same	1	19T
PC-C11orf80	chr11_66675339_chr11_66605837	Adjacent_Converging	1	OT9
PDLIM2-C8orf58	chr8_22447253_chr8_22458394	Read_Through	1	23T
PEX26-TUBA8	chr22_18562779_chr22_18604245	Read_Through	1	23T
PEX26-TUBA8	chr22_18568023_chr22_18606922	Read_Through	1	AW8507
PEX26-TUBA8	chr22_18566497_chr22_18606922	Read_Through	1	33T
PEX26-TUBA8	chr22_18568023_chr22_18604245	Read_Through	1	9T
PFKFB4-SHISA5	chr3_48594226_chr3_48509196	Read_Through	1	9T
PHACTR4-SESN2	chr1_28733973_chr1_28605607	Intrachromosomal_Complex	1	NT8e
PILRB-PILRB	chr7_99947509_chr7_99949884	Read_Through	1	AW13516
PLAUR-CADM4	chr19_44174497_chr19_44126521	Read_Through	1	AW13516
PLEKHO2-ANKDD1A	chr15_65153773_chr15_65214119	Read_Through	1	9T
PLEKHO2-ANKDD1A	chr15_65147235_chr15_65218264	Read_Through	1	AW8507
PMS2CL-POM121	chr7_6771585_chr7_72361165	Intrachromosomal	1	AW13516
PMS2P3-CCDC146	chr7_75157452_chr7_76796974	Intrachromsomal_Diverging	1	23T
PMS2P3-RASA4	chr7_75157452_chr7_102230605	Intrachromosomal_Complex	1	23T
PMS2P5-RASA4	chr7_74313950_chr7_102230605	Intrachromosomal	1	AW13516
PPIP5K1-CATSPER2	chr15_43882450_chr15_43922771	Intrachromosomal_Complex	1	9T
PRKCH-FLJ22447	chr14_61997312_chr14_62117679	Read_Through	1	23T
PRRC2B-METTL11A	chr9_134334742_chr9_132394928	Intrachromosomal_Complex	1	39T
PRX-HIPK4	chr19_40910194_chr19_40885177	Read_Through	1	33T
PSMD5-VAV2	chr9_123605205_chr9_136627015	Intrachromosomal_Complex	1	9T
QSOX2-EGFL7	chr9_139137686_chr9_139563008	Intrachromsomal_Diverging	1	23T
RAD18-OXTR	chr3_9005158_chr3_8792094	Read_Through	1	AW8507
RNASET2-RPS6KA2	chr6_167370076_chr6_166822853	Read_Through	1	29T
RNF7-GRK7	chr3_141457357_chr3_141499215	Read_Through	1	AW8507
RPL23AP53-41883	chr8_182317_chr7_55861236	Interchromosomal	1	AW8507
RPL23AP53-AL137655	chr8_182317_chr11_120810	Interchromosomal	1	AW8507
RPL23AP53-AL137655	chr8_182317_chr3_197955064	Interchromosomal	1	AW8507
RPL23AP82-MGC2752	chr22_51223720_chr19_59102864	Interchromosomal	1	9T
SAR1A-TYSND1	chr10_71930284_chr10_71897732	Read_Through	1	AW8507
SAV1-GYPE	chr14_51135022_chr4_144792018	Interchromosomal	1	23T
SCNN1A-TNFRSF1A	chr12_6473312_chr12_6437922	Read_Through	1	AW13516
SDF4-TNFRSF4	chr1_1167446_chr1_1146705	Read_Through	1	NT8e
SHANK3-ACR	chr22_51160864_chr22_51177698	Read_Through	1	19T
SIRPB2-NSFL1C	chr20_1472232_chr20_1422806	Read_Through	1	AW13516
SLC35A3-HIAT1	chr1_100483370_chr1_100515464	Read_Through	1	AW8507
SLC35B1-SPOP	chr17_47785281_chr17_47676245	Read_Through	1	33T
SLC35E2-CDC2L1	chr1_1677437_chr1_1586822	Intrachromosomal	1	33T
SMA4-GTF2H2	chr5_69140739_chr5_70330950	Intrachromosomal_Converging	1	33T
SMA4-GTF2H2B	chr5_69140739_chr5_69716075	Intrachromosomal	1	33T
SNRNP200-NCAPH	chr2_96971306_chr2_97017568	Intrachromsomal_Diverging	1	NT8e
ST6GALNAC4-ST6GALNAC6	chr9_130679304_chr9_130647600	Read_Through	1	AW8507
ST7-ST7-OT4	chr7_116593744_chr7_116595027	Overlapping_Same	1	19T
STX12-PPP1R8	chr1_28146242_chr1_28159266	Read_Through	1	11T
TBC1D15-PPHLN1	chr12_72291720_chr12_42729684	Intrachromosomal_Complex	1	19T
TBC1D22B-RNF8	chr6_37284981_chr6_37328221	Read_Through	1	29T
TCRBV5S1A1T-TCRVB	chr7_142021361_chr7_142494245	Intrachromosomal	1	9T
TCRBV7S1A1N2T-TCRVB	chr7_142013486_chr7_142495140	Intrachromosomal	1	23T
THAP5-PNPLA8	chr7_108210166_chr7_108112070	Read_Through	1	11T
TMEM111-PRRT3	chr3_10028521_chr3_9987225	Read_Through	1	AW8507
TMEM205-RAB3D	chr19_11456980_chr19_11432721	Read_Through	1	23T
TMLHE-CLIC2	chrX_154842621_chrX_154505499	Intrachromosomal	1	19T

TNNI1-LAD1	chr1_201386920_chr1_201353371	Read_Through	1	9T
TOPORS-DDX58	chr9_32552625_chr9_32455299	Read_Through	1	19T
TRADD-B3GNT9	chr16_67193811_chr16_67182004	Read_Through	1	AW13516
TTC27-BIRC6	chr2_32865476_chr2_32842791	Adjacent_Complex	1	AW8507
TYSND1-AIFM2	chr10_71906495_chr10_71872022	Read_Through	1	19T
UBAC1-CAMSAP1	chr9_138853225_chr9_138700332	Read_Through	1	AW8507
UBE2D2-CXXC5	chr5_139003045_chr5_139059948	Read_Through	1	19T
UBE2L3-CCDC116	chr22_21965331_chr22_21990720	Read_Through	1	AW13516
UBR2-CACNA2D4	chr6_42585244_chr12_1904833	Interchromosomal	1	AW8507
UGCG-ODF2	chr9_114688775_chr9_131260691	Intrachromosomal	1	39T
VAMP8-VAMP5	chr2_85806289_chr2_85818847	Read_Through	1	AW13516
VPS13B-RNF139	chr8_100287481_chr8_125498071	Intrachromosomal	1	NT8e
VPS33B-PRC1	chr15_91565832_chr15_91509267	Read_Through	1	33T
WRB-SH3BGR	chr21_40763761_chr21_40834300	Read_Through	1	19T
WWTR1-PGAP1	chr3_149375108_chr2_197697727	Interchromosomal	1	19T
XAF1-FBXO39	chr17_6674302_chr17_6690098	Read_Through	1	19T
XKR8-RNF216P1	chr1_28290203_chr7_5035105	Interchromosomal	1	33T
YIF1A-RCOR2	chr11_66056637_chr11_63678692	Intrachromosomal	1	OT9
ZNF782-ZNF510	chr9_99637855_chr9_99518146	Read_Through	1	33T
ZNF841-ZNF432	chr19_52599017_chr19_52536676	Read_Through	1	11T

Supplementary Table S7: List of fusion transcript pairs overlapping with the fusion databases

S.No.	Fusion	FusionCancer	TCGA-PanCancer	TCGA-HNSC	Number of samples
1	*CLTC-VMP1*	■		■	1
2	*PMS2P5-CCDC146*			■	3
3	*PMS2P5-RASA4*		■		1
4	*SAV1-GYPE*		■		1
5	*SMA4-GTF2H2*		■		1
6	*BMS1-AQP7P1*	■			1
7	*BPTF-AMZ2*	■			1
8	*C15orf26-IL16*	■			1
9	*C17orf99-SYNGR2*	■			1
10	*CECR7-IL17RA*	■			1
11	*CIRBP-C19orf24*	■			1
12	***CLN6-CALML4***	■			7
13	*CNPY2-CS*	■			1
14	*CRIP2-CRIP1*	■			1
15	***CTSC-RAB38***	■			2
16	*CTSD-IFITM10*	■			3
17	*HERC2-HERC2P3*	■			1
18	*HOXB6-HOXB3*	■			1
19	*IGSF3-GGT1*	■			3
20	*IL17RC-CRELD1*	■			2
21	*KIAA0889-CCDC165*	■			6
22	*KIAA1267-ARL17A*	■			7
23	*LMAN2-MXD3*	■			3
24	*MAEA-CTBP1*	■			1
25	*MAPKAPK5-ALDH2*	■			1
26	*MBD1-CCDC11*	■			7
27	*NBPF24-NBPF15*	■			1
28	*NCKIPSD-CELSR3*	■			1
29	*NF1-AK4*	■			1
30	*NHP2L1-LLPH*	■			1
31	*NSUN4-FAAH*	■			1
32	*OSBPL8-TSPAN8*	■			1
33	*PLEKHO2-ANKDD1A*	■			1
34	***POLA2-CDC42EP2***	■			5
35	*PRIM1-NACA*	■			3
36	***RRM2-C2orf48***	■			4
37	*RRN3P3-CDR2*	■			3
38	*SAR1A-TYSND1*	■			1
39	*SCNN1A-TNFRSF1A*	■			1
40	*SIRPB2-NSFL1C*	■			1
41	*SLC35A3-HIAT1*	■			1
42	*TBC1D23-NIT2*	■			2
43	*TCRBV5S1A1T-TCRVB*	■			1
44	*TLK2-AL137655*	■			5
45	*TMEM141-KIAA1984*	■			4
46	*TPD52L2-DNAJC5*	■			4
47	*WRB-SH3BGR*	■			1

Bold highlighted fusion transcripts were also validated by Sanger sequencing

Supplementary Table S8: Details of the 12 validated fusion transcripts

S.No.	Fusion Name	In-frame	Type	Gene	Distance between fused exons (kb)	Cytoband	Exon No.	Sub-cellular location of Protein	Domain	Amino Acid included in fusion
1	*LRP5-UBE3C*	No	Inter-chromosomal	*LRP5*	Chr11-Chr7	11q13	5	extra cellular	EGF-1 repeat	1-330
				UBE3C		7q36	23	active site	HECT domian	1028-1084
2	*YIF1A-RCOR2*	Yes	Intra-chromosomal	*YIF1A*	2377.94	11q13	1	Cyotplasma	NA	1-11
				RCOR2		11q13	6	Cytoplasma	SANT domain	161-524
3	*EXT1-MED30*	Yes	Intra-chromosomal	*EXT1*	571.93	8q24	1	ER and Golgi	Topological domain	1-321
				MED30		8q24	4	Nucleus	Coiled-coil domain	148-178
4	*RRM2-C2orf48*	No	Read Through	*RRM2*	12.70	2q25	9	Cyotplasma	Helix	1-399
				C2orf48		2q25	1	NA	NA	NA
5	*CLN6-CALML4*	No	Read Through	*CLN6*	39.04	15q23	2	Plasma membrame	Transmembrane domian	1-66
				CALML4		15q23	4	NA	EF-HAND-2	102-196
6	*BACH1-GRIK1*	No	Read Through	*BACH1*	267.87	21q21	4	Nucleus	b ZIP	1-592
				GRIK1		21q21	2	Extracellular	Intron	402-918
7	*CTSC-RAB38*	Yes	Read Through	*CTSC*	224.52	11q14	5	Lysosomes	Dipeptidyl peptidase	1-253
				RAB38		11q14	2	Plasma membrame	Nucleotide phosphate-binding region	68-211
8	*POLA2-CDC42EP2*	No	Read Through	*POLA2*	24.55	11q13	16	Nucleus	b-Strand	1-549
				CDC42EP2		11q13	2	NA	Non-coding	NA
9	*SLC39A1-CRTC2*	Yes	Read Through	*SLC39A*	15.65	1q21	3	Plasma membrame	Topological domain	1-106
				CRTC2		1q21	2	Cyto/nucleus	CREB-regulated transcription coactivator 2	52-693
10	*PSMD5-VAV2*	Yes	Intra-chromosomal	*PSMD5*	13021.80	9q33	9	cytosol	NA	1-419
				VAV2		9q34	26	cytosol	SH2 domian	712-878
11	*FTSJD2-BTBD9*	Yes	Intra-chromosomal	*FTSJD2*	724.30	6p21	3	Nucleus	G-patch	1-95
				BTBD9		6p21	9	Cyotplasma	NA	455-610
12	*NAIP-GTF2H2B*	Yes	Intra-chromosomal	*NAIP*	308.56	5q13	2	Plasma membrame	ATP binding domain	1-760
				GTF2H2B		5q13	2	Nucleus	Full protein	1-395

Supplementary Table S9: Patient sample cohorts used for identification and validation of fusion transcripts

S.No.	Sample ID	Patient sample set	*LRP5-UBE3C* fusion	*UBE3C-LRP5* fusion
1	AD0486	Validation set 1	■	■
2	AD0495	Validation set 1	■	■
3	AD0501	Discovery set and validation set 1	■	
4	AD0496	Validation set 1	■	
5	AD0503	Validation set 1	■	
6	AD0514	Validation set 1	■	
7	AD3067	Validation set 2		■
8	AD3079	Validation set 2		■
9	AD0487	Discovery set and validation set 1		
10	AD0488	Discovery set and validation set 1		
11	AD0489	Discovery set and validation set 1		
12	AD0493	Discovery set and validation set 1		
13	AD0497	Discovery set and validation set 1		
14	AD0507	Discovery set and validation set 1		
15	AD0511	Discovery set and validation set 1		
16	AD0516	Discovery set and validation set 1		
17	AD0517	Discovery set and validation set 1		
18	AD0479	Validation set 1		
19	AD0480	Validation set 1		
20	AD0481	Validation set 1		
21	AD0483	Validation set 1		
22	AD0485	Validation set 1		
23	AD0505	Validation set 1		
24	AD0508	Validation set 1		
25	AD0509	Validation set 1		
26	AD0510	Validation set 1		
27	AD0512	Validation set 1		
28	AD0515	Validation set 1		
29	AD0518	Validation set 1		
30	AD0519	Validation set 1		
31	AD0520	Validation set 1		
32	AD0521	Validation set 1		
33	AD0522	Validation set 1		
34	AD0523	Validation set 1		
35	AD0524	Validation set 1		
36	AD0525	Validation set 1		
37	AD0526	Validation set 1		
38	AD0527	Validation set 1		
39	AD0536	Validation set 1		
40	AD0537	Validation set 1		
41	AD0538	Validation set 1		
42	AD0539	Validation set 1		
43	AD0540	Validation set 1		
44	AD0541	Validation set 1		

45	AD0542	Validation set 1		
46	AD0543	Validation set 1		
47	AD0482	Validation set 2		
48	AD0484	Validation set 2		
49	AD0491	Validation set 2		
50	AD0492	Validation set 2		
51	AD0494	Validation set 2		
52	AD0498	Validation set 2		
53	AD0499	Validation set 2		
54	AD0500	Validation set 2		
55	AD0502	Validation set 2		
56	AD0504	Validation set 2		
57	AD0506	Validation set 2		
58	AD0513	Validation set 2		
59	AD0544	Validation set 2		
60	AD2886	Validation set 2		
61	AD3045	Validation set 2		
62	AD3047	Validation set 2		
63	AD3553	Validation set 2		
64	AD2893	Validation set 2		
65	AD3554	Validation set 2		
66	AD3049	Validation set 2		
67	AD2884	Validation set 2		
68	AD2885	Validation set 2		
69	AD2887	Validation set 2		
70	AD2892	Validation set 2		
71	AD2883	Validation set 2		
72	AD2896	Validation set 2		
73	AD3055	Validation set 2		
74	AD3057	Validation set 2		
75	AD3061	Validation set 2		
76	AD3063	Validation set 2		
77	AD3065	Validation set 2		
78	AD3069	Validation set 2		
79	AD3071	Validation set 2		
80	AD3073	Validation set 2		
81	AD3075	Validation set 2		
82	AD3077	Validation set 2		
83	AD3081	Validation set 2		
84	AD3083	Validation set 2		
85	AD3085	Validation set 2		
86	AD3087	Validation set 2		
87	AD3089	Validation set 2		
88	AD3091	Validation set 2		
89	AD3093	Validation set 2		
90	AD3095	Validation set 2		
91	AD3097	Validation set 2		

92	AD3099	Validation set 2		
93	AD3101	Validation set 2		
94	AD3103	Validation set 2		
95	AD3105	Validation set 2		
96	AD3107	Validation set 2		
97	AD3109	Validation set 2		
98	AD3111	Validation set 2		
99	AD3113	Validation set 2		
100	AD3115	Validation set 2		
101	AD3117	Validation set 2		
102	AD3119	Validation set 2		
103	AD3121	Validation set 2		
104	AD3123	Validation set 2		
105	AD2894	Validation set 2		
106	AD3127	Validation set 2		
107	AD3129	Validation set 2		
108	AD3131	Validation set 2		
109	AD3133	Validation set 2		
110	AD3135	Validation set 2		
111	AD3137	Validation set 2		
112	AD3139	Validation set 2		
113	AD3141	Validation set 2		
114	AD3143	Validation set 2		
115	AD3145	Validation set 2		
116	AD3147	Validation set 2		
117	AD3149	Validation set 2		
118	AD3151	Validation set 2		
119	AD3153	Validation set 2		
120	AD3155	Validation set 2		
121	AD3157	Validation set 2		
122	AD3159	Validation set 2		
123	AD3161	Validation set 2		
124	AD3163	Validation set 2		
125	AD2895	Validation set 2		
126	AD2897	Validation set 2		
127	AD3167	Validation set 2		
128	AD3171	Validation set 2		
129	AD3173	Validation set 2		
130	AD3177	Validation set 2		
131	AD3179	Validation set 2		
132	AD3181	Validation set 2		
133	AD3183	Validation set 2		
134	AD3185	Validation set 2		
135	AD3187	Validation set 2		
136	AD3189	Validation set 2		
137	AD3191	Validation set 2		
138	AD3195	Validation set 2		

139	AD3197	Validation set 2		
140	AD3199	Validation set 2		
141	AD3201	Validation set 2		
142	AD3203	Validation set 2		
143	AD3205	Validation set 2		
144	AD3207	Validation set 2		
145	AD3209	Validation set 2		
146	AD3211	Validation set 2		
147	AD3217	Validation set 2		
148	AD3219	Validation set 2		
149	AD3221	Validation set 2		
150	AD3223	Validation set 2		
151	AD3225	Validation set 2		
	Indicates the presence of the mentioned fusion transcript			
